# Proximity ligation strategy for the genomic reconstruction of microbial communities associated with the ectoparasite *Caligus rogercresseyi*

**DOI:** 10.1038/s41598-021-04485-0

**Published:** 2022-01-17

**Authors:** Diego Valenzuela-Miranda, Ana Teresa Gonçalves, Valentina Valenzuela-Muñoz, Gustavo Nuñez-Acuña, Ivan Liachko, Bradley Nelson, Cristian Gallardo-Escarate

**Affiliations:** 1 Interdisciplinary Center for Aquaculture Research (INCAR), University of Concepción, P. O. Box 160-C, Concepción, Chile; 2 GreenCoLab-Associação Oceano Verde, University of Algarve, Campus de Gambelas, Faro, Portugal; 3 Phase Genomics, Inc., Seattle, USA

**Keywords:** Functional genomics, Genome, Microbial genetics, Sequencing

## Abstract

The sea louse *Caligus rogercresseyi* has become one of the main constraints for the sustainable development of salmon aquaculture in Chile. Although this parasite's negative impacts are well recognized by the industry, some novel potential threats remain unnoticed. The recent sequencing of the *C. rogercresseyi* genome revealed a large bacterial community associated with the sea louse, however, it is unknown if these microorganisms should become a new focus of sanitary concern. Herein, chromosome proximity ligation (Hi-C) coupled with long-read sequencing were used for the genomic reconstruction of the *C. rogercresseyi* microbiota. Through deconvolution analysis, we were able to assemble and characterize 413 bacterial genome clusters, including six bacterial genomes with more than 80% of completeness. The most represented bacterial genome belonged to the fish pathogen *Tenacibacullum ovolyticum* (97.87% completeness), followed by *Dokdonia *sp. (96.71% completeness). This completeness allowed identifying 21 virulence factors (VF) within the *T. ovolyticum* genome and four antibiotic resistance genes (ARG). Notably, genomic pathway reconstruction analysis suggests putative metabolic complementation mechanisms between *C. rogercresseyi* and its associated microbiota. Taken together, our data highlight the relevance of Hi-C techniques to discover pathogenic bacteria, VF, and ARGs and also suggest novel host-microbiota mutualism in sea lice biology.

## Introduction

The sea louse, *Caligus rogercresseyi*, is a marine ectoparasite that has become one of the main constraints for developing sustainable aquaculture in Chile^1^. This copepod is the etiological agent of caligidosis, a prevalent and severe disease that has been established as one of the top priorities for Chilean salmon farming^2^. Lice infestation produces severe deleterious effects on the host, including a reduction in growth performance, a reduced feed-conversion efficiency, severe chronic stress, skin wounds, and loss of scales that increase the susceptibility of the infested salmonids to secondary infections^3,4^. Currently, sea lice control strategies are mainly based on pharmacological treatments, increasing the average production costs by $1.4 US/kg^5^. Besides this well-known negative impact, novel evidence suggests the presence of a large and diverse microbiota associated with *C. rogercresseyi*^6^. However, the biological role of these microorganisms or their potential importance on salmon aquaculture remains largely elusive.

Over the past years, the idea that microbes play pivotal roles in the development of almost all living organisms has gain consensus^7^. Microbes can drive physiological and evolutionary changes due to their interaction with their host^8^. Particularly in parasites, the microbiota can actively participate during the infective process by producing toxins, redirecting the host immune system, or increasing the parasite’s virulence^7,9,10^. Herein, the parasite's microbiota has been proposed as a target to develop alternative therapeutic strategies to cope with infestations^11,12^.

Vector-borne diseases are another major issue regarding parasite-associated microbes in arthropods, responsible for transmitting virus, bacteria, and protozoa among vertebrate hosts^13^. Copepods are widely colonized by bacteria^14,15^, among which pathogenic species are usually identified^16,17^. Known fish pathogens have been found in other caligid parasitic copepods, such as *Vibrio* sp. in *Caligus lalandei*^18^. Likewise, it has been suggested that the sea louse *Lepeophtheirus salmonis* could act as a vector of several diseases, including infectious hematopoietic necrosis virus (IHNv), the infectious salmon anemia virus (ISAv), and furunculosis^19–21^. Our research group previously identified bacterial pathogens present in *C. rogercresseyi* associated microbiota, including *Vibrio*, *Tenacibaculum*, and *Aeromonas* genus^6^. However, these species' genomic features are poorly understood, hindering the biological role of the sea lice microbiota and its potential impact on salmon aquaculture.

High-throughput chromosomal conformation capture (Hi-C) coupled with long-read sequencing has offered the possibility to deeply understand microbiomes by reconstructing individual bacterial genomes from complex microbe samples^22–24^. However, this approach has been mainly applied in the study of mammalian gut microbiota and it is less frequently used in marine environments. This work aimed to reconstruct the genetic background of sea lice microbiota to evaluate possible new threats for salmon aquaculture and to reveal novel insights about the biological roles of these bacteria in *C. rogercresseyi* biology. Through Hi-C and bioinformatic deconvolution, we were able to reconstruct different bacterial genome clusters, allowing the characterization of complete genomes of pathogenic salmon bacteria and identifying virulence factors (VF), and antibiotic resistance genes (ARG) present in sea lice microbiota. Our data also reveal that some bacteria associated with *C. rogercresseyi* might be fulfilling key metabolic roles, suggesting novel host-microbiota mutualism in sea lice biology.

## Results

**Figure 1:**
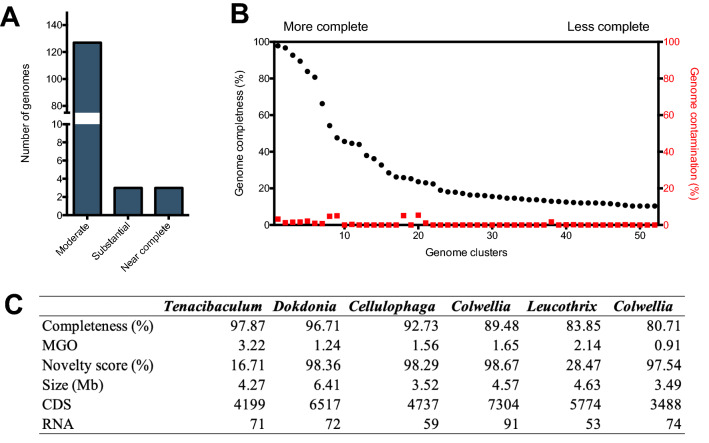
Summary of the genomic reconstruction of bacterial clusters associated with *Caligus rogercresseyi*. (**A**) Genome cluster classification according with their completeness in near complete (> 95%), substantial (> 80%), and moderate (< 80%). (**B**) Genome completeness and contamination among the 50 most represented bacterial clusters. (**C**) Main genomic features of the top 5 represented genomes clusters in sea lice microbiota.

Organism-associated microbiota can fulfill key roles in the development of almost all living organisms. In parasites, this role can comprise both biological and functional roles during pathogenesis. Hi-C sequencing and a bioinformatic deconvolution analysis were used to gain insights about sea lice microbiota's relevance in aquaculture. Through this analysis, we were able to assemble and characterize 413 bacterial genome clusters. These clusters were then analyzed for completeness and genetic contamination, based on the marker gene overrepresentation (MGO). Based on their completeness, the genomes were classified as near-complete (100% to 95%), substantial complete (95–85%), and moderate complete (< 85%). The Hi-C sequencing and deconvolution analysis revealed that two genome clusters accounted for over 95% completeness, two others were substantial complete with around 83 and 89% completeness, and with the more significant number of clusters classified as moderate complete (Fig. 1A). Regarding gene overrepresentation, the analysis evidenced a low degree of genetic cross-contamination among bacterial clusters, with no values above 4% (Fig. 1B). Genomic clusters with the most completeness comprised five different genera, including *Tenacibacullum, Dokdonia, Cellulophaga, Colwellia,* and *Leucothrix* (Fig. 1C). The genome cluster belonging to the known fish pathogen *Tenacibacullum* reached over 97% completeness with an estimated genome size of 4.27 Mb. Interestingly, this approach allowed the genome reconstruction of a relatively large bacterial genome (> 6.41 Mb) with estimated completeness of over 96%. Compared with the RefSeq database, some of the genome clusters accounted for a novelty score above 95% (*Dokdonia*, *Cellulophaga,* and *Colwellia*), thus evidencing that this approach also allowed the reconstruction of previously unknown bacterial genomes.

**Figure 2:**
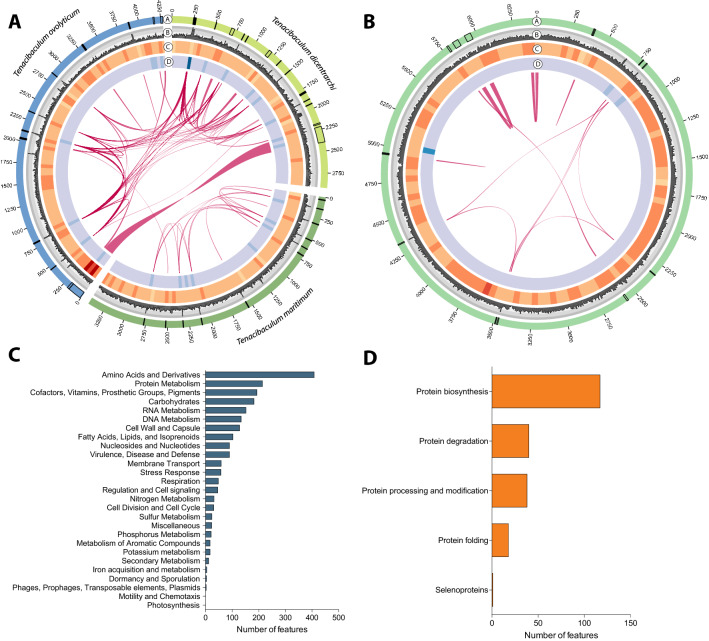
General overview of *Tenacibaculum* and *Dokdonia* sp. genomes. (**A**) Circos^65^ plot representing different *Tenacibaculum* genomes including the genome size (a), GC content (b), CDS density (c), ncRNA density (d) and synteny blocks (ribons). (**B**) Genome characterization of *Dokdonia sp.* cluster. Circos^65^ plot representing *Dokdonia sp.* genome including the genome size (a), GC content (b), CDS density (c), ncRNA density (d) and synteny blocks (ribons). (**C**) Functional annotation of the CDS characterize in *Dokdonia* sp. genome provided by RAST annotation. (**D**) Functional annotation of the CDS within amino acids metabolism found in *Dokdonia* sp. genome.

We further explored the *Tenacibacullum* cluster, as it is a genus known to comprise different pathogenic species that are relevant for Chilean aquaculture^25^. Based on all the 16S genes fully annotated in the genome, we were able to identify this particular cluster as *T. ovolyticum* (Fig. S1). The circos plot, including different features such as GC content, coding, and non-coding gene density, was constructed to gain a general idea about T. ovolyticum genome structure (Fig. 2A). Comparative genome analyses among *T. dicentrarchi*, *T. maritimum*, and *T. ovolyticum* showed that different synteny blocks between *T. dicentrarchi* and *T. maritimum* are shared with *T. ovolyticum*. Notably, more synteny conservation was observed between *T. ovolyticum* and *T. dicentrarchi* (Fig. 2A). Given the degree of completeness, we further explored the presence of virulence factors (VFs) in the *T. ovolyticum* genome. Using the Virulence Factor Database (VFDB) as a reference, we were able to identify 21 genes present in the *T. ovolyticum* genome, including genes belonging to the Dot/Icm type IVB secretion system, 60 K heat shock proteins (HtpB), and catalases (katA) genes among others (Table 1). Moreover, using the MEGARes database, the results evidenced antibiotic resistance genes (ARGs) within all the bacterial genome clusters. Through this analysis, it was possible to identify 4 ARGs, including the *Tetracycline efflux Na*^+^*/H*^+^ *transporter tet(35)*, *Plasmid-mediated quinolone resistance protein (QnrS2)*, *Integron chloramphenicol acetyltransferase (catB9),* and *Oxa beta-lactamase (Oxa-209)* genes (Table 1).

**Table 1:** Genes coding for virulence factors (VF) and antibiotic resistance genes identified in the genomes associated with *C. rogercresseyi*.

Gene	Description	E-value	Closely related specie
**VF**			
basC	Acinetobactin biosynthesis protein	3.53E−100	*Acinetobacter baumannii*
basG	Acinetobactin biosynthesis protein	1.39E−109	*Acinetobacter baumannii*
bplC	Lipopolysaccharide biosynthesis protein	1.49E−107	*Bordetella pertussis*
bplC	Lipopolysaccharide biosynthesis protein	2.85E−101	*Bordetella pertussis*
bplD	UDP-N-acetylglucosamine 2-epimerase	6.00E−124	*Bordetella pertussis*
bplD	UDP-N-acetylglucosamine 2-epimerase	9.67E−114	*Bordetella pertussis*
bplE	Probable glycosyl transferase	2.23E−121	*Bordetella pertussis*
cap8D	Capsular polysaccharide synthesis enzyme	3.77E−114	*Staphylococcus aureus * subsp. *aureus*
CBU_0270	Dot/Icm type IVB secretion system	4.45E−169	*Coxiella burnetii*
clpC	Endopeptidase Clp ATP-binding chain C	0	*Listeria monocytogenes*
clpC	Endopeptidase Clp ATP-binding chain C	0	*Listeria monocytogenes*
clpC	Endopeptidase Clp ATP-binding chain C	1.25E−112	*Listeria monocytogenes*
galE	UDP-glucose 4-epimerase	5.83E−103	*Haemophilus influenzae*
htpB	Hsp60, 60 K heat shock protein HtpB	0	*Legionella pneumophila * subsp. *pneumophila*
htpB	Hsp60, 60 K heat shock protein HtpB	0	*Legionella pneumophila * subsp. *pneumophila*
katA	Catalase	0	*Legionella pneumophila * subsp. *Pneumophila*
katA	Catalase	0	*Legionella pneumophila * subsp. *pneumophila*
pilR	Two-component response regulator	6.78E−104	*Pseudomonas aeruginosa*
relA	Probable GTP pyrophosphokinase	1.03E−136	*Mycobacterium tuberculosis*
rffG	dTDP-glucose 46-dehydratase	4.95E−113	*Haemophilus influenzae*
tviB	Vi polysaccharide biosynthesis protein	1.88E−160	*Salmonella enterica subsp. enterica*
**ARG**			
tet(35)	Tetracycline efflux Na+/H+ transporter	0	*Vibrio harveyi*
QnrS2	Plasmid-mediated quinolone resistance protein	3.13E−151	*Salmonella enterica*
catB9	Integron chloramphenicol acetyltransferase	2.03E−77	*Vibrio cholerae*
OXA-209	Oxa beta lactamase	2.52E−71	*Riemerella anatipestifer*

**Figure 3:**
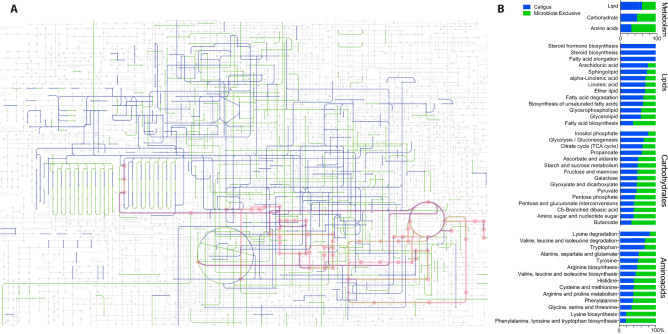
(**A**) Graphical representation of the metabolic pathways reconstructed for *Caligus rogercresseyi* (blue) and its associated microbiome (green). The amino acid metabolism is highlighted in red. (**B**) Percentage of genes annotated for the different metabolic pathways encoded in the sea lice genome (blue) and that were exclusively encoded in the microbiome (green). All pathways were constructed using KEGG pathways as reference^66^.

**Figure 4:**
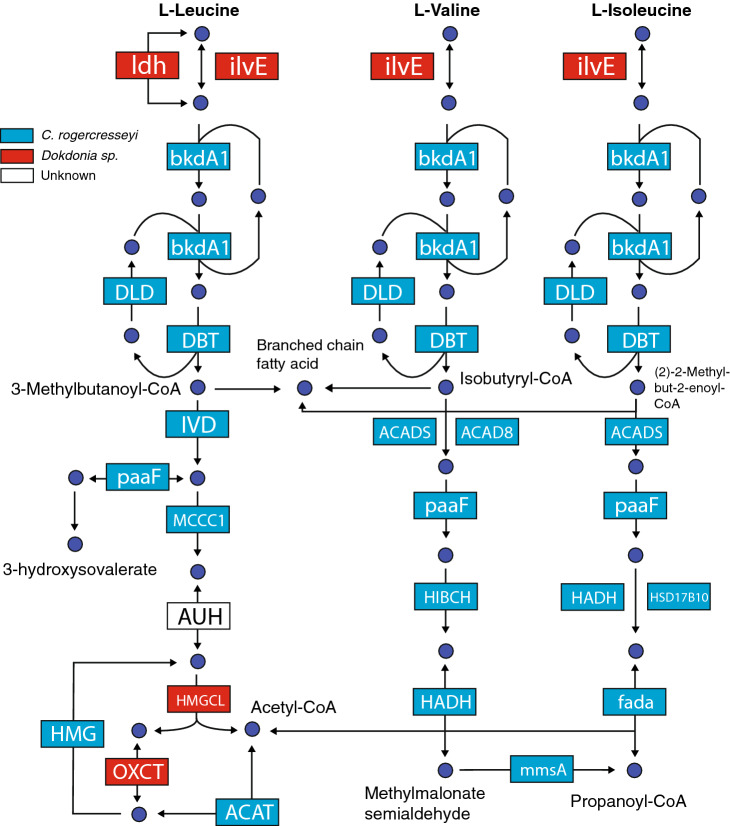
Metabolic reconstruction of the leucine, valine and isoleucine metabolism. Each box represents a gene coding for a protein in the pathway, where the blue boxes represent genes found in *C. rogercresseyi* genome and the red boxes genes found just in *Dokdonia* sp. Genome.

The ubiquitous sets of genes determine genome completeness, and a single copy within a phylogenetic lineage could be used as a proxy for bacterial abundance in metagenomic studies. As one of the largest and more complete genomes represented in sea lice microbiota, we explored the *Dokdonia* sp. genome, representing their main features through a circos plot (Fig. 2B). The genome size of *Dokdonia* sp*.* was estimated in 6.41 Mb, accounting for 6517 CDS and 72 RNAs. The functional annotation evidence a large number of genes associated with different metabolism features, including protein, amino acids, and derivates metabolism, followed by cofactors, vitamins, pigments, and others (Fig. 2C). Most of the genes were annotated in protein biosynthesis, degradation, processing, and modification (Fig. 2D). Given the large number of protein metabolism genes found in the *Dokdonia* sp. genome, and the recent availability of the sea lice genome (ASM1338718v1)^26^, we further investigate possible metabolic complementation between sea lice and its associated microbiota. To do this, the Kyoto Encyclopedia of Genes and Genomes (KEGG) database was used as a reference to annotate the genes in different metabolic pathways, including lipid, carbohydrate, and amino acid metabolism (Fig. 3A). The results evidenced that the more significant number of metabolic genes annotated in the lice microbiota but not present in the sea louse genome belongs to the amino acid metabolism, followed by carbohydrate and lipid metabolism (Fig. 3B). Regarding specific pathways mostly encoded by parasite microbiota, the fatty acid biosynthesis, butanoate metabolism, phenylalanine, tyrosine, and tryptophan biosynthesis were identified for lipid, carbohydrate, and amino acid metabolism, respectively (Fig. 3B). The complete list of all the metabolic KEGG pathways analyzed can be found in the supplementary material 1. As an example, we analyzed the branched chain amino acid (BCA) metabolism (Fig. 4). The results evidenced that the genome of *C. rogercresseyi* contains almost all the genes coding for this pathway. However, key biosynthetic genes, including *Idh*, *iLve*, *HMGCL,* and *OXCT* were absented or not identified. On the other hand, we were able to find analog genes to *Idh*, *iLve*, *HMGCL,* and *OXCT* in the metagenome. Although it is not possible to confirm metabolic complementation between sea lice and its microbiota, these results could suggest novel insights about possible roles of sea lice-associated microbiota in parasite biology.

## Discussion

Marine organisms constantly interact with microbial communities present in the environment^27^. While some of these bacteria might fulfill beneficial roles in one species, they can also become harmful in other ones. Therefore, this study aimed to explore possible roles of sea lice-associated microbiota in *C. rogercresseyi* biology and its potential risk to salmon aquaculture. Previous efforts have been made to understand the microbial communities associated with the northern hemisphere salmon louse (*Lepeophtheirus salmonis*)^28,29^. However, these approaches were technically limited based on the need to isolate and culture each bacteria species that want to be identified or the lack of taxonomical resolution at the species or even family level.

Long nanopore reads and computational tools have recently allowed the full-length 16s rRNA sequencing of the unraveled sea lice-associated microbiota, evidencing the geographical variations and the core microbiota at species-specific levels^6^. Here, chromosome proximity ligation (Hi-C) coupled with long-read sequencing is a valuable tool to reconstruct individual bacterial genomes, uncovering novel insights associated with the sea lice microbiome. Thus, our results evidenced the assemble of almost complete genomes from a complex sample with low degrees of cross-contamination. The complete genomes belonged to species within the *Tenacibacullum* and *Dokdonia* genus, previously described as abundant components of salmon and sea lice microbiota^6,28,29^. As expected, our results evidence that each bacterial genome cluster's completeness degree might be directly related to the abundance of each bacteria within the sample. Although the estimation of each bacteria's abundance was not a goal in this research, the inclusion of a known number of a particular bacterium within the sample can be employed to estimate bacterial abundance using Hi-C data^24^.

It is known that ectoparasites can become vectors of different pathogens^18,30^. Previously, it has been suggested that the northern salmon lice *L. salmonis* might fulfill roles as a vector for the transmission of the infectious hematopoietic necrosis virus (IHNV) and the *Aeromonas salmonicida *subsp. *Salmonicida*^31,32^. However, the role of *C. rogercresseyi* as a vector for fish pathogens is less clear. Given the genome completeness degree, our results confirm previous research evidencing *Tenacibaculum* genus as an abundant component of *C. rogercresseyi* microbiota^6^. Over the last years, this genus has gained major importance as an emergent pathogen causing different outbreaks in salmon aquaculture with presence in Chile, Norway, and Australia^25,33–35^. Although no evidence has been found about sea lice's role in the transmission of the salmon pathogen *Piscirickettsia salmonis*^36^, its potential as a transmission vector for *Tenacibaculum* species needs to be further investigated.

Beyond identifying bacterial pathogens, our approach allowed the characterization of 4911 coding genes in the *T. ovolyticum* genome. This information can become key for the application of reverse vaccinology, which has been used to cope with vector-borne parasite diseases^37^. The use of genomic information allows the rapid discovery of potential antigens, being a promising methodology for developing new fish vaccines^38^. Virulence factors were also identified in the *T. ovolyticum* genome, including the Dot/Icm type IVB secretion system, 60 K heat shock proteins (HtpB), and catalases (katA) genes, among others, which has been suggested as key factors during the infection of different fish pathogens^39–42^. Thus, this genomic information availability can also be used to infer pathogenic mechanisms employed by potential fish pathogens present in *C. rogercresseyi* microbiota.

Antibiotic resistance is another major issue both for human health and aquaculture. Therefore, using the ARG database, we identified four different ARGs present in the genome clusters of sea lice microbiota, including *tet(35), QnrS2, catB9,* and *OXA-209*. It has been shown that *tet(35)* gene encodes for an efflux pump that confers resistance against tetracyclines^43^, *QnrS2* is a plasmid-mediated protein that confers resistance against quinolones^44^, *catB9* is a chromosome-encoded chloramphenicol resistance protein^45^ while *OXA-209* gene encodes a beta-lactamase resistance protein^46^. Tetracyclines, quinolones, chloramphenicol, and beta-lactamases antibiotics have been commonly used in aquaculture^45,47,48^. Particularly in Chile, it was estimated that between 2011 and 2015, the antibiotics used to produce 1 ton of salmon were 1,500 times higher than in Norway^49^. Although this number has been decreased over the last years, florfenicol and oxytetracycline are still the most used antibiotics in Chilean salmon aquaculture^50^. Here, the presence of *tet(35)* on sea lice-associated microbiota should raise a major concern for the industry since it has been shown that this resistance determinant found from aquaculture sources can be transferred between bacterial species^51^. Thus, there is a potential risk that these genes could be transferred between bacteria species composing the sea lice microbiota, including some fish pathogens that can later infect salmons and reduce the efficacy of the currently used antibiotic treatments.

It is known that arthropods-associated microbiota can play pivotal roles in their host development^52,53^. Here, metabolic interdependence is a widespread phenomenon between bacteria and arthropods that have been previously reported (reviewed by Zientz et al.^53^). These interactions have been identified for different metabolic pathways, including glycolysis, gluconeogenesis, phospholipids, nucleotide, and sulfur metabolism^53^. Our results evidenced that the genome of C. rogercresseyi lacks different metabolic-related genes, which are putatively encoded in the microbiome. Amino acid metabolism was the most extensive set of microbiota-exclusive genes, with phenylalanine, tyrosine, tryptophan, and lysine biosynthesis the most supported pathways. The absence of these genes in the *C. rogercresseyi* genome suggests that sea lice cannot biosynthesize these amino acids; therefore, they should be obtained through feeding. However, another possibility is that *C. rogercresseyi* might complement the metabolic machinery present in its core associated microbiota to obtain these amino acids. This phenomenon is well-known in aphid parasites. Previous reports have suggested that the bacteria *Buchnera aphidicola* provides essential amino acids to its insect host^54^. Likewise, through RNA-seq and pathway analysis, researchers have found that aphid host can upregulates genes in order to fill the gaps of Buchnera’s essential amino acid pathways^55^. On the other hand, the whole-genome sequencing of the symbiont bacteria *Sulcia muelleri* on the insect *Homalodisca coagulate* revealed that the bacteria produced most of the essential amino acids needed by its host^56^. Moreover, it has been shown that the deprivation of some bacteria in arthropods through antibiotics negatively impacts the host's development and survival^57^. While the nutritional requirements of the sea lice are barely known, our approach suggest the existence of essential amino acids in sea lice, that in turn, are encoded in the microbiome.

Through full 16S nanopore sequencing we have recently identify a core microbiome from sea lice collected from different geographical areas in Chile^6^. Among the identified species, it was suggested that *Dokdonia* sp*.* could fulfill roles during the infective process of sea lice because of the secretory capacity of bioactive compounds from this bacteria^6^. Notably, one of the most represented genomes in the sea lice microbiome was *Dokdonia* sp. Here, the pathway analysis evidenced that the genome of *Dokdonia* sp*.* encodes for genes that can fill the gaps for the biosynthesis and degradation of valine, leucine, and isoleucine. Although further studies are needed to confirm the metabolic interdependency between the sea lice and its associated microbiota, a strategy targeting some bacteria like *Dokdonia* sp. might provide novel therapeutic tools to mitigate the parasite's impacts in salmon aquaculture.

## Methods

### Sample collection and sequencing

Adult specimens of *C. rogercresseyi* were collected in spring from a commercial salmon farm located in Los Lagos, Chile. The site selection was based on our previous established criteria^6^, prioritizing cages with more than 8 months in seawater and centers with less or no pharmacological (both delousing and antibiotics) usage. Collected lice were treated with different antibiotics to minimize environmental bacteria's presence as previously standardized^6^. Thus, adult sea lice were treated with a solution containing 20 mg/ml ampicillin (Sigma-Aldrich, USA), 20 mg/ml Kanamycin (US biological, USA), 1× Penicillin–Streptomycin (GIBCO, USA), and 100 μg/ml Primocin (Invivogen, USA). After 72 h, the specimens were transferred to tubes containing molecular grade EtOH 100% and stored at – 80 °C for further processing. Genomic DNA was isolated from a pool of 10 individuals (5 females and 5 males) using the Qiagen DNA purification kit (QIAGEN, Germantown, MD, USA) according to manufacturer′s instructions. DNA quality and integrity were assessed through a 1% agarose gel and NanoDrop 1000 Spectrophotometer. Samples with no smear and absorbance ratios above 1.8 were used for further processing. Shotgun metagenomic libraries were prepared using the TruSeq® DNA PCR-Free Library Prep kit (Illumina, San Diego, CA, USA), while Hi-C sequencing libraries were prepared using the Phase Genomics’ Animal Hi-C kit (Phase Genomics, Seattle, WA, USA) following the manufacturer′s instructions. Both short-read sequencing libraries and Hi-C libraries were sequenced on a HiSeq 4000 (Illumina, San Diego, CA, USA).

### Sequencing data analysis

Sequencing analysis and processing were performed as previously described^24^. Briefly, sequencing adapters from the shotgun metagenomic sequencing data were removed using BBDuk (BBTools developed by the Joint Genome Institute). Sequence reads were mapped against the last version of the sea lice genome^58^ using the BWA-MEM alignment tool. Unmapped reads were de novo assembled using Megahit^59^ and assessed through MetaQuast^60^ considering default parameters. The Hi-C reads were mapped against the de novo assembly through the Burrows–Wheeler alignment tool BWA-MEM^61^. The deconvolution of contigs was performed as previously described^24^. Contigs with less than two restriction sites and shorter than 1000 bp in sizer were removed, whereas the remaining contigs were grouped into genome clusters considering the Hi-C contact data using a proprietary Markov Chain Monte Carlo algorithm. All assembled cluster were deposited on figshare^62^.

### Genome clusters characterization and annotation

The first quality control of the generated genome clusters was assessed using CheckM^63^, which provides a robust estimation of genome completeness and contamination by accounting for ubiquitous genes single-copy within a phylogenetic lineage. Thus, genome clusters with marker gene overrepresentation (MGO) over five were discarded from subsequent analysis. To estimate genome novelty, each metagenome-assembled genomes (MAGs) were compared with the RefSeq genome database using Mash^64^. Thus, a genome was considered as “novel” when its > 70% complete, < 10% marker gene overrepresentation and > 90% novelty score. On the other hand, a “known” genome is defined when > 70% completeness, < 10% marker gene overrepresentation and < 90% novelty score. Genome annotation was performed using the Rapid Annotations using Subsystems Technology (RAST) servers considering default parameters. For each genome cluster, the coding sequences (CDS) were annotated, and BLAST was used for the annotation of virulence factors (VF) and antibiotic resistance genes (ARG) considering as reference the virulence factor database (VFDB) and the MEGARes database respectively. Individual genome cluster features such as GC content, CDS, and RNA density was calculated by a 10,000 bp frame using R. Synteny blocks between closely related species were detected using the Synteny Block ExpLoration tool (Siberia) (http://bioinf.spbau.ru/sibelia) and plotted with Circos software version 0.69-6 (http://circos.ca/)^65^.

### Metabolic pathway analysis

The CDS obtained from the different bacterial clusters were compared with the genes annotated in the last version of the *C. rogercresseyi* genome^58^ to predict possible biological roles of sea lice microbiota. All the CDS were translated to proteins using the CLC genomic workbench (V10, Qiagen). The predicted proteins for both *C. rogercresseyi* and the bacterial clusters were annotated with KEGG Automatic Annotation Server (KAAS)^66^ through a bi-directional best hit (BBH) method to assign orthologs and selecting GHOST X search engine. The resulting pathways were then compared to find metabolic complementarity between the sea lice and the genome clusters composing in sea lice microbiota.

## Conclusion

Proximity ligation techniques for the reconstruction of microbial communities in aquaculture provide valuable information for understanding the biological role and potential risk of the sea lice microbiota. Herein, we provide a novel approach to reveal genome features of some bacteria associated with the sea lice, allowing the identification of pathogenic bacteria, virulence factors, antibiotic resistance genes, and the reconstruction of bacterial metabolic pathways associated with *C. rogercresseyi*. We believe that our findings should raise awareness about sea lice microbiota as a pivotal component in salmon aquaculture, providing novel perspectives to develop novel control strategies.

## Supplementary Information

Supplementary Figure S1.

Supplementary Information 2.
