# Author Correction: Proximity ligation strategy for the genomic reconstruction of microbial communities associated with the ectoparasite *Caligus rogercresseyi*

**DOI:** 10.1038/s41598-022-11969-0

**Published:** 2022-05-09

**Authors:** Diego Valenzuela-Miranda, Ana Teresa Gonçalves, Valentina Valenzuela-Muñoz, Gustavo Nuñez-Acuña, Ivan Liachko, Bradley Nelson, Cristian Gallardo-Escarate

**Affiliations:** 1 Interdisciplinary Center for Aquaculture Research (INCAR), University of Concepción, P. O. Box 160-C, Concepción, Chile; 2 GreenCoLab-Associação Oceano Verde, University of Algarve, Campus de Gambelas, Faro, Portugal; 3 Phase Genomics, Inc., Seattle, USA

Correction to: *Scientific reports* 10.1038/s41598-021-04485-0, Published online 17 January 2022

The Supplementary Information published with this Article contained errors.

Bootstrap values were omitted from the phylogenetic tree in Supplementary Figure S1 as was a description of its construction. The original Supplementary Figure S1 appears below.

These error have now been corrected in the Supplementary Figure S1 file that accompanies the original Article.

## Supplementary Information

Supplementary Information.

