# Supplementary material for: Proximity ligation strategy for the genomic reconstruction of microbial communities associated with the ectoparasite Caligus rogercresseyi
Source: Sci Rep. 2022 Jan 17;12:783. doi: 10.1038/s41598-021-04485-0 (PMC8764032; doi:10.1038/s41598-021-04485-0)
Supplement: Supplementary file 1 — Supplementary Figure S1. [file 41598_2021_4485_MOESM1_ESM.pdf]

Supplementary figure 1

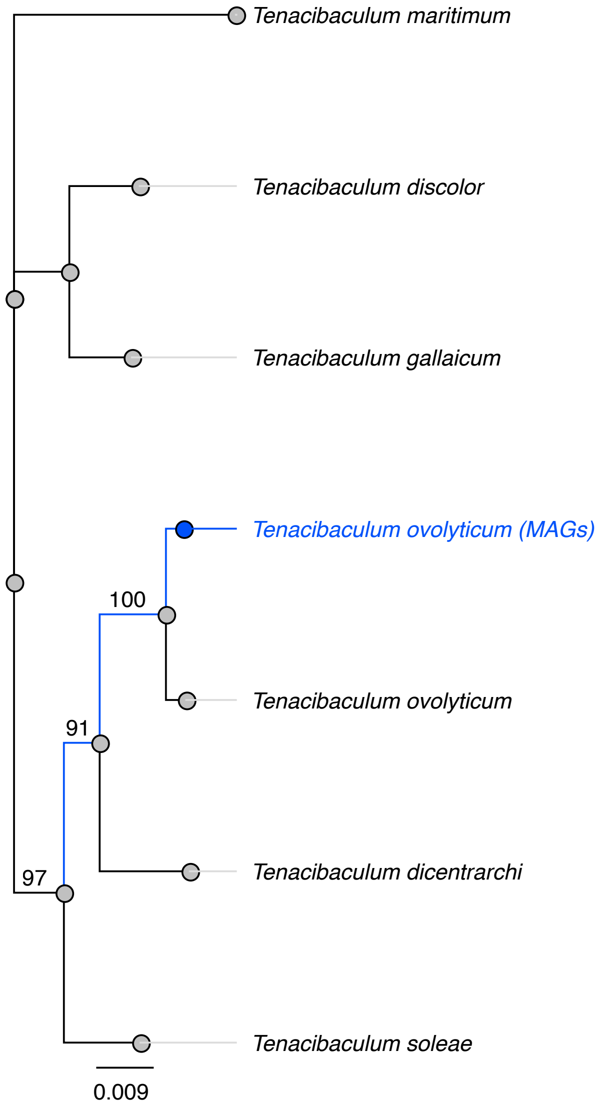

Figure S1. A phylogenetic tree was constructed using the 16S gene from 6 Tenacibaculum species and the identified *T. ovolyticum* MAGs. The data analysis was conducted using Geneious prime (2019.2.1). First the sequences were aligned using the Geneious Alignment, considering a global alignment with free end gaps; a Gap open penalty: 12; Gap extension penalty: 3; and refinement iterations: 2. This alignment was used to construct a phylogenetic tree using the Geneious Tree Builder, considering a Jukes-Cantor genetic distance model and a Neighbor-Joining as tree build method. The consensus tree was constructed using bootstrap as resampling method with 100 replicates. Bootstrap values above 85% were included in the tree.
