# Supplementary material for: Proximity ligation strategy for the genomic reconstruction of microbial communities associated with the ectoparasite Caligus rogercresseyi
Source: Sci Rep. 2022 Jan 17;12:783. doi: 10.1038/s41598-021-04485-0 (PMC8764032; doi:10.1038/s41598-021-04485-0)
Supplement: Supplementary file 2 — Supplementary Information 2. [file 41598_2021_4485_MOESM2_ESM.pdf]

Supplementary material 1

Below are described different metabolic KEGG pathways for amino acid, carbohydrate and lipid metabolism. The boxes represent a particular gene in a reaction. Blue boxes represent genes annotated in the genome of the sea lice *Caligus rogercresseyi*. Green boxes represent genes annotated just in the microbiome of *C. rogercresseyi*. White boxes represent genes that were not found either in the genome of the parasite or this microbiome

Amino Acid metabolism

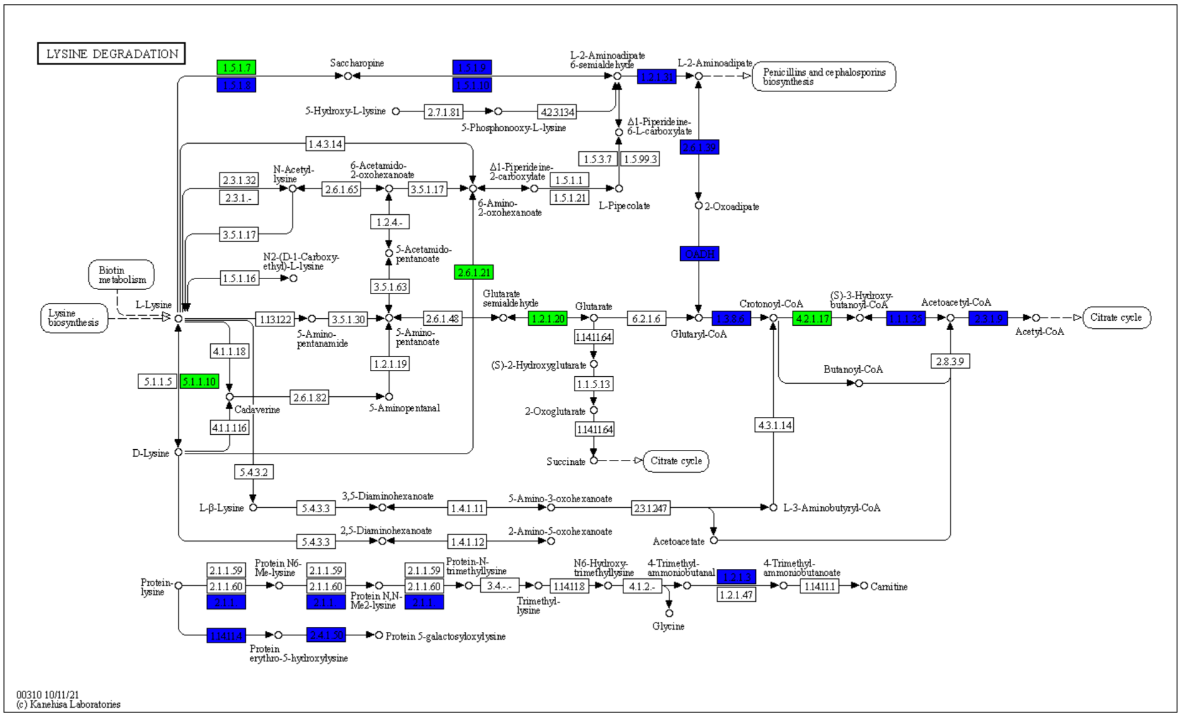

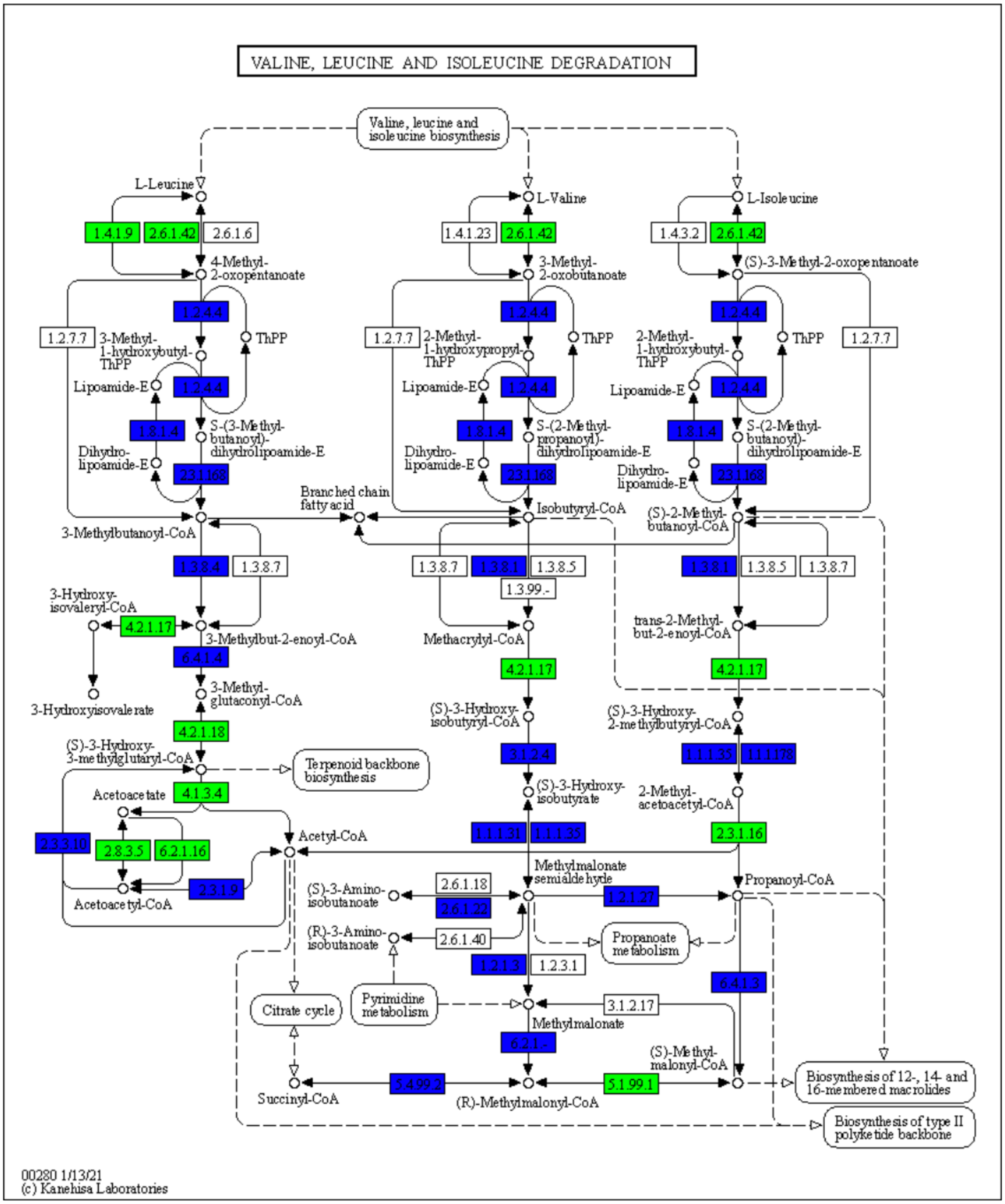
VALINE, LEUCINE AND ISOLEUCINE DEGRADATION
Valine, leucine and isoleucine biosynthesis
L-Leucine
L-Valine
L-Isoleucine
1.4.1.9
2.6.1.42
2.6.1.6
1.4.1.23
1.4.3.2
4-Methyl-2-oxopentanoate
3-Methyl-2-oxobutanoate
(S)-3-Methyl-2-oxopentanoate
1.2.4.4
ThPP
1.2.7.7
3-Methyl-1-hydroxybutyl-ThPP
2-Methyl-1-hydroxypropyl-ThPP
2-Methyl-1-hydroxybutyl-ThPP
Lipoamide-E
1.8.1.4
S-(3-Methylbutanoyl)-dihydrolipoamide-E
S-(2-Methylpropanoyl)-dihydrolipoamide-E
S-(2-Methylbutanoyl)-dihydrolipoamide-E
Dihydrolipoamide-E
2.3.1.168
Branched chain fatty acid
3-Methylbutanoyl-CoA
Isobutyryl-CoA
(S)-2-Methylbutanoyl-CoA
1.3.8.4
1.3.8.7
1.3.8.1
1.3.8.5
1.3.99.-
3-Hydroxyisovaleryl-CoA
4.2.1.17
3-Methylbut-2-enoyl-CoA
Methacrylyl-CoA
trans-2-Methylbut-2-enoyl-CoA
6.4.1.4
3-Hydroxyisovalerate
3-Methylglutaconyl-CoA
4.2.1.18
(S)-3-Hydroxyisobutyryl-CoA
(S)-3-Hydroxy-2-methylbutyryl-CoA
(S)-3-Hydroxy-3-methylglutaryl-CoA
Terpenoid backbone biosynthesis
3.1.2.4
1.1.1.35
1.1.1.178
Acetoacetate
4.1.3.4
(S)-3-Hydroxyisobutyrate
2-Methylacetoacetyl-CoA
2.3.3.10
2.8.3.5
6.2.1.16
Acetyl-CoA
1.1.1.31
2.3.1.16
2.3.1.9
Acetoacetyl-CoA
Methylmalonate semialdehyde
(S)-3-Aminoisobutanoate
2.6.1.18
2.6.1.22
1.2.1.27
Propanoyl-CoA
(R)-3-Aminoisobutanoate
2.6.1.40
Propanoate metabolism
1.2.1.3
1.2.3.1
6.4.1.3
Citrate cycle
Pyrimidine metabolism
3.1.2.17
Methylmalonate
6.2.1.-
(S)-Methylmalonyl-CoA
Biosynthesis of 12-, 14- and 16-membered macrolides
Succinyl-CoA
5.4.99.2
(R)-Methylmalonyl-CoA
5.1.99.1
Biosynthesis of type II polyketide backbone
00280 1/13/21
(c) Kanehisa Laboratories

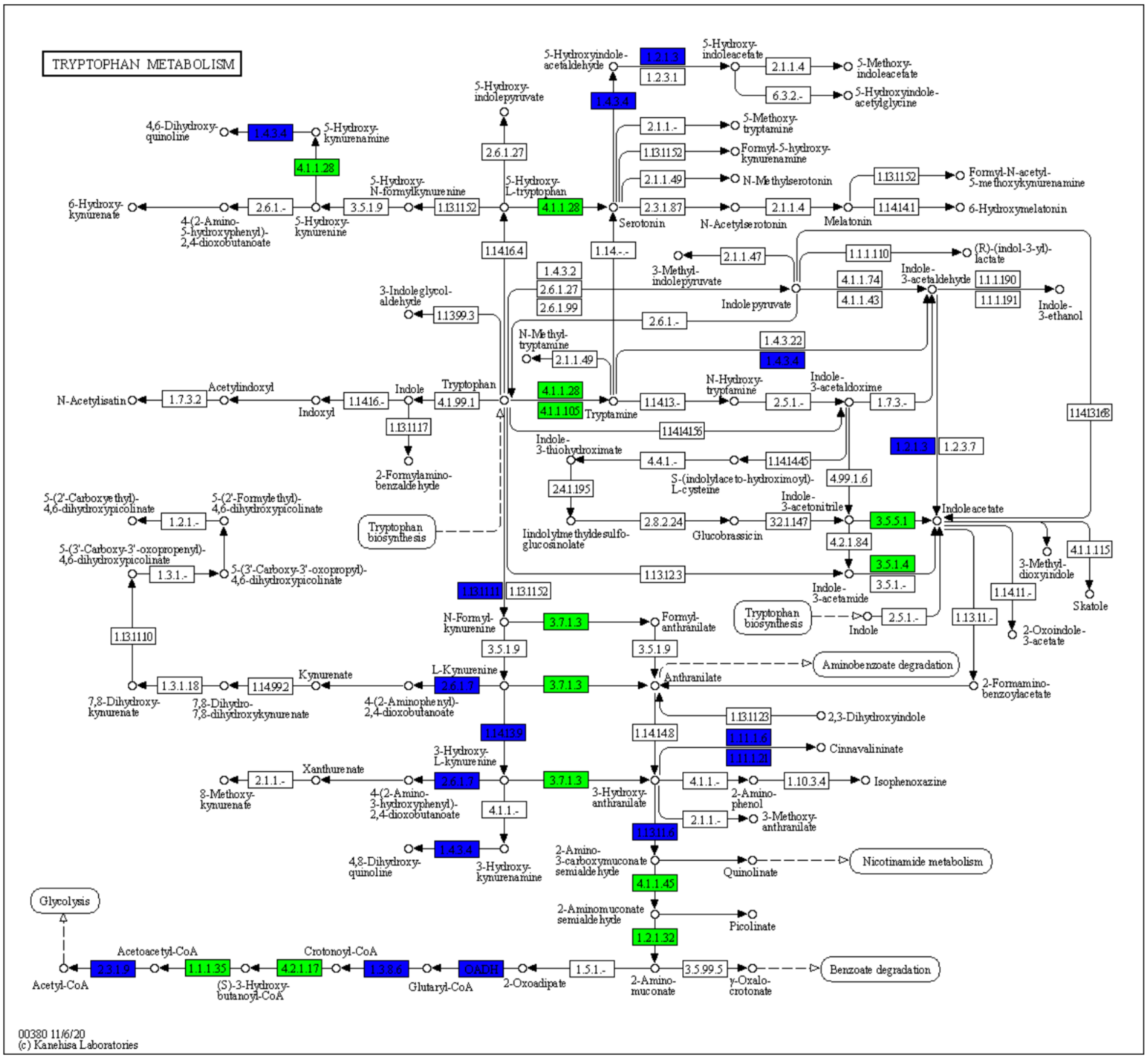

TRYPTOPHAN METABOLISM
5-Hydroxyindole-acetaldehyde
1.2.1.3
1.2.3.1
5-Hydroxy-indoleacetate
2.1.1.4
5-Methoxy-indoleacetate
6.3.2.-
5-Hydroxyindole-acetylglycine
1.4.3.4
5-Hydroxy-indolepyruvate
4,6-Dihydroxy-quinoline
5-Hydroxy-kynurenamine
2.6.1.27
2.1.1.-
5-Methoxy-tryptamine
1.13.11.52
Formyl-5-hydroxy-kynurenamine
4.1.1.28
2.1.1.49
N-Methylserotonin
1.13.11.52
Formyl-N-acetyl-5-methoxykynurenamine
5-Hydroxy-N-formylkynurenine
5-Hydroxy-L-tryptophan
6-Hydroxy-kynurenate
2.6.1.-
3.5.1.9
2.3.1.87
2.1.1.4
1.14.14.1
6-Hydroxymelatonin
4-(2-Amino-5-hydroxyphenyl)-2,4-dioxobutanoate
5-Hydroxy-kynurenine
Serotonin
N-Acetylserotonin
Melatonin
1.14.16.4
1.14.-.-
2.1.1.47
1.1.1.110
(R)-(indol-3-yl)-lactate
1.4.3.2
3-Methyl-indolepyruvate
4.1.1.74
Indole-3-acetaldehyde
1.1.1.190
3-Indoleglycol-aldehyde
2.6.1.27
2.6.1.99
Indolepyruvate
4.1.1.43
1.1.1.191
Indole-3-ethanol
1.13.99.3
2.6.1.-
N-Methyl-tryptamine
1.4.3.22
2.1.1.49
1.4.3.4
N-Acetylisatin
1.7.3.2
Acetylindoxyl
Indoxyl
1.14.16.-
Indole
4.1.99.1
Tryptophan
Tryptamine
4.1.1.105
1.14.13.-
N-Hydroxy-tryptamine
2.5.1.-
Indole-3-acetaldoxime
1.7.3.-
1.14.13.168
1.13.11.17
1.14.14.156
1.2.1.3
1.2.3.7
2-Formylamino-benzaldehyde
Indole-3-thiohydroximate
4.4.1.-
1.14.14.45
S-(indolylaceto-hydroximoyl)-L-cysteine
2.4.1.195
4.99.1.6
5-(2'-Carboxyethyl)-4,6-dihydroxypicolinate
5-(2'-Formylethyl)-4,6-dihydroxypicolinate
Indole-3-acetonitrile
1.2.1.-
Tryptophan biosynthesis
Indolylmethyldesulfo-glucosinolate
2.8.2.24
Glucobrassicin
3.2.1.147
3.5.5.1
Indoleacetate
5-(3'-Carboxy-3'-oxopropenyl)-4,6-dihydroxypicolinate
1.3.1.-
5-(3'-Carboxy-3'-oxopropyl)-4,6-dihydroxypicolinate
4.2.1.84
4.1.1.115
1.13.12.3
3.5.1.4
3-Methyl-dioxyindole
1.13.11.11
1.13.11.52
Indole-3-acetamide
3.5.1.-
1.14.11.-
Skatole
1.13.11.10
N-Formyl-kynurenine
3.7.1.3
Formyl-anthranilate
Tryptophan biosynthesis
Indole
2.5.1.-
1.13.11.-
2-Oxoindole-3-acetate
3.5.1.9
3.5.1.9
Aminobenzoate degradation
Kynurenate
L-Kynurenine
Anthranilate
2-Formamino-benzoylacetate
7,8-Dihydroxy-kynurenate
1.3.1.18
7,8-Dihydro-7,8-dihydroxykynurenate
1.14.99.2
2.6.1.7
4-(2-Aminophenyl)-2,4-dioxobutanoate
1.13.11.23
2,3-Dihydroxyindole
1.14.13.9
1.14.14.8
1.11.1.6
1.11.1.21
Cinnavalininate
3-Hydroxy-L-kynurenine
Xanthurenate
8-Methoxy-kynurenate
2.1.1.-
2.6.1.7
4-(2-Amino-3-hydroxyphenyl)-2,4-dioxobutanoate
3-Hydroxy-anthranilate
4.1.1.-
2-Amino-phenol
1.10.3.4
Isophenoxazine
4.1.1.-
2.1.1.-
3-Methoxy-anthranilate
1.13.11.6
4,8-Dihydroxy-quinoline
1.4.3.4
3-Hydroxy-kynurenamine
2-Amino-3-carboxymuconate semialdehyde
Quinolinate
Nicotinamide metabolism
4.1.1.45
Glycolysis
2-Aminomuconate semialdehyde
Picolinate
1.2.1.32
Acetoacetyl-CoA
Crotonoyl-CoA
Acetyl-CoA
2.3.1.9
1.1.1.35
4.2.1.17
1.3.8.6
OADH
1.5.1.-
3.5.99.5
Benzoate degradation
(S)-3-Hydroxy-butanoyl-CoA
Glutaryl-CoA
2-Oxoadipate
2-Amino-muconate
γ-Oxalo-crotonate
00380 11/6/20
(c) Kanehisa Laboratories

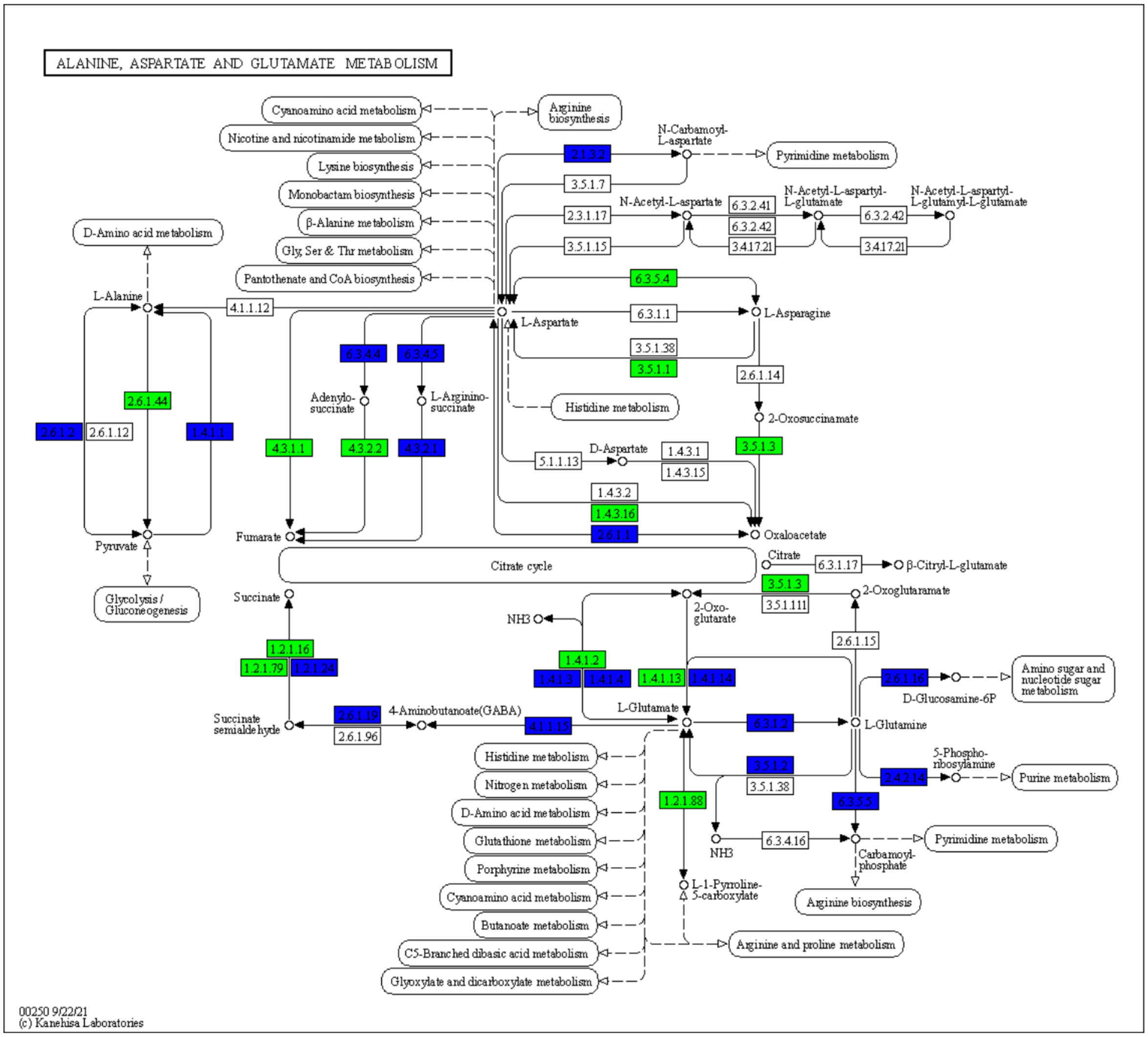

ALANINE, ASPARTATE AND GLUTAMATE METABOLISM
Cyanoamino acid metabolism
Nicotine and nicotinamide metabolism
Lysine biosynthesis
Monobactam biosynthesis
β-Alanine metabolism
Gly, Ser & Thr metabolism
Pantothenate and CoA biosynthesis
Arginine biosynthesis
N-Carbamoyl-L-aspartate
Pyrimidine metabolism
3.5.1.7
2.3.1.17
3.5.1.15
N-Acetyl-L-aspartate
6.3.2.41
6.3.2.42
3.4.17.21
N-Acetyl-L-aspartyl-L-glutamate
6.3.2.42
3.4.17.21
N-Acetyl-L-aspartyl-L-glutamyl-L-glutamate
D-Amino acid metabolism
L-Alanine
4.1.1.12
6.3.5.4
6.3.1.1
L-Aspartate
L-Asparagine
3.5.1.38
3.5.1.1
2.6.1.14
Histidine metabolism
2-Oxosuccinamate
2.6.1.44
2.6.1.12
Adenylo-succinate
L-Arginino-succinate
4.3.1.1
4.3.2.2
3.5.1.3
5.1.1.13
D-Aspartate
1.4.3.1
1.4.3.15
1.4.3.2
1.4.3.16
Pyruvate
Fumarate
Oxaloacetate
Citrate cycle
Citrate
6.3.1.17
β-Citryl-L-glutamate
Glycolysis / Gluconeogenesis
Succinate
3.5.1.3
3.5.1.111
2-Oxoglutaramate
2-Oxo-glutarate
NH3
2.6.1.15
1.2.1.16
1.2.1.79
1.4.1.2
1.4.1.13
Amino sugar and nucleotide sugar metabolism
D-Glucosamine-6P
Succinate semialdehyde
4-Aminobutanoate(GABA)
2.6.1.96
L-Glutamate
L-Glutamine
5-Phospho-ribosylamine
Purine metabolism
Histidine metabolism
Nitrogen metabolism
D-Amino acid metabolism
Glutathione metabolism
Porphyrine metabolism
Cyanoamino acid metabolism
Butanoate metabolism
C5-Branched dibasic acid metabolism
Glyoxylate and dicarboxylate metabolism
3.5.1.38
1.2.1.88
NH3
6.3.4.16
Carbamoyl-phosphate
Pyrimidine metabolism
Arginine biosynthesis
L-1-Pyrroline-5-carboxylate
Arginine and proline metabolism
00250 9/22/21
(c) Kanehisa Laboratories

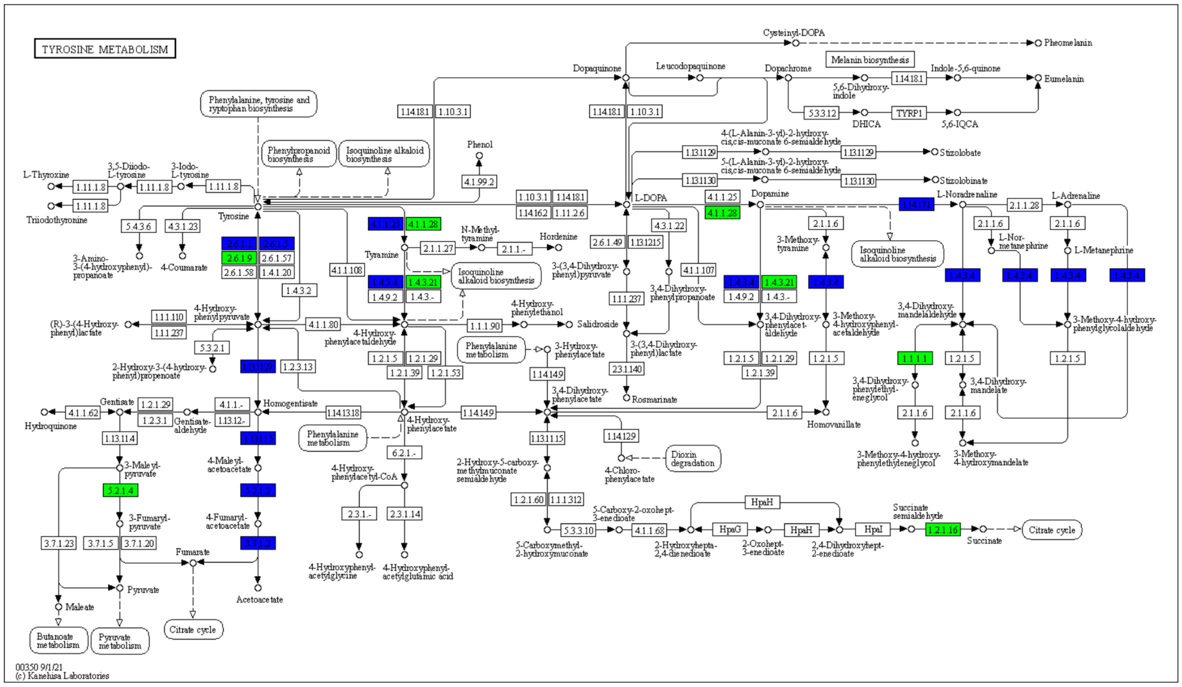
TYROSINE METABOLISM
Cysteinyl-DOPA
Pheomelanin
Dopaquinone
Leucodopaquinone
Dopachrome
Melanin biosynthesis
Indole-5,6-quinone
Eumelanin
5,6-Dihydroxy-indole
DHICA
5,6-IQCA
TYRP1
Phenylalanine, tyrosine and tryptophan biosynthesis
Phenylpropanoid biosynthesis
Isoquinoline alkaloid biosynthesis
Phenol
4-(L-Alanin-3-yl)-2-hydroxy-cis,cis-muconate 6-semialdehyde
5-(L-Alanin-3-yl)-2-hydroxy-cis,cis-muconate 6-semialdehyde
Stizolobate
Stizolobinate
L-Thyroxine
3,5-Diiodo-L-tyrosine
3-Iodo-L-tyrosine
Triiodothyronine
Tyrosine
L-DOPA
Dopamine
L-Noradrenaline
L-Adrenaline
L-Nor-metanephrine
L-Metanephrine
N-Methyl-tyramine
Hordenine
Tyramine
3-Methoxy-tyramine
3-Amino-3-(4-hydroxyphenyl)-propanoate
4-Coumarate
3-(3,4-Dihydroxy-phenyl)pyruvate
3,4-Dihydroxy-phenylpropanoate
4-Hydroxy-phenylpyruvate
(R)-3-(4-Hydroxy-phenyl)lactate
4-Hydroxy-phenylacetaldehyde
4-Hydroxy-phenylethanol
Salidroside
3,4-Dihydroxy-phenylacetaldehyde
3-Methoxy-4-hydroxyphenyl-acetaldehyde
3,4-Dihydroxy-mandelaldehyde
3-Methoxy-4-hydroxy-phenylglycolaldehyde
Phenylalanine metabolism
3-Hydroxy-phenylacetate
3-(3,4-Dihydroxy-phenyl)lactate
2-Hydroxy-3-(4-hydroxy-phenyl)propenoate
3,4-Dihydroxy-phenylethyleneglycol
3,4-Dihydroxy-mandelate
Gentisate
Homogentisate
Hydroquinone
Gentisate-aldehyde
3,4-Dihydroxy-phenylacetate
Rosmarinate
Homovanillate
4-Hydroxy-phenylacetate
3-Maleyl-pyruvate
4-Maleyl-acetoacetate
4-Hydroxy-phenylacetyl-CoA
2-Hydroxy-5-carboxy-methylmuconate semialdehyde
4-Chloro-phenylacetate
Dioxin degradation
3-Methoxy-4-hydroxy-phenylethyleneglycol
3-Methoxy-4-hydroxymandelate
3-Fumaryl-pyruvate
4-Fumaryl-acetoacetate
5-Carboxy-2-oxohept-3-enedioate
Succinate semialdehyde
HpaH
HpaG
HpaI
Succinate
Citrate cycle
Fumarate
Pyruvate
Acetoacetate
4-Hydroxyphenyl-acetylglycine
4-Hydroxyphenyl-acetylglutamic acid
5-Carboxymethyl-2-hydroxymuconate
2-Hydroxyhepta-2,4-dienedioate
2-Oxohept-3-enedioate
2,4-Dihydroxyhept-2-enedioate
Maleate
Butanoate metabolism
Pyruvate metabolism
00350 9/1/21
(c) Kanehisa Laboratories

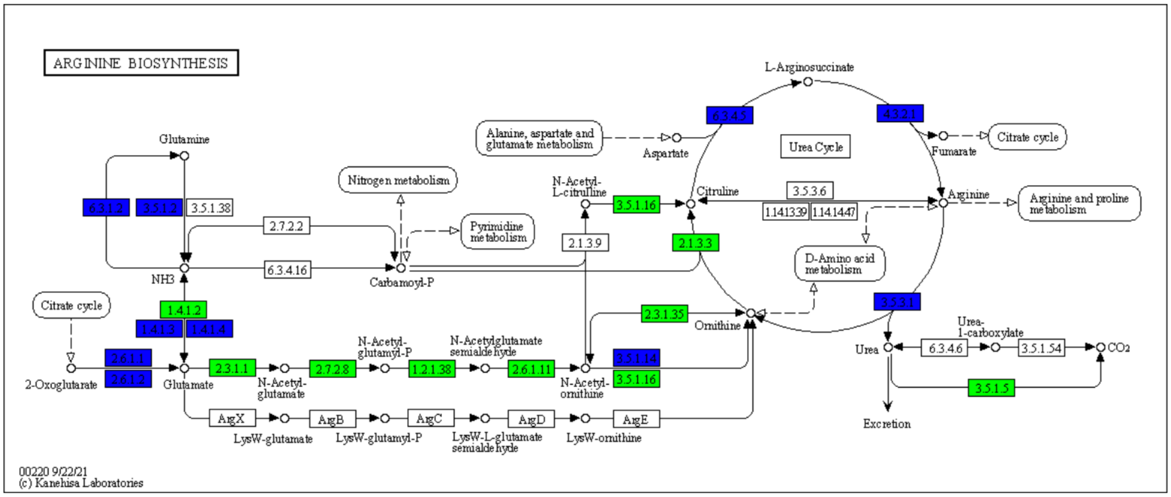
ARGININE BIOSYNTHESIS
L-Arginosuccinate
6.3.4.5
4.3.2.1
Alanine, aspartate and glutamate metabolism
Aspartate
Urea Cycle
Fumarate
Citrate cycle
Glutamine
Nitrogen metabolism
N-Acetyl-L-citrulline
Citruline
3.5.3.6
Arginine
Arginine and proline metabolism
6.3.1.2
3.5.1.2
3.5.1.38
2.7.2.2
Pyrimidine metabolism
3.5.1.16
1.14.13.39
1.14.14.47
2.1.3.9
2.1.3.3
D-Amino acid metabolism
NH3
6.3.4.16
Carbamoyl-P
Citrate cycle
1.4.1.2
1.4.1.3
1.4.1.4
2.3.1.35
Ornithine
3.5.3.1
Urea
Urea-1-carboxylate
6.3.4.6
3.5.1.54
CO2
2-Oxoglutarate
2.6.1.1
2.6.1.2
Glutamate
2.3.1.1
N-Acetyl-glutamate
2.7.2.8
N-Acetyl-glutamyl-P
1.2.1.38
N-Acetylglutamate semialdehyde
2.6.1.11
N-Acetyl-ornithine
3.5.1.14
3.5.1.16
3.5.1.5
Excretion
ArgX
ArgB
ArgC
ArgD
ArgE
LysW-glutamate
LysW-glutamyl-P
LysW-L-glutamate semialdehyde
LysW-ornithine
00220 9/22/21
(c) Kanehisa Laboratories

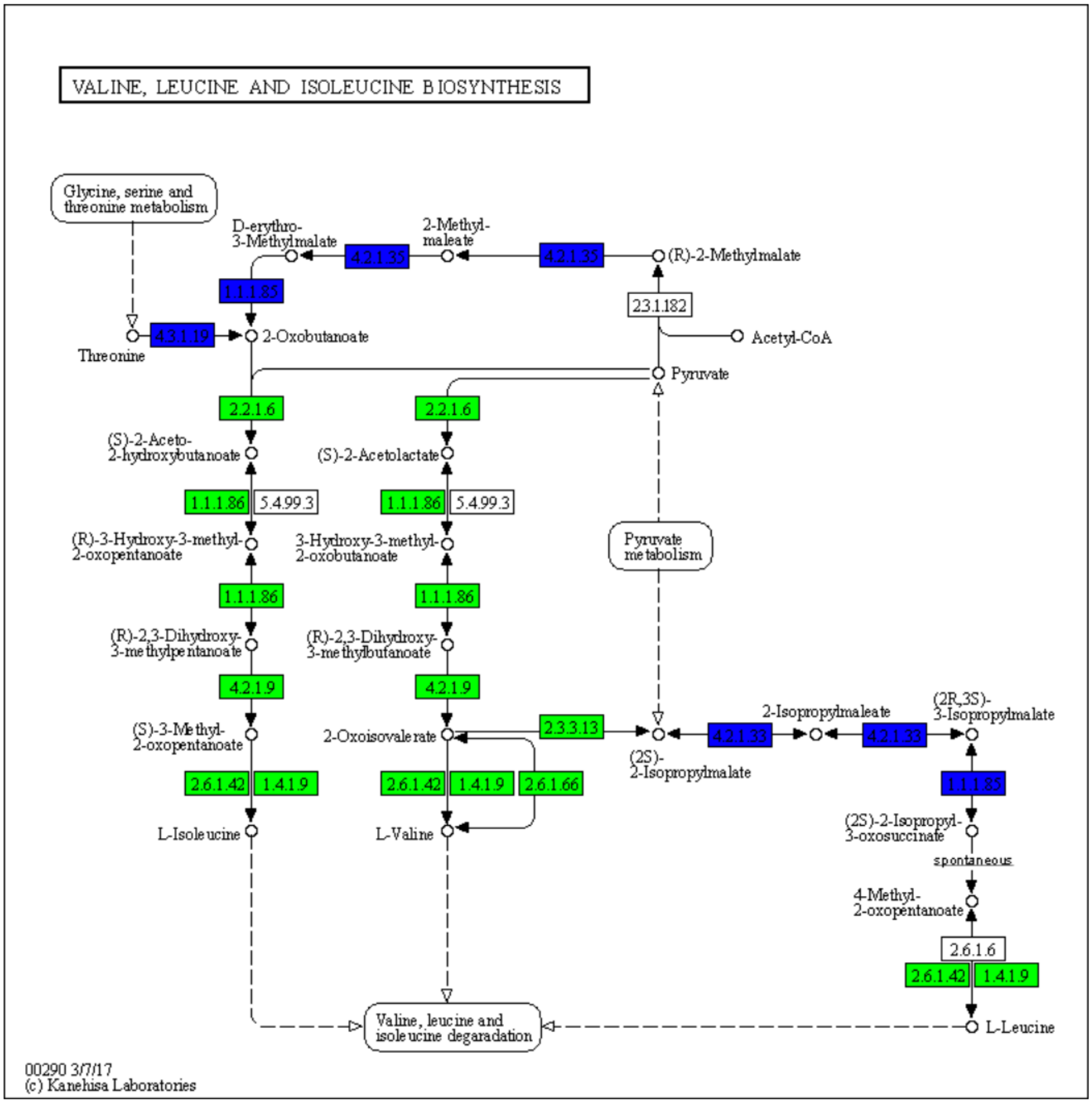
VALINE, LEUCINE AND ISOLEUCINE BIOSYNTHESIS
Glycine, serine and threonine metabolism
D-erythro-3-Methylmalate
2-Methyl-maleate
4.2.1.35
4.2.1.35
(R)-2-Methylmalate
1.1.1.85
2.3.1.182
Threonine
4.3.1.19
2-Oxobutanoate
Acetyl-CoA
Pyruvate
2.2.1.6
2.2.1.6
(S)-2-Aceto-2-hydroxybutanoate
(S)-2-Acetolactate
1.1.1.86
5.4.99.3
1.1.1.86
5.4.99.3
(R)-3-Hydroxy-3-methyl-2-oxopentanoate
3-Hydroxy-3-methyl-2-oxobutanoate
Pyruvate metabolism
1.1.1.86
1.1.1.86
(R)-2,3-Dihydroxy-3-methylpentanoate
(R)-2,3-Dihydroxy-3-methylbutanoate
4.2.1.9
4.2.1.9
(S)-3-Methyl-2-oxopentanoate
2-Oxoisovalerate
2.3.3.13
2-Isopropylmaleate
(2R,3S)-3-Isopropylmalate
4.2.1.33
4.2.1.33
(2S)-2-Isopropylmalate
2.6.1.42
1.4.1.9
2.6.1.42
1.4.1.9
2.6.1.66
1.1.1.85
L-Isoleucine
L-Valine
(2S)-2-Isopropyl-3-oxosuccinate
spontaneous
4-Methyl-2-oxopentanoate
2.6.1.6
2.6.1.42
1.4.1.9
Valine, leucine and isoleucine degaradation
L-Leucine
00290 3/7/17
(c) Kanehisa Laboratories

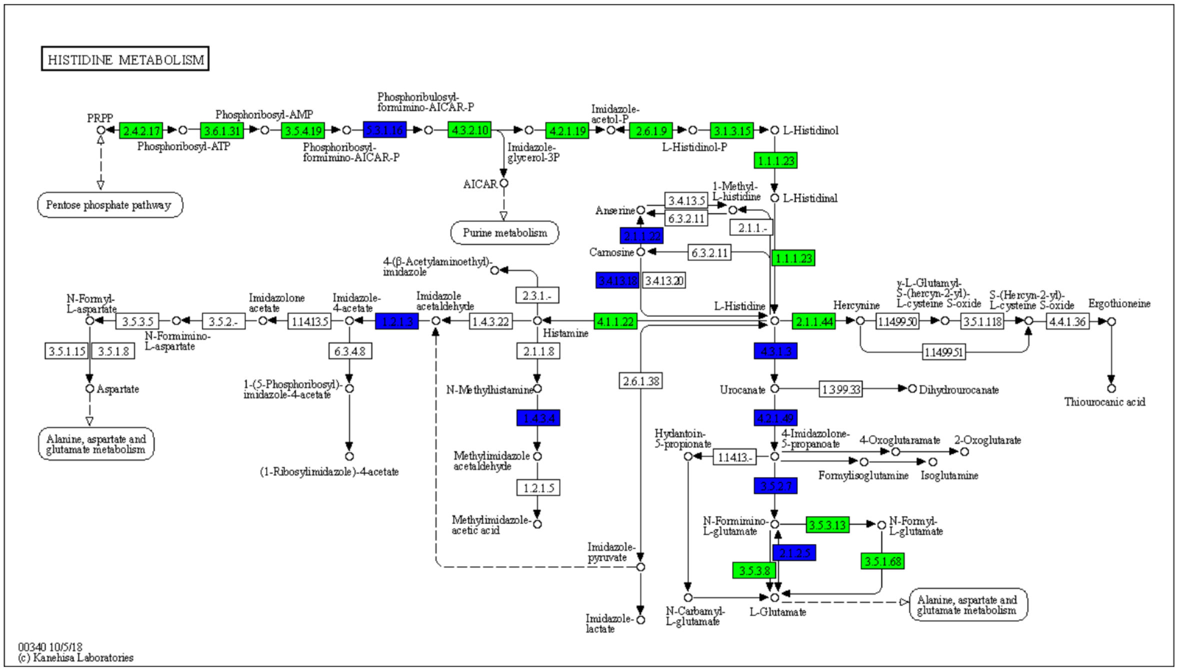

HISTIDINE METABOLISM
PRPP
2.4.2.17
Phosphoribosyl-ATP
3.6.1.31
Phosphoribosyl-AMP
3.5.4.19
Phosphoribosyl-formimino-AICAR-P
Phosphoribulosyl-formimino-AICAR-P
4.3.2.10
Imidazole-glycerol-3P
AICAR
Purine metabolism
4.2.1.19
Imidazole-acetol-P
2.6.1.9
L-Histidinol-P
3.1.3.15
L-Histidinol
1.1.1.23
L-Histidinal
Pentose phosphate pathway
1-Methyl-L-histidine
3.4.13.5
6.3.2.11
2.1.1.-
Anserine
Carnosine
6.3.2.11
3.4.13.20
1.1.1.23
4-(β-Acetylaminoethyl)-imidazole
2.3.1.-
N-Formyl-L-aspartate
3.5.3.5
3.5.2.-
Imidazolone acetate
1.14.13.5
Imidazole-4-acetate
Imidazole acetaldehyde
1.4.3.22
Histamine
4.1.1.22
L-Histidine
2.1.1.44
Hercynine
1.14.99.50
γ-L-Glutamyl-S-(hercyn-2-yl)-L-cysteine S-oxide
3.5.1.118
S-(Hercyn-2-yl)-L-cysteine S-oxide
4.4.1.36
Ergothioneine
1.14.99.51
Thiourocanic acid
N-Formimino-L-aspartate
3.5.1.15
3.5.1.8
Aspartate
6.3.4.8
1-(5-Phosphoribosyl)-imidazole-4-acetate
(1-Ribosylimidazole)-4-acetate
Alanine, aspartate and glutamate metabolism
2.1.1.8
N-Methylhistamine
Methylimidazole acetaldehyde
1.2.1.5
Methylimidazole-acetic acid
2.6.1.38
Urocanate
1.3.99.33
Dihydrourocanate
Hydantoin-5-propionate
1.14.13.-
4-Imidazolone-5-propanoate
4-Oxoglutaramate
2-Oxoglutarate
Formylisoglutamine
Isoglutamine
N-Formimino-L-glutamate
3.5.3.13
N-Formyl-L-glutamate
3.5.1.68
3.5.3.8
Imidazole-pyruvate
Imidazole-lactate
N-Carbamyl-L-glutamate
L-Glutamate
Alanine, aspartate and glutamate metabolism
00340 10/5/18
(c) Kanehisa Laboratories

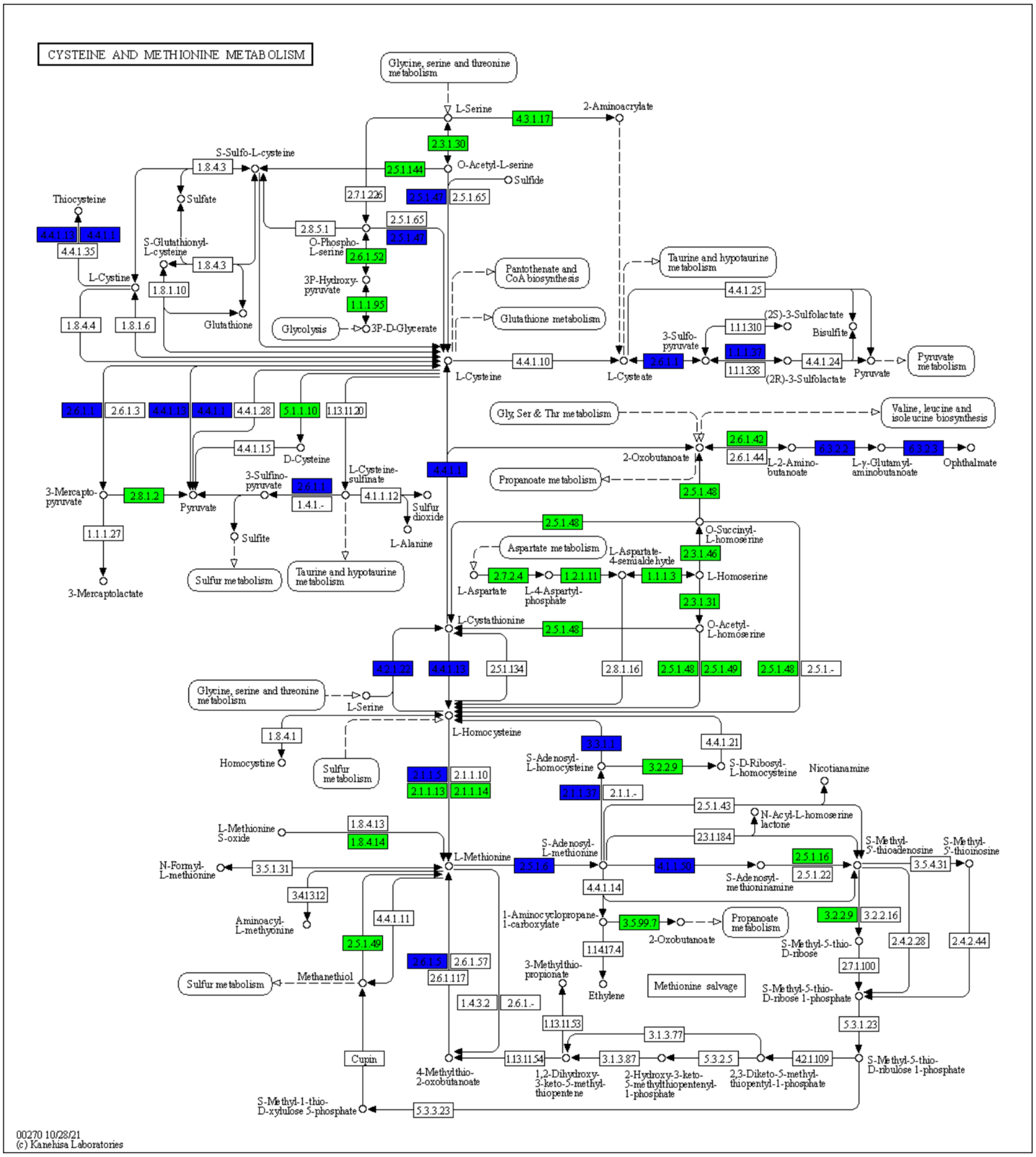

CYSTEINE AND METHIONINE METABOLISM
Glycine, serine and threonine metabolism
L-Serine
4.3.1.17
2-Aminoacrylate
2.3.1.30
O-Acetyl-L-serine
Sulfide
S-Sulfo-L-cysteine
1.8.4.3
2.5.1.144
2.7.1.226
2.5.1.65
Sulfate
Thiocysteine
4.4.1.35
S-Glutathionyl-L-cysteine
2.8.5.1
O-Phospho-L-serine
2.6.1.52
1.8.4.3
3P-Hydroxy-pyruvate
1.1.1.95
Pantothenate and CoA biosynthesis
Taurine and hypotaurine metabolism
L-Cystine
1.8.1.10
Glutathione metabolism
1.8.4.4
1.8.1.6
Glutathione
Glycolysis
3P-D-Glycerate
4.4.1.25
(2S)-3-Sulfolactate
1.1.1.310
3-Sulfo-pyruvate
Bisulfite
L-Cysteine
4.4.1.10
L-Cysteate
1.1.1.338
4.4.1.24
(2R)-3-Sulfolactate
Pyruvate
Pyruvate metabolism
2.6.1.3
4.4.1.28
1.1.10
1.13.11.20
Gly, Ser & Thr metabolism
Valine, leucine and isoleucine biosynthesis
4.4.1.15
D-Cysteine
2.6.1.42
2-Oxobutanoate
2.6.1.44
L-2-Amino-butanoate
L-γ-Glutamyl-aminobutanoate
Ophthalmate
3-Sulfino-pyruvate
L-Cysteine-sulfinate
Propanoate metabolism
3-Mercapto-pyruvate
2.8.1.2
Pyruvate
1.4.1.-
4.1.1.12
Sulfur dioxide
L-Alanine
2.5.1.48
1.1.1.27
Sulfite
O-Succinyl-L-homoserine
Aspartate metabolism
L-Aspartate-4-semialdehyde
2.3.1.46
Sulfur metabolism
Taurine and hypotaurine metabolism
L-Aspartate
2.7.2.4
L-4-Aspartyl-phosphate
1.2.1.11
1.1.1.3
L-Homoserine
3-Mercaptolactate
2.3.1.31
L-Cystathionine
2.5.1.48
O-Acetyl-L-homoserine
2.5.1.134
2.8.1.16
2.5.1.48
2.5.1.49
2.5.1.48
2.5.1.-
Glycine, serine and threonine metabolism
L-Serine
L-Homocysteine
1.8.4.1
Homocystine
Sulfur metabolism
4.4.1.21
S-Adenosyl-L-homocysteine
3.2.2.9
S-D-Ribosyl-L-homocysteine
2.1.1.10
2.1.1.13
2.1.1.14
2.1.1.-
Nicotianamine
2.5.1.43
N-Acyl-L-homoserine lactone
L-Methionine S-oxide
1.8.4.13
1.8.4.14
2.3.1.184
S-Methyl-5'-thioadenosine
S-Methyl-5'-thioinosine
L-Methionine
S-Adenosyl-L-methionine
2.5.1.16
3.5.4.31
N-Formyl-L-methionine
3.5.1.31
S-Adenosyl-methioninamine
2.5.1.22
3.4.13.12
4.4.1.14
Aminoacyl-L-methionine
4.4.1.11
1-Aminocyclopropane-1-carboxylate
3.5.99.7
2-Oxobutanoate
Propanoate metabolism
3.2.2.9
3.2.2.16
2.5.1.49
S-Methyl-5-thio-D-ribose
2.4.2.28
2.4.2.44
1.14.17.4
2.6.1.57
3-Methylthio-propionate
2.7.1.100
Sulfur metabolism
Methanethiol
2.6.1.117
Ethylene
Methionine salvage
S-Methyl-5-thio-D-ribose 1-phosphate
1.4.3.2
2.6.1.-
5.3.1.23
1.13.11.53
3.1.3.77
Cupin
1.13.11.54
3.1.3.87
5.3.2.5
4.2.1.109
4-Methylthio-2-oxobutanoate
1,2-Dihydroxy-3-keto-5-methyl-thiopentene
2-Hydroxy-3-keto-5-methylthiopentenyl-1-phosphate
2,3-Diketo-5-methyl-thiopentyl-1-phosphate
S-Methyl-5-thio-D-ribulose 1-phosphate
S-Methyl-1-thio-D-xylulose 5-phosphate
5.3.3.23
00270 10/28/21
(c) Kanehisa Laboratories

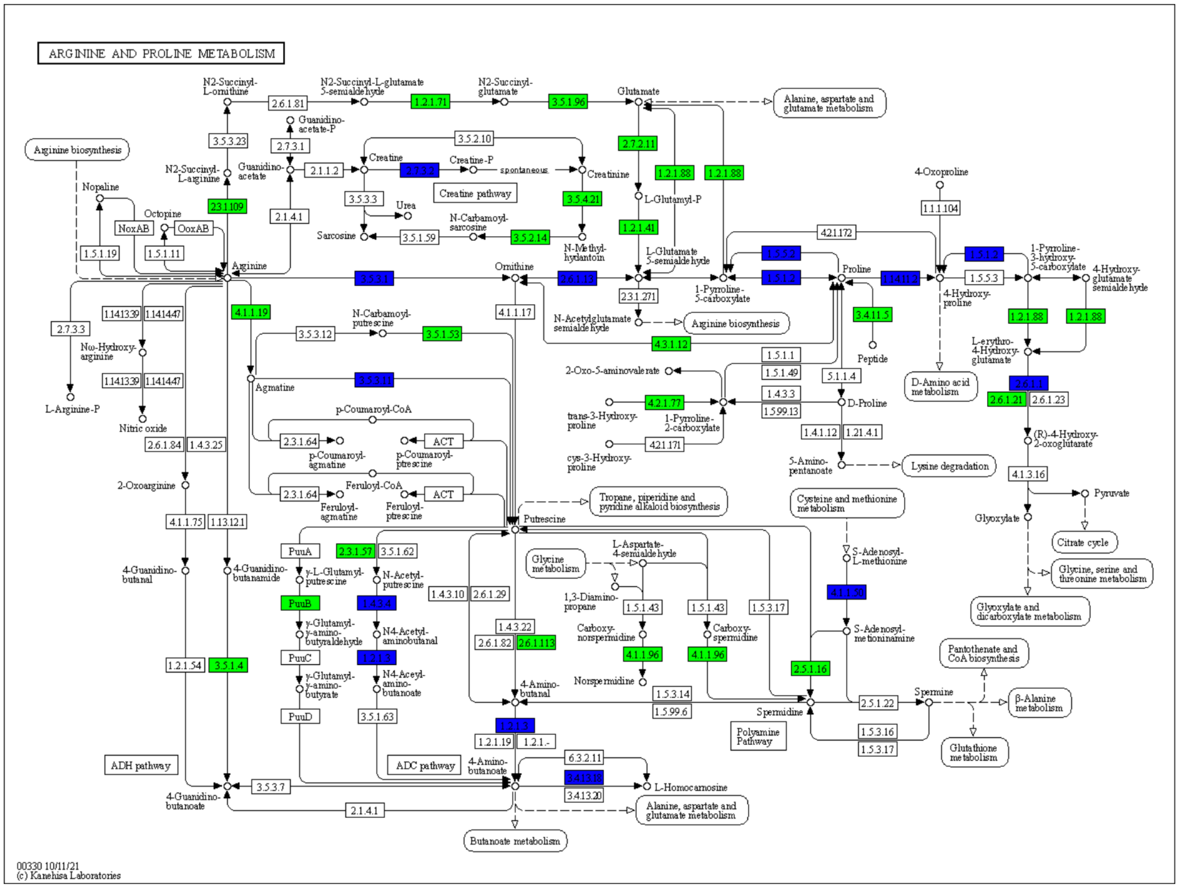

ARGININE AND PROLINE METABOLISM
N2-Succinyl-L-ornithine
2.6.1.81
N2-Succinyl-L-glutamate 5-semialdehyde
1.2.1.71
N2-Succinyl-glutamate
3.5.1.96
Glutamate
Alanine, aspartate and glutamate metabolism
Guanidino-acetate-P
3.5.3.23
2.7.3.1
3.5.2.10
Arginine biosynthesis
2.7.2.11
N2-Succinyl-L-arginine
Guanidino-acetate
2.1.1.2
Creatine
Creatine-P
spontaneous
Creatinine
1.2.1.88
1.2.1.88
4-Oxoproline
Nopaline
2.3.1.109
3.5.3.3
Urea
Creatine pathway
3.5.4.21
L-Glutamyl-P
1.1.1.104
Octopine
NoxAB
OoxAB
2.1.4.1
N-Carbamoyl-sarcosine
1.2.1.41
4.2.1.72
Sarcosine
3.5.1.59
3.5.2.14
N-Methyl-hydantoin
L-Glutamate 5-semialdehyde
1-Pyrroline-3-hydroxy-5-carboxylate
1.5.1.19
1.5.1.11
Arginine
Ornithine
Proline
4-Hydroxy-glutamate semialdehyde
1.5.5.3
1-Pyrroline-5-carboxylate
4-Hydroxy-proline
2.3.1.271
4.1.1.17
N-Acetylglutamate semialdehyde
Arginine biosynthesis
3.4.11.5
1.2.1.88
1.2.1.88
1.14.13.39
1.14.14.47
4.1.1.19
N-Carbamoyl-putrescine
2.7.3.3
3.5.3.12
3.5.1.53
4.3.1.12
Peptide
Nω-Hydroxy-arginine
L-erythro-4-Hydroxy-glutamate
1.5.1.1
D-Amino acid metabolism
1.5.1.49
2-Oxo-5-aminovalerate
5.1.1.4
1.14.13.39
1.14.14.47
Agmatine
1.4.3.3
D-Proline
2.6.1.21
2.6.1.23
L-Arginine-P
trans-3-Hydroxy-proline
4.2.1.77
1.5.99.13
p-Coumaroyl-CoA
1-Pyrroline-2-carboxylate
Nitric oxide
ACT
1.4.1.12
1.21.4.1
2.3.1.64
(R)-4-Hydroxy-2-oxoglutarate
2.6.1.84
1.4.3.25
p-Coumaroyl-agmatine
p-Coumaroyl-ptrescine
4.2.1.171
cys-3-Hydroxy-proline
5-Amino-pentanoate
Lysine degradation
4.1.3.16
Feruloyl-CoA
2-Oxoarginine
Pyruvate
ACT
2.3.1.64
Tropane, piperidine and pyridine alkaloid biosynthesis
Feruloyl-agmatine
Feruloyl-ptrescine
Cysteine and methionine metabolism
Glyoxylate
4.1.1.75
1.13.12.1
Putrescine
Citrate cycle
L-Aspartate-4-semialdehyde
PuuA
2.3.1.57
3.5.1.62
S-Adenosyl-L-methionine
Glycine metabolism
4-Guanidino-butanal
4-Guanidino-butanamide
γ-L-Glutamyl-putrescine
N-Acetyl-putrescine
Glycine, serine and threonine metabolism
1.4.3.10
2.6.1.29
1,3-Diamino-propane
1.5.1.43
1.5.1.43
1.5.3.17
PuuB
Glyoxylate and dicarboxylate metabolism
1.4.3.22
γ-Glutamyl-γ-amino-butyraldehyde
N4-Acetyl-aminobutanal
Carboxy-norspermidine
Carboxy-spermidine
S-Adenosyl-methioninamine
2.6.1.82
2.6.1.113
Pantothenate and CoA biosynthesis
1.2.1.54
3.5.1.4
PuuC
4.1.1.96
4.1.1.96
2.5.1.16
Norspermidine
γ-Glutamyl-γ-amino-butyrate
N4-Acetyl-amino-butanoate
4-Amino-butanal
1.5.3.14
Spermine
β-Alanine metabolism
Spermidine
2.5.1.22
1.5.99.6
PuuD
3.5.1.63
Polyamine Pathway
1.5.3.16
1.2.1.19
1.2.1.-
1.5.3.17
Glutathione metabolism
ADH pathway
ADC pathway
4-Amino-butanoate
6.3.2.11
4-Guanidino-butanoate
3.5.3.7
L-Homocarnosine
3.4.13.20
2.1.4.1
Alanine, aspartate and glutamate metabolism
Butanoate metabolism
00330 10/11/21
(c) Kanehisa Laboratories

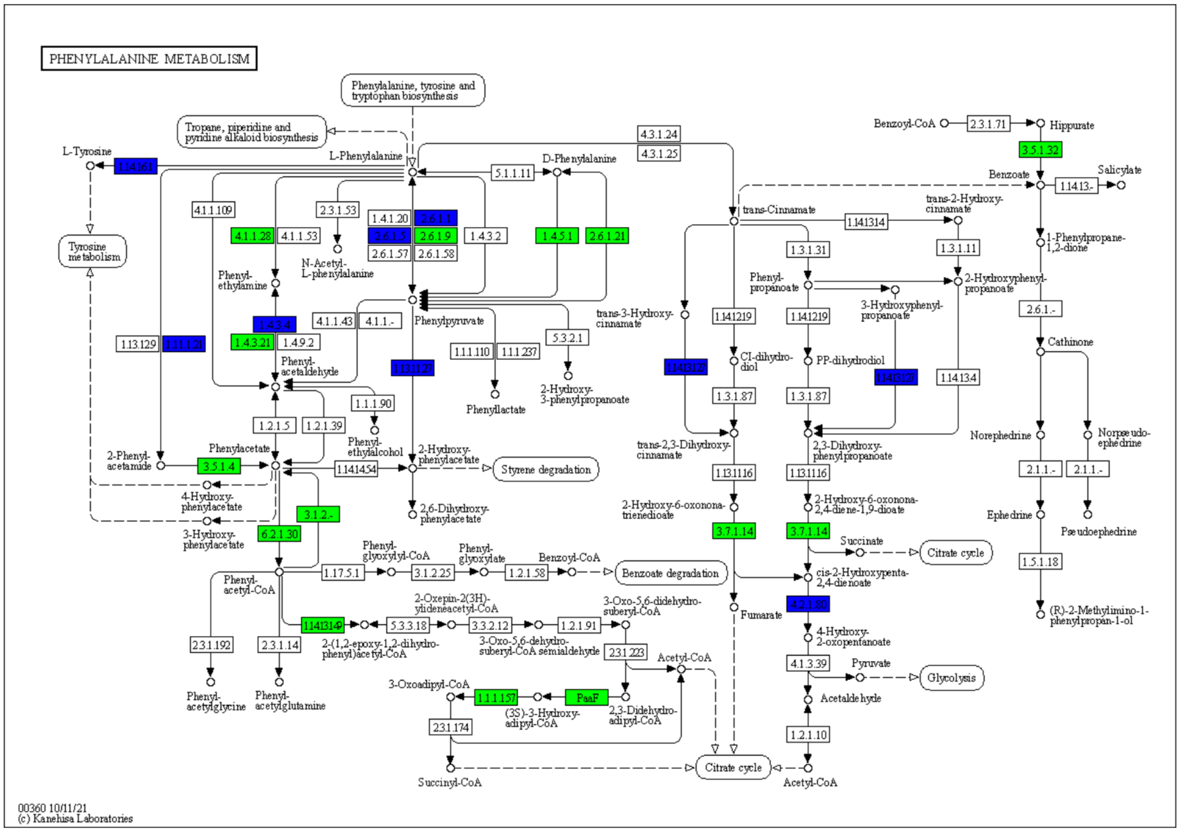
PHENYLALANINE METABOLISM
Phenylalanine, tyrosine and tryptophan biosynthesis
Tropane, piperidine and pyridine alkaloid biosynthesis
L-Tyrosine
L-Phenylalanine
D-Phenylalanine
Tyrosine metabolism
4.3.1.24
4.3.1.25
5.1.1.11
4.1.1.109
4.1.1.28
4.1.1.53
2.3.1.53
1.4.1.20
2.6.1.57
2.6.1.58
2.6.1.9
1.4.3.2
1.4.5.1
2.6.1.21
N-Acetyl-L-phenylalanine
Phenyl-ethylamine
Phenylpyruvate
4.1.1.43
4.1.1.-
1.4.3.21
1.4.9.2
1.13.12.9
1.1.1.110
1.1.1.237
5.3.2.1
Phenyl-acetaldehyde
Phenyllactate
2-Hydroxy-3-phenylpropanoate
1.1.1.90
1.2.1.5
1.2.1.39
Phenylacetate
Phenyl-ethylalcohol
2-Phenyl-acetamide
3.5.1.4
1.14.14.54
2-Hydroxy-phenylacetate
Styrene degradation
4-Hydroxy-phenylacetate
3-Hydroxy-phenylacetate
3.1.2.-
6.2.1.30
2,6-Dihydroxy-phenylacetate
Phenyl-acetyl-CoA
1.17.5.1
Phenyl-glyoxylyl-CoA
3.1.2.25
Phenyl-glyoxylate
1.2.1.58
Benzoyl-CoA
Benzoate degradation
2-Oxepin-2(3H)-ylideneacetyl-CoA
5.3.3.18
3.3.2.12
1.2.1.91
3-Oxo-5,6-didehydro-suberyl-CoA
2-(1,2-epoxy-1,2-dihydro-phenyl)acetyl-CoA
3-Oxo-5,6-dehydro-suberyl-CoA semialdehyde
2.3.1.223
Acetyl-CoA
2.3.1.192
2.3.1.14
Phenyl-acetylglycine
Phenyl-acetylglutamine
3-Oxoadipyl-CoA
1.1.1.157
PaaF
(3S)-3-Hydroxy-adipyl-CoA
2,3-Didehydro-adipyl-CoA
2.3.1.174
Succinyl-CoA
Citrate cycle
Acetyl-CoA
trans-Cinnamate
1.14.13.14
trans-2-Hydroxy-cinnamate
1.3.1.31
1.3.1.11
Phenyl-propanoate
3-Hydroxyphenyl-propanoate
2-Hydroxyphenyl-propanoate
trans-3-Hydroxy-cinnamate
1.14.12.19
Cl-dihydro-diol
PP-dihydrodiol
1.14.13.4
1.3.1.87
trans-2,3-Dihydroxy-cinnamate
2,3-Dihydroxy-phenylpropanoate
1.13.11.16
2-Hydroxy-6-oxonona-trienedioate
2-Hydroxy-6-oxonona-2,4-diene-1,9-dioate
3.7.1.14
Succinate
Citrate cycle
cis-2-Hydroxypenta-2,4-dienoate
Fumarate
4-Hydroxy-2-oxopentanoate
4.1.3.39
Pyruvate
Glycolysis
Acetaldehyde
1.2.1.10
Acetyl-CoA
Benzoyl-CoA
2.3.1.71
Hippurate
3.5.1.32
Benzoate
Salicylate
1.14.13.-
1-Phenylpropane-1,2-dione
2.6.1.-
Cathinone
Norephedrine
Norpseudo-ephedrine
2.1.1.-
Ephedrine
Pseudoephedrine
1.5.1.18
(R)-2-Methylimino-1-phenylpropan-1-ol
00360 10/11/21
(c) Kanehisa Laboratories

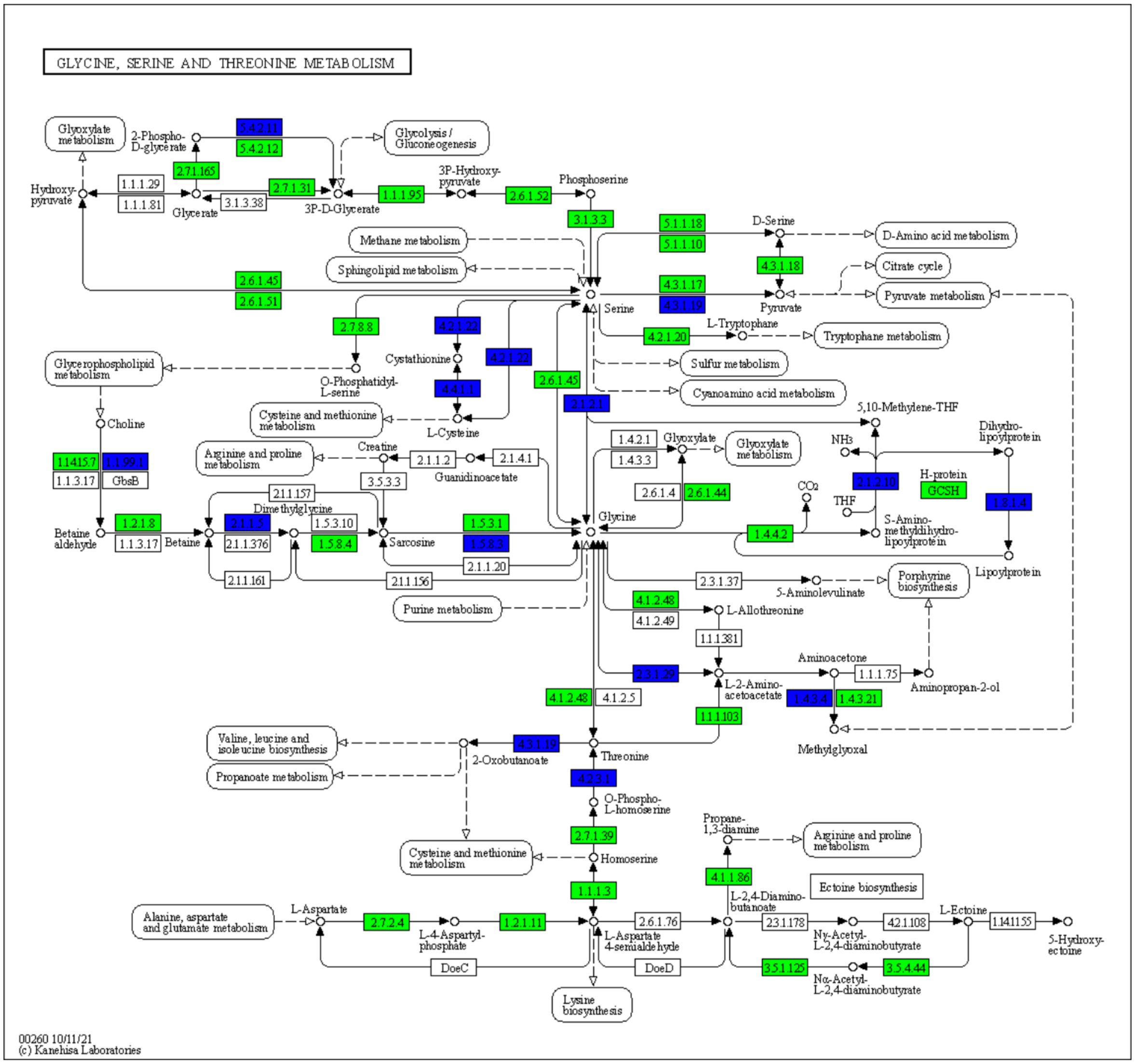
GLYCINE, SERINE AND THREONINE METABOLISM
Glyoxylate metabolism
2-Phospho-D-glycerate
5.4.2.12
Glycolysis / Gluconeogenesis
2.7.1.165
1.1.1.29
1.1.1.81
Hydroxy-pyruvate
Glycerate
3.1.3.38
2.7.1.31
3P-D-Glycerate
1.1.1.95
3P-Hydroxy-pyruvate
2.6.1.52
Phosphoserine
3.1.3.3
Methane metabolism
Sphingolipid metabolism
5.1.1.18
5.1.1.10
D-Serine
D-Amino acid metabolism
4.3.1.18
Citrate cycle
2.6.1.45
2.6.1.51
4.3.1.17
Pyruvate
Pyruvate metabolism
Serine
2.7.8.8
Cystathionine
4.2.1.20
L-Tryptophane
Tryptophane metabolism
Glycerophospholipid metabolism
O-Phosphatidyl-L-serine
2.6.1.45
Sulfur metabolism
Cyanoamino acid metabolism
Choline
Cysteine and methionine metabolism
L-Cysteine
5,10-Methylene-THF
Arginine and proline metabolism
Creatine
2.1.1.2
2.1.4.1
Guanidinoacetate
1.4.2.1
1.4.3.3
Glyoxylate
Glyoxylate metabolism
NH3
Dihydro-lipoylprotein
1.1.99.1
1.1.3.17
GbsB
3.5.3.3
2.1.1.157
Dimethylglycine
2.6.1.4
2.6.1.44
H-protein
GCSH
CO2
THF
1.2.1.8
1.1.3.17
Betaine aldehyde
Betaine
2.1.1.376
1.5.3.10
1.5.8.4
1.5.3.1
Sarcosine
Glycine
1.4.4.2
S-Amino-methyldihydro-lipoylprotein
2.1.1.20
Lipoylprotein
2.1.1.161
2.1.1.156
2.3.1.37
5-Aminolevulinate
Porphyrine biosynthesis
Purine metabolism
4.1.2.48
4.1.2.49
L-Allothreonine
1.1.1.381
Aminoacetone
1.1.1.75
L-2-Amino-acetoacetate
Aminopropan-2-ol
4.1.2.48
4.1.2.5
1.4.3.21
1.1.1.103
Methylglyoxal
Valine, leucine and isoleucine biosynthesis
2-Oxobutanoate
Threonine
Propanoate metabolism
O-Phospho-L-homoserine
2.7.1.39
Propane-1,3-diamine
Arginine and proline metabolism
Cysteine and methionine metabolism
Homoserine
4.1.1.86
Ectoine biosynthesis
1.1.1.3
L-2,4-Diamino-butanoate
Alanine, aspartate and glutamate metabolism
L-Aspartate
2.7.2.4
1.2.1.11
L-4-Aspartyl-phosphate
L-Aspartate 4-semialdehyde
2.6.1.76
2.3.1.178
Nγ-Acetyl-L-2,4-diaminobutyrate
4.2.1.108
L-Ectoine
1.14.11.55
5-Hydroxy-ectoine
DoeC
DoeD
3.5.1.125
3.5.4.44
Nα-Acetyl-L-2,4-diaminobutyrate
Lysine biosynthesis
00260 10/11/21
(c) Kanehisa Laboratories

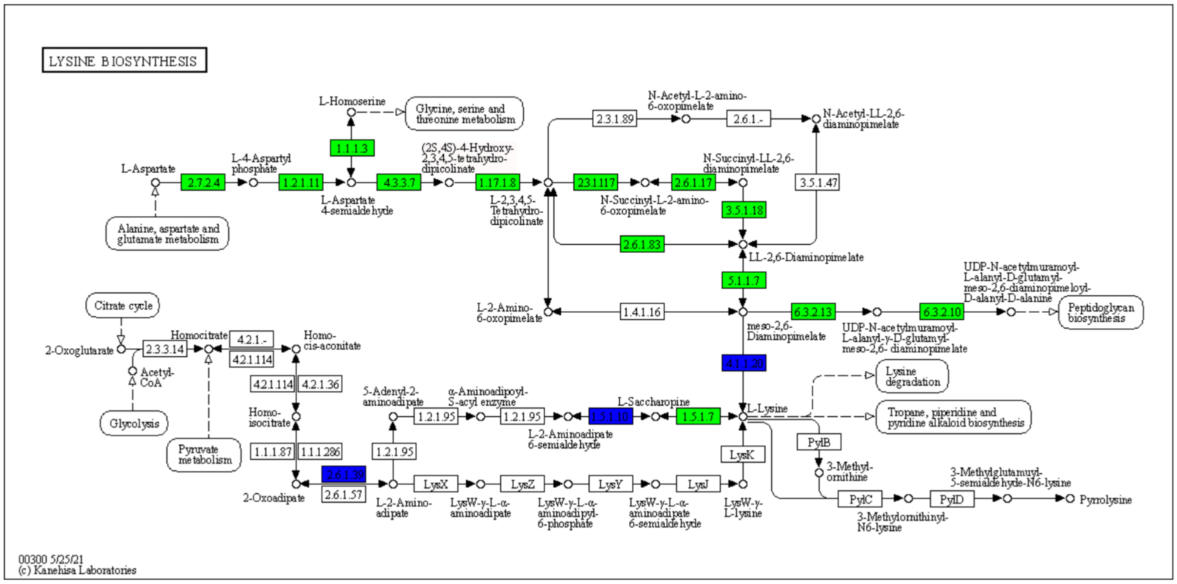
LYSINE BIOSYNTHESIS
L-Homoserine
Glycine, serine and threonine metabolism
1.1.1.3
N-Acetyl-L-2-amino-6-oxopimelate
2.3.1.89
2.6.1.-
N-Acetyl-LL-2,6-diaminopimelate
L-Aspartate
L-4-Aspartyl phosphate
2.7.2.4
1.2.1.11
4.3.3.7
(2S,4S)-4-Hydroxy-2,3,4,5-tetrahydro-dipicolinate
1.17.1.8
2.3.1.117
2.6.1.17
N-Succinyl-LL-2,6-diaminopimelate
3.5.1.47
L-Aspartate 4-semialdehyde
L-2,3,4,5-Tetrahydro-dipicolinate
N-Succinyl-L-2-amino-6-oxopimelate
3.5.1.18
Alanine, aspartate and glutamate metabolism
2.6.1.83
LL-2,6-Diaminopimelate
5.1.1.7
UDP-N-acetylmuramoyl-L-alanyl-D-glutamyl-meso-2,6-diaminopimeloyl-D-alanyl-D-alanine
Citrate cycle
L-2-Amino-6-oxopimelate
1.4.1.16
6.3.2.13
6.3.2.10
Peptidoglycan biosynthesis
meso-2,6-Diaminopimelate
UDP-N-acetylmuramoyl-L-alanyl-γ-D-glutamyl-meso-2,6-diaminopimelate
Homocitrate
4.2.1.-
Homo-cis-aconitate
2-Oxoglutarate
2.3.3.14
4.2.1.114
Acetyl-CoA
Lysine degradation
4.2.1.114
4.2.1.36
5-Adenyl-2-aminoadipate
α-Aminoadipoyl-S-acyl enzyme
L-Saccharopine
Glycolysis
Homo-isocitrate
1.2.1.95
1.2.1.95
1.5.1.7
L-Lysine
Tropane, piperidine and pyridine alkaloid biosynthesis
L-2-Aminoadipate 6-semialdehyde
Pyruvate metabolism
1.1.1.87
1.1.1.286
1.2.1.95
LysK
PylB
3-Methyl-ornithine
3-Methylglutamyl-5-semialdehyde-N6-lysine
2-Oxoadipate
2.6.1.57
L-2-Amino-adipate
LysX
LysW-γ-L-α-aminoadipate
LysZ
LysW-γ-L-α-aminoadipyl-6-phosphate
LysY
LysW-γ-L-α-aminoadipate 6-semialdehyde
LysJ
LysW-γ-L-lysine
PylC
PylD
Pyrrolysine
3-Methylornithinyl-N6-lysine
00300 5/25/21
(c) Kanehisa Laboratories

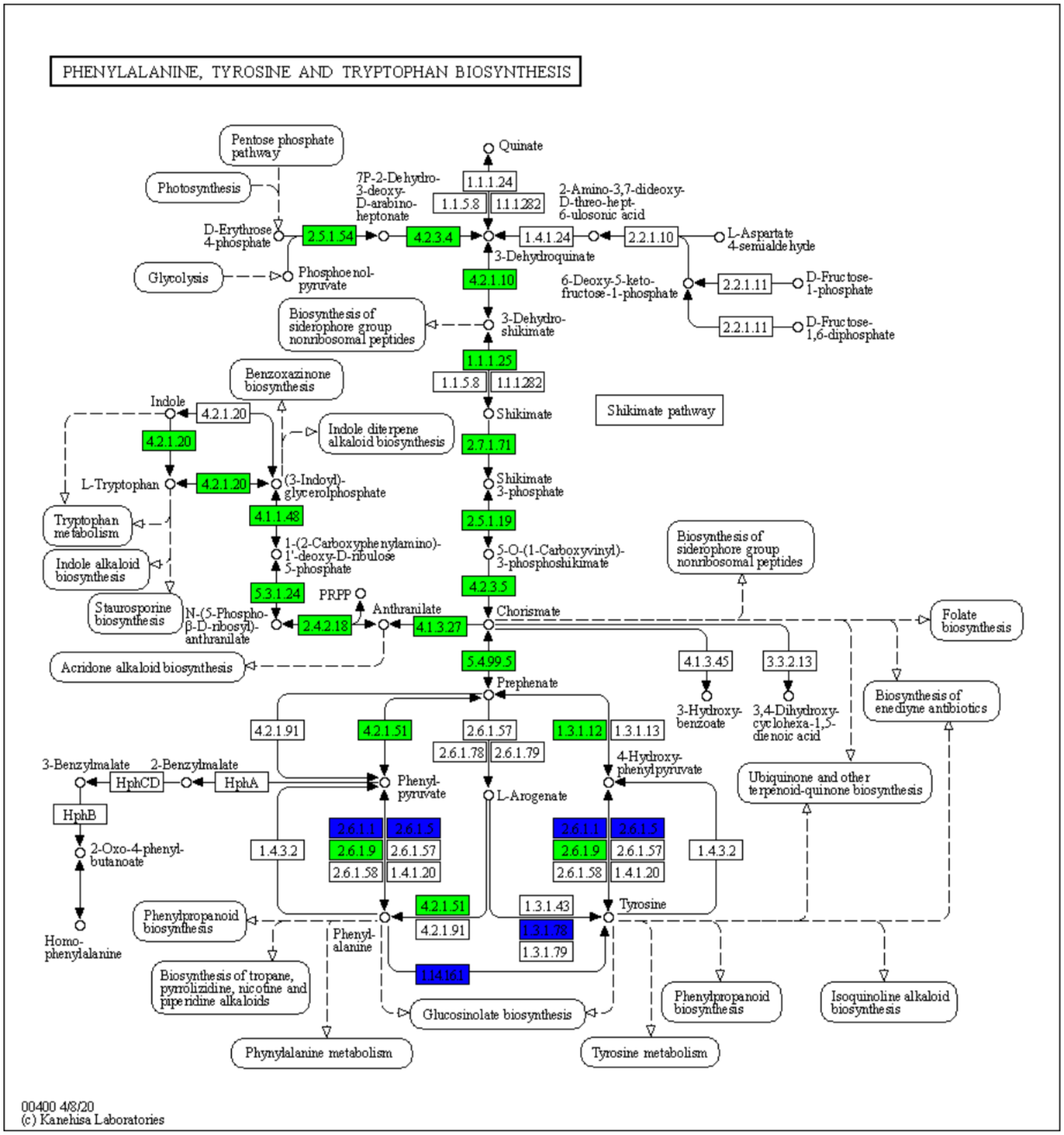
PHENYLALANINE, TYROSINE AND TRYPTOPHAN BIOSYNTHESIS
Pentose phosphate pathway
Photosynthesis
Glycolysis
D-Erythrose 4-phosphate
2.5.1.54
Phosphoenol-pyruvate
7P-2-Dehydro-3-deoxy-D-arabino-heptonate
4.2.3.4
Quinate
1.1.1.24
1.1.5.8
1.1.1.282
2-Amino-3,7-dideoxy-D-threo-hept-6-ulosonic acid
1.4.1.24
2.2.1.10
L-Aspartate 4-semialdehyde
3-Dehydroquinate
4.2.1.10
6-Deoxy-5-keto-fructose-1-phosphate
2.2.1.11
D-Fructose-1-phosphate
D-Fructose-1,6-diphosphate
Biosynthesis of siderophore group nonribosomal peptides
3-Dehydro-shikimate
1.1.1.25
Shikimate
Shikimate pathway
2.7.1.71
Shikimate 3-phosphate
2.5.1.19
5-O-(1-Carboxyvinyl)-3-phosphoshikimate
4.2.3.5
Chorismate
Benzoxazinone biosynthesis
Indole diterpene alkaloid biosynthesis
Indole
4.2.1.20
L-Tryptophan
(3-Indoyl)-glycerolphosphate
Tryptophan metabolism
Indole alkaloid biosynthesis
Staurosporine biosynthesis
4.1.1.48
1-(2-Carboxyphenylamino)-1'-deoxy-D-ribulose 5-phosphate
5.3.1.24
PRPP
Anthranilate
N-(5-Phospho-β-D-ribosyl)-anthranilate
2.4.2.18
4.1.3.27
Acridone alkaloid biosynthesis
Folate biosynthesis
5.4.99.5
4.1.3.45
3.3.2.13
Prephenate
3-Hydroxy-benzoate
3,4-Dihydroxy-cyclohexa-1,5-dienoic acid
Biosynthesis of enediyne antibiotics
4.2.1.91
4.2.1.51
2.6.1.57
2.6.1.78
2.6.1.79
1.3.1.12
1.3.1.13
4-Hydroxy-phenylpyruvate
3-Benzylmalate
2-Benzylmalate
HphCD
HphA
HphB
Phenyl-pyruvate
L-Arogenate
Ubiquinone and other terpenoid-quinone biosynthesis
2-Oxo-4-phenyl-butanoate
1.4.3.2
2.6.1.1
2.6.1.5
2.6.1.9
2.6.1.58
1.4.1.20
1.3.1.43
1.3.1.78
1.3.1.79
Tyrosine
Phenylpropanoid biosynthesis
Phenyl-alanine
1.14.16.1
Homo-phenylalanine
Biosynthesis of tropane, pyrrolizidine, nicotine and piperidine alkaloids
Glucosinolate biosynthesis
Isoquinoline alkaloid biosynthesis
Phynylalanine metabolism
Tyrosine metabolism
00400 4/8/20
(c) Kanehisa Laboratories

Carbohydrate metabolism

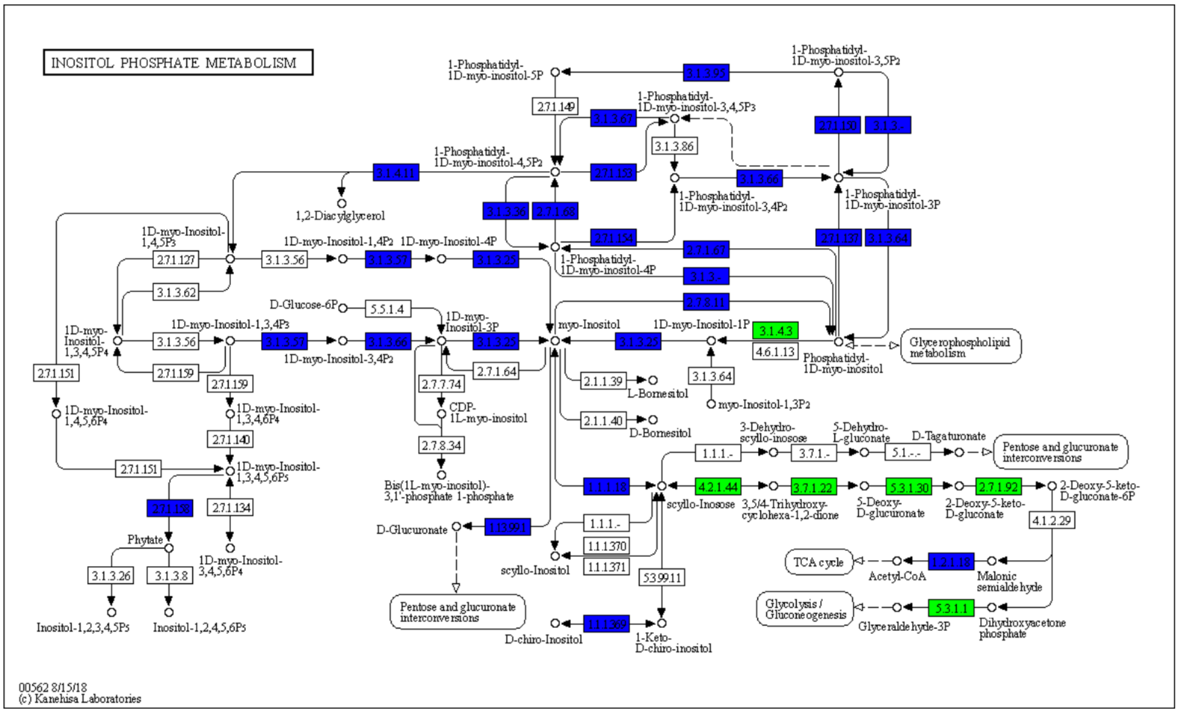

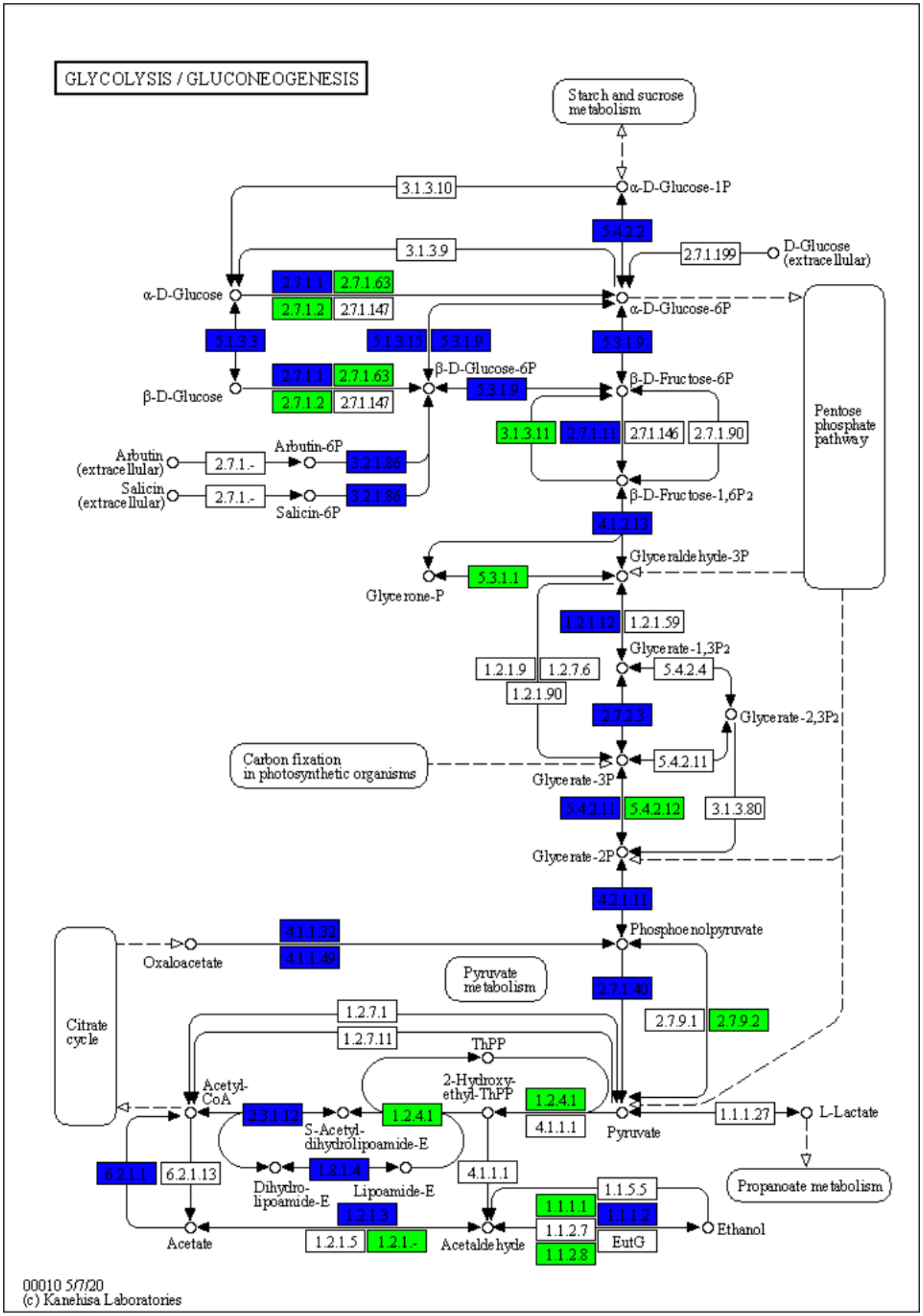
GLYCOLYSIS / GLUCONEOGENESIS
Starch and sucrose metabolism
α-D-Glucose-1P
3.1.3.10
5.4.2.2
3.1.3.9
2.7.1.199
D-Glucose (extracellular)
2.7.1.1
2.7.1.63
2.7.1.2
2.7.1.147
α-D-Glucose
α-D-Glucose-6P
5.1.3.3
5.1.3.15
5.3.1.9
β-D-Glucose-6P
β-D-Glucose
β-D-Fructose-6P
3.1.3.11
2.7.1.11
2.7.1.146
2.7.1.90
Pentose phosphate pathway
Arbutin (extracellular)
2.7.1.-
Arbutin-6P
3.2.1.86
Salicin (extracellular)
Salicin-6P
β-D-Fructose-1,6P2
4.1.2.13
Glyceraldehyde-3P
5.3.1.1
Glycerone-P
1.2.1.12
1.2.1.59
Glycerate-1,3P2
1.2.1.9
1.2.7.6
5.4.2.4
1.2.1.90
2.7.2.3
Glycerate-2,3P2
Carbon fixation in photosynthetic organisms
5.4.2.11
Glycerate-3P
5.4.2.12
3.1.3.80
Glycerate-2P
4.2.1.11
Phosphoenolpyruvate
4.1.1.32
4.1.1.49
Oxaloacetate
Citrate cycle
Pyruvate metabolism
2.7.1.40
1.2.7.1
1.2.7.11
2.7.9.1
2.7.9.2
ThPP
Acetyl-CoA
2-Hydroxy-ethyl-ThPP
1.2.4.1
2.3.1.12
S-Acetyl-dihydrolipoamide-E
4.1.1.1
Pyruvate
1.1.1.27
L-Lactate
Propanoate metabolism
6.2.1.1
6.2.1.13
1.8.1.4
Dihydro-lipoamide-E
Lipoamide-E
1.1.5.5
1.1.1.1
1.1.1.2
1.2.1.3
1.1.2.7
Ethanol
Acetate
1.2.1.5
1.2.1.-
Acetaldehyde
EutG
1.1.2.8
00010 5/7/20
(c) Kanehisa Laboratories

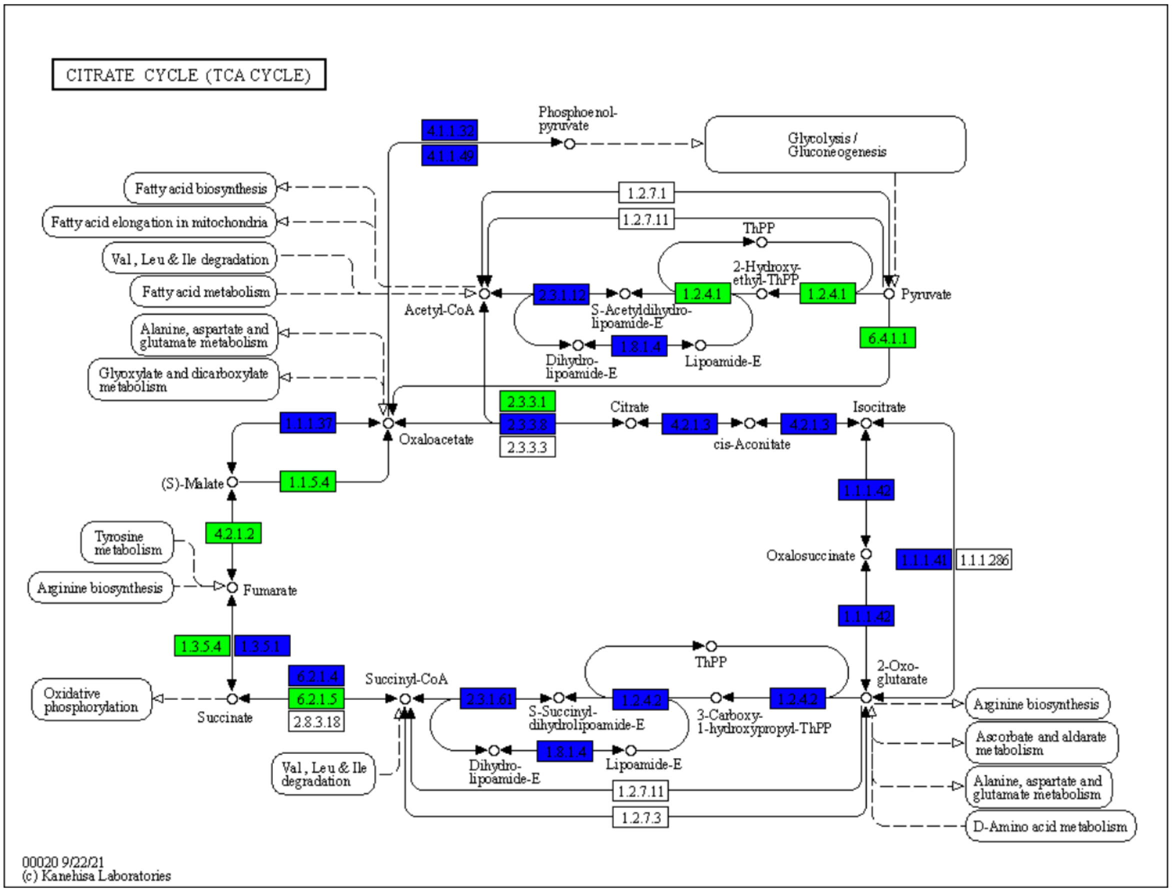
CITRATE CYCLE (TCA CYCLE)
Phosphoenol-pyruvate
Glycolysis / Gluconeogenesis
Fatty acid biosynthesis
Fatty acid elongation in mitochondria
Val, Leu & Ile degradation
Fatty acid metabolism
Alanine, aspartate and glutamate metabolism
Glyoxylate and dicarboxylate metabolism
Acetyl-CoA
S-Acetyldihydro-lipoamide-E
2-Hydroxy-ethyl-ThPP
ThPP
Pyruvate
Dihydro-lipoamide-E
Lipoamide-E
Oxaloacetate
Citrate
cis-Aconitate
Isocitrate
(S)-Malate
Oxalosuccinate
Tyrosine metabolism
Arginine biosynthesis
Fumarate
Oxidative phosphorylation
Succinate
Succinyl-CoA
S-Succinyl-dihydrolipoamide-E
3-Carboxy-1-hydroxypropyl-ThPP
2-Oxo-glutarate
Ascorbate and aldarate metabolism
D-Amino acid metabolism
4.1.1.32
4.1.1.49
1.2.7.1
1.2.7.11
2.3.1.12
1.2.4.1
6.4.1.1
1.8.1.4
2.3.3.1
2.3.3.8
2.3.3.3
4.2.1.3
1.1.1.37
1.1.5.4
1.1.1.42
4.2.1.2
1.1.1.41
1.1.1.286
1.3.5.4
1.3.5.1
6.2.1.4
6.2.1.5
2.8.3.18
2.3.1.61
1.2.4.2
1.2.7.3
00020 9/22/21
(c) Kanehisa Laboratories

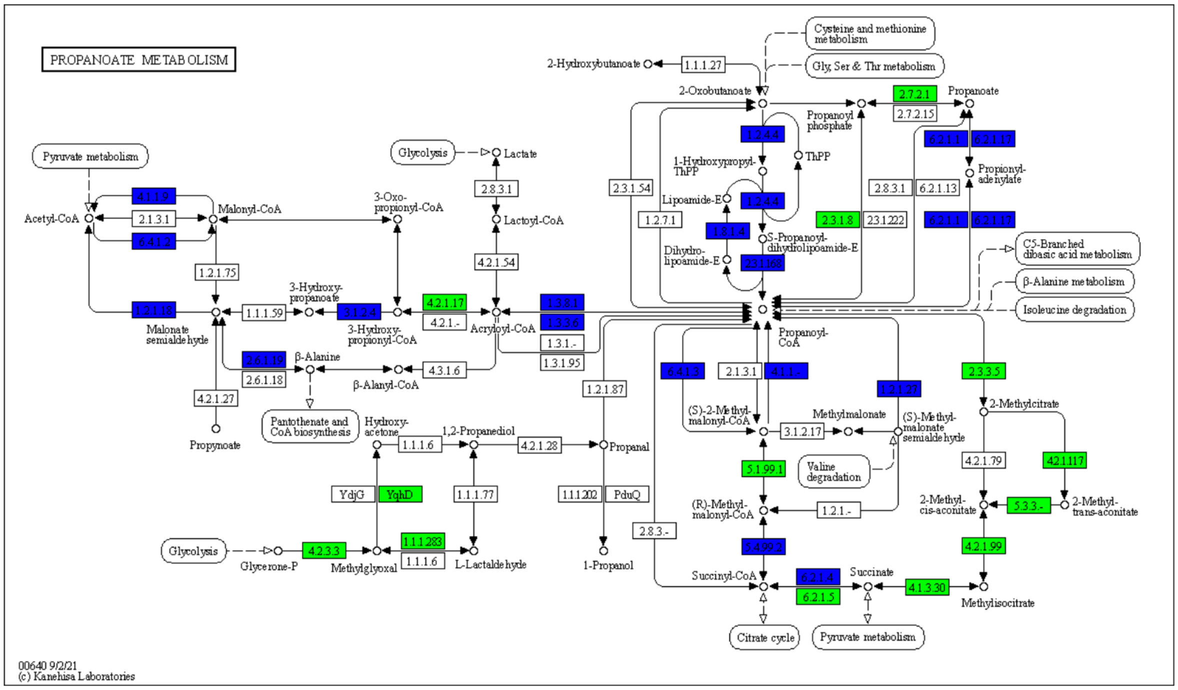
PROPANOATE METABOLISM
Cysteine and methionine metabolism
Gly, Ser & Thr metabolism
2-Hydroxybutanoate
2-Oxobutanoate
Propanoate
Propanoyl phosphate
Propionyl-adenylate
1-Hydroxypropyl-ThPP
ThPP
Lipoamide-E
Dihydro-lipoamide-E
S-Propanoyl-dihydrolipoamide-E
C5-Branched dibasic acid metabolism
β-Alanine metabolism
Isoleucine degradation
Pyruvate metabolism
Glycolysis
Lactate
Lactoyl-CoA
Acetyl-CoA
Malonyl-CoA
3-Oxo-propionyl-CoA
3-Hydroxy-propanoate
3-Hydroxy-propionyl-CoA
Acryloyl-CoA
Malonate semialdehyde
β-Alanine
β-Alanyl-CoA
Propanoyl-CoA
Propynoate
Pantothenate and CoA biosynthesis
Hydroxy-acetone
1,2-Propanediol
Propanal
Glycerone-P
Methylglyoxal
L-Lactaldehyde
1-Propanol
(S)-2-Methyl-malonyl-CoA
Methylmalonate
(S)-Methyl-malonate semialdehyde
Valine degradation
(R)-Methyl-malonyl-CoA
2-Methylcitrate
2-Methyl-cis-aconitate
2-Methyl-trans-aconitate
Succinyl-CoA
Succinate
Methylisocitrate
Citrate cycle
YdjG
YqhD
PduQ
00640 9/2/21
(c) Kanehisa Laboratories

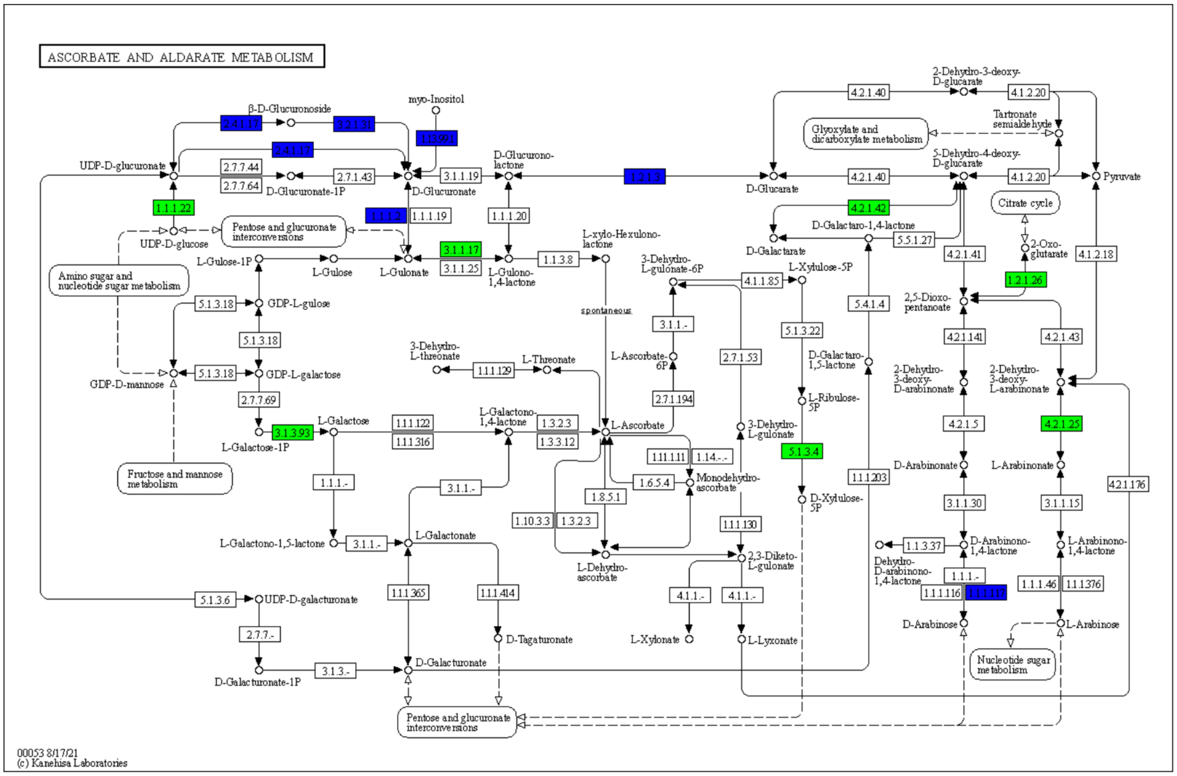
ASCORBATE AND ALDARATE METABOLISM
β-D-Glucuronoside
myo-Inositol
UDP-D-glucuronate
2.7.7.44
2.7.7.64
D-Glucuronate-1P
2.7.1.43
D-Glucuronate
3.1.1.19
D-Glucurono-lactone
1.1.1.22
UDP-D-glucose
Pentose and glucuronate interconversions
1.1.1.19
1.1.1.20
3.1.1.17
3.1.1.25
L-Gulono-1,4-lactone
1.1.3.8
L-xylo-Hexulono-lactone
L-Gulose-1P
L-Gulose
L-Gulonate
Amino sugar and nucleotide sugar metabolism
5.1.3.18
GDP-L-gulose
GDP-D-mannose
GDP-L-galactose
2.7.7.69
L-Galactose-1P
3.1.3.93
L-Galactose
1.1.1.122
1.1.1.316
L-Galactono-1,4-lactone
1.3.2.3
1.3.3.12
L-Ascorbate
3-Dehydro-L-threonate
1.1.1.129
L-Threonate
spontaneous
3-Dehydro-L-gulonate-6P
3.1.1.-
L-Ascorbate-6P
2.7.1.194
1.11.1.11
1.14.-.-
1.6.5.4
Monodehydro-ascorbate
1.8.5.1
1.10.3.3
L-Dehydro-ascorbate
Fructose and mannose metabolism
1.1.1.-
L-Galactono-1,5-lactone
L-Galactonate
1.1.1.365
1.1.1.414
D-Tagaturonate
5.1.3.6
UDP-D-galacturonate
2.7.7.-
D-Galacturonate-1P
3.1.3.-
D-Galacturonate
2.7.1.53
3-Dehydro-L-gulonate
1.1.1.130
2,3-Diketo-L-gulonate
4.1.1.-
L-Xylonate
L-Lyxonate
4.1.1.85
L-Xylulose-5P
5.1.3.22
L-Ribulose-5P
5.1.3.4
D-Xylulose-5P
D-Galactaro-1,5-lactone
1.1.1.203
5.4.1.4
D-Glucarate
4.2.1.40
D-Galactarate
4.2.1.42
D-Galactaro-1,4-lactone
5.5.1.27
2-Dehydro-3-deoxy-D-glucarate
4.1.2.20
Glyoxylate and dicarboxylate metabolism
Tartronate semialdehyde
5-Dehydro-4-deoxy-D-glucarate
Pyruvate
Citrate cycle
2-Oxo-glutarate
4.2.1.41
1.2.1.26
4.1.2.18
2,5-Dioxo-pentanoate
4.2.1.141
4.2.1.43
2-Dehydro-3-deoxy-D-arabinonate
2-Dehydro-3-deoxy-L-arabinonate
4.2.1.5
4.2.1.25
D-Arabinonate
L-Arabinonate
4.2.1.176
3.1.1.30
3.1.1.15
1.1.3.37
D-Arabinono-1,4-lactone
L-Arabinono-1,4-lactone
Dehydro-D-arabinono-1,4-lactone
1.1.1.-
1.1.1.116
1.1.1.46
1.1.1.376
D-Arabinose
L-Arabinose
Nucleotide sugar metabolism
Pentose and glucuronate interconversions
00053 8/17/21
(c) Kanehisa Laboratories

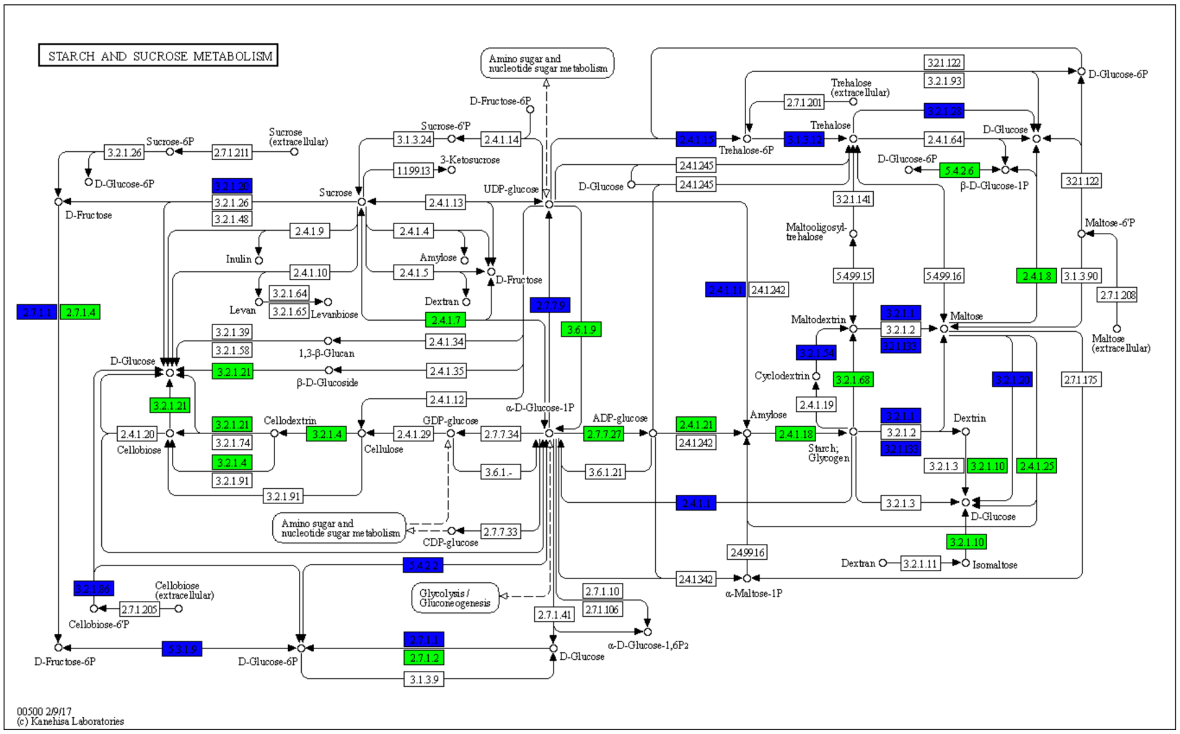
STARCH AND SUCROSE METABOLISM
Amino sugar and nucleotide sugar metabolism
D-Fructose-6P
Sucrose-6P
3.2.1.26
2.4.1.211
Sucrose (extracellular)
D-Glucose-6P
D-Fructose
3.2.1.26
3.2.1.48
Sucrose
3.1.3.24
2.4.1.14
1.1.99.13
3-Ketosucrose
2.4.1.13
UDP-glucose
2.4.1.9
Inulin
2.4.1.10
Levan
3.2.1.64
3.2.1.65
Levanbiose
2.4.1.4
Amylose
2.4.1.5
Dextran
D-Fructose
2.4.1.7
3.6.1.9
D-Glucose
3.2.1.39
3.2.1.58
1,3-β-Glucan
3.2.1.21
2.4.1.34
2.4.1.35
β-D-Glucoside
2.4.1.12
Cellodextrin
2.4.1.20
Cellobiose
3.2.1.74
3.2.1.4
3.2.1.91
Cellulose
2.4.1.29
GDP-glucose
2.7.7.34
α-D-Glucose-1P
3.6.1.-
Amino sugar and nucleotide sugar metabolism
2.7.7.33
CDP-glucose
Cellobiose (extracellular)
2.7.1.205
Cellobiose-6P
Glycolysis / Gluconeogenesis
2.7.1.10
2.7.1.106
2.7.1.41
α-D-Glucose-1,6P2
D-Fructose-6P
D-Glucose-6P
3.1.3.9
D-Glucose
D-Glucose
2.4.1.245
2.4.1.245
Trehalose-6P
Trehalose (extracellular)
2.7.1.201
Trehalose
3.2.1.122
3.2.1.93
D-Glucose-6P
2.4.1.64
D-Glucose
β-D-Glucose-1P
5.4.2.6
3.2.1.122
3.2.1.141
Maltooligosyl-trehalose
5.4.99.15
5.4.99.16
2.4.1.242
Maltose-6P
3.1.3.90
2.7.1.208
Maltodextrin
3.2.1.2
Maltose
Maltose (extracellular)
Cyclodextrin
3.2.1.68
2.7.1.175
2.4.1.19
ADP-glucose
2.7.7.27
2.4.1.21
2.4.1.242
Amylose
2.4.1.18
Starch; Glycogen
3.2.1.2
Dextrin
3.2.1.3
3.2.1.10
2.4.1.25
3.6.1.21
3.2.1.3
D-Glucose
2.4.99.16
2.4.1.342
α-Maltose-1P
3.2.1.10
Dextran
3.2.1.11
Isomaltose
3.1.3.9
00500 2/9/17
(c) Kanehisa Laboratories

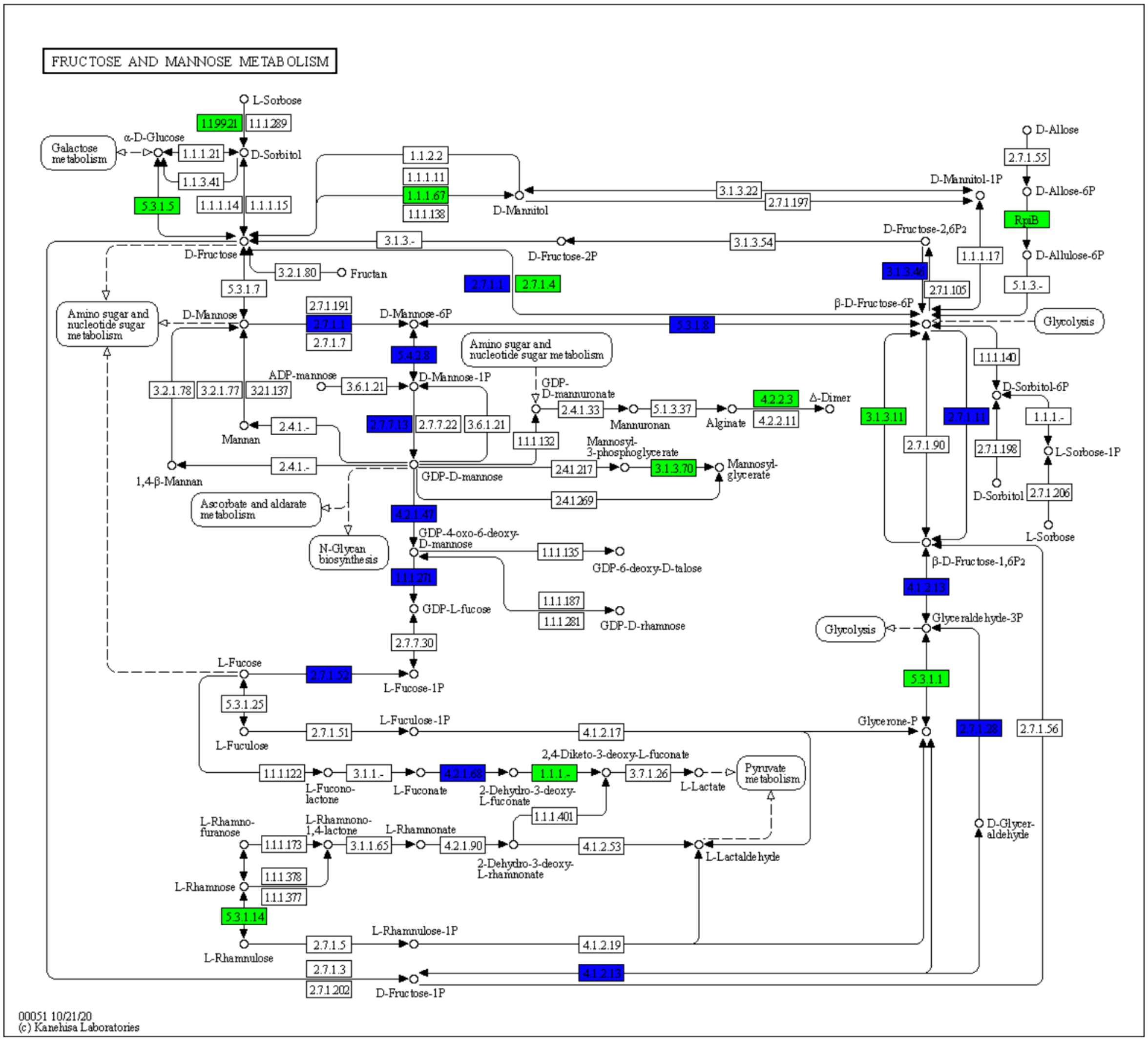
FRUCTOSE AND MANNOSE METABOLISM
L-Sorbose
1.1.99.21
1.1.1.289
α-D-Glucose
Galactose metabolism
1.1.1.21
D-Sorbitol
1.1.3.41
5.3.1.5
1.1.1.14
1.1.1.15
1.1.2.2
1.1.1.11
1.1.1.67
1.1.1.138
D-Mannitol
3.1.3.22
2.7.1.197
D-Mannitol-1P
D-Allose
2.7.1.55
D-Allose-6P
RpiB
D-Allulose-6P
5.1.3.-
3.1.3.-
D-Fructose
D-Fructose-2P
3.1.3.54
D-Fructose-2,6P2
1.1.1.17
3.2.1.80
Fructan
5.3.1.7
2.7.1.4
2.7.1.105
β-D-Fructose-6P
Glycolysis
Amino sugar and nucleotide sugar metabolism
D-Mannose
2.7.1.191
2.7.1.7
D-Mannose-6P
Amino sugar and nucleotide sugar metabolism
ADP-mannose
3.6.1.21
D-Mannose-1P
3.2.1.78
3.2.1.77
3.2.1.137
GDP-D-mannuronate
2.4.1.33
Mannuronan
5.1.3.37
Alginate
4.2.2.3
4.2.2.11
Δ-Dimer
3.1.3.11
1.1.1.140
D-Sorbitol-6P
1.1.1.-
Mannan
2.4.1.-
2.7.7.22
3.6.1.21
1.1.1.132
Mannosyl-3-phosphoglycerate
2.7.1.90
2.7.1.198
L-Sorbose-1P
1,4-β-Mannan
2.4.1.-
GDP-D-mannose
2.4.1.217
3.1.3.70
Mannosyl-glycerate
D-Sorbitol
2.7.1.206
Ascorbate and aldarate metabolism
2.4.1.269
L-Sorbose
N-Glycan biosynthesis
GDP-4-oxo-6-deoxy-D-mannose
1.1.1.135
GDP-6-deoxy-D-talose
β-D-Fructose-1,6P2
1.1.1.187
1.1.1.281
GDP-D-rhamnose
GDP-L-fucose
Glyceraldehyde-3P
Glycolysis
2.7.7.30
L-Fucose
L-Fucose-1P
5.3.1.1
5.3.1.25
L-Fuculose-1P
L-Fuculose
2.7.1.51
4.1.2.17
Glycerone-P
2.7.1.56
2,4-Diketo-3-deoxy-L-fuconate
1.1.1.122
3.1.1.-
1.1.1.-
3.7.1.26
L-Fucono-lactone
L-Fuconate
2-Dehydro-3-deoxy-L-fuconate
L-Lactate
Pyruvate metabolism
1.1.1.401
L-Rhamno-furanose
L-Rhamnono-1,4-lactone
L-Rhamnonate
1.1.1.173
3.1.1.65
4.2.1.90
4.1.2.53
L-Lactaldehyde
D-Glycer-aldehyde
2-Dehydro-3-deoxy-L-rhamnonate
L-Rhamnose
1.1.1.378
1.1.1.377
5.3.1.14
L-Rhamnulose-1P
L-Rhamnulose
2.7.1.5
4.1.2.19
2.7.1.3
2.7.1.202
D-Fructose-1P
00051 10/21/20
(c) Kanehisa Laboratories

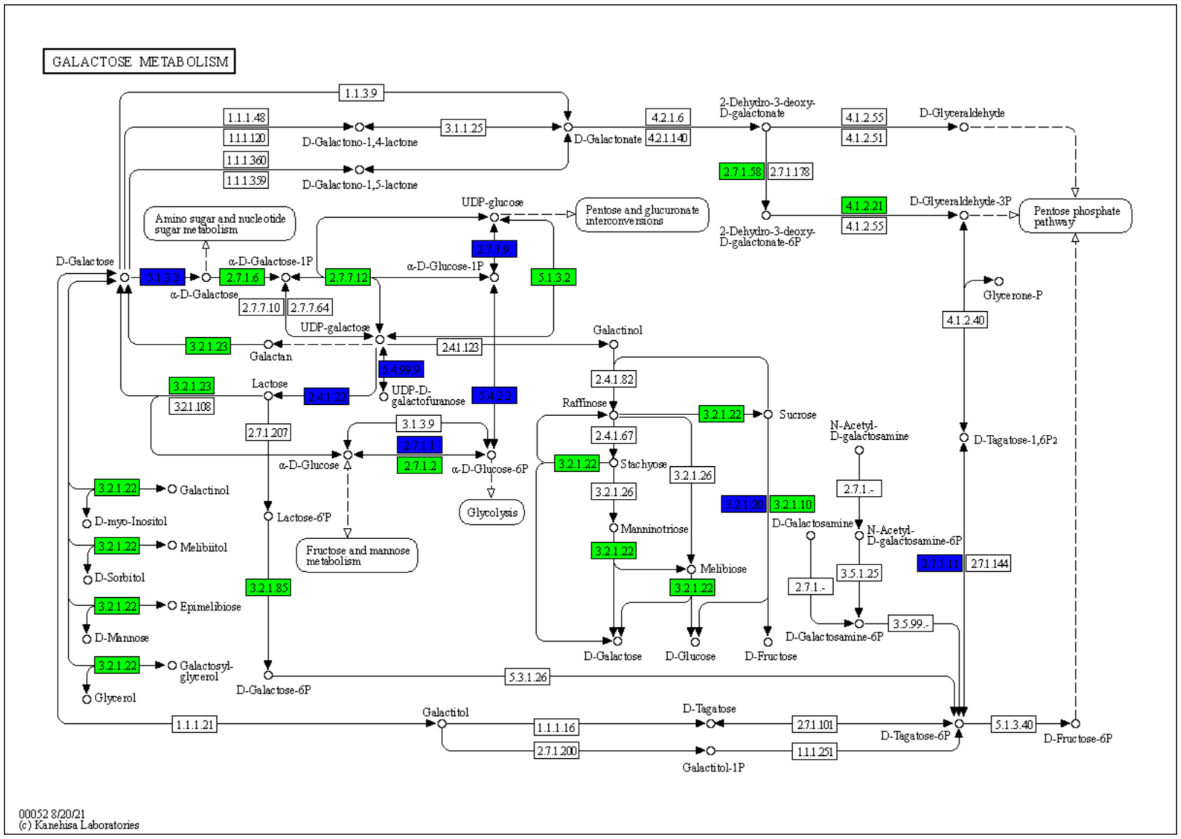
GALACTOSE METABOLISM
D-Galactose
α-D-Galactose
α-D-Galactose-1P
UDP-glucose
α-D-Glucose-1P
UDP-galactose
Galactan
Lactose
UDP-D-galactofuranose
α-D-Glucose
α-D-Glucose-6P
Lactose-6P
D-Galactose-6P
Galactinol
Raffinose
Stachyose
Manninotriose
Melibiose
Sucrose
D-Glucose
D-Fructose
D-Galactonate
D-Galactono-1,4-lactone
D-Galactono-1,5-lactone
2-Dehydro-3-deoxy-D-galactonate
2-Dehydro-3-deoxy-D-galactonate-6P
D-Glyceraldehyde
D-Glyceraldehyde-3P
Glycerone-P
D-Tagatose-1,6P2
N-Acetyl-D-galactosamine
N-Acetyl-D-galactosamine-6P
D-Galactosamine
D-Galactosamine-6P
D-Tagatose-6P
D-Fructose-6P
D-Tagatose
Galactitol
Galactitol-1P
D-myo-Inositol
Melibiitol
D-Sorbitol
Epimelibiose
D-Mannose
Galactosyl-glycerol
Glycerol
Amino sugar and nucleotide sugar metabolism
Pentose and glucuronate interconversions
Pentose phosphate pathway
Fructose and mannose metabolism
Glycolysis
1.1.3.9
1.1.1.48
1.1.1.120
3.1.1.25
1.1.1.360
1.1.1.359
4.2.1.6
4.2.1.140
4.1.2.55
4.1.2.51
2.7.1.58
2.7.1.178
4.1.2.21
5.1.3.3
2.7.1.6
2.7.7.12
2.7.7.9
5.1.3.2
2.7.7.10
2.7.7.64
3.2.1.23
2.4.1.123
5.4.99.9
2.4.1.22
3.2.1.108
5.4.2.2
2.7.1.207
3.1.3.9
2.7.1.1
2.7.1.2
2.4.1.82
2.4.1.67
3.2.1.22
3.2.1.26
3.2.1.10
2.7.1.-
3.5.1.25
3.5.99.-
2.7.1.11
2.7.1.144
4.1.2.40
3.2.1.85
5.3.1.26
1.1.1.21
1.1.1.16
2.7.1.101
2.7.1.200
1.1.1.251
5.1.3.40
00052 8/20/21
(c) Kanehisa Laboratories

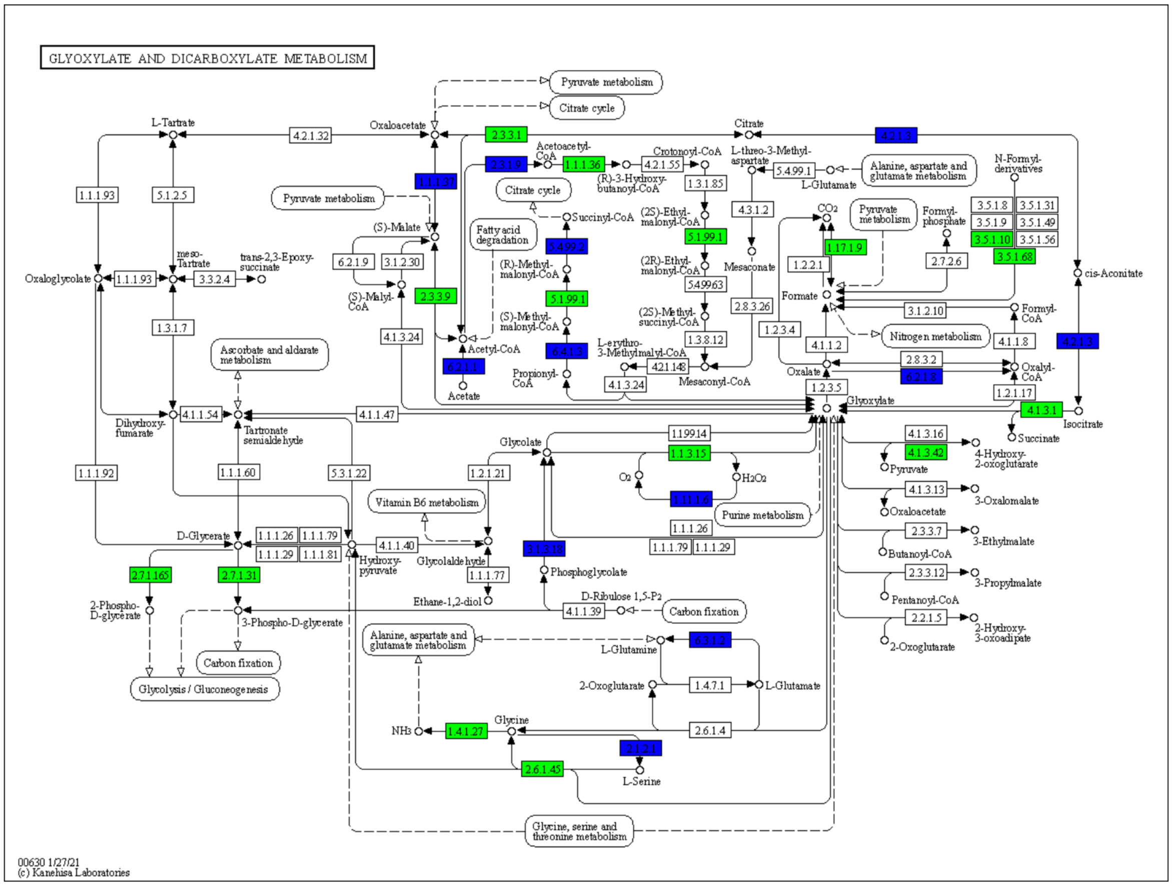

GLYOXYLATE AND DICARBOXYLATE METABOLISM
Pyruvate metabolism
Citrate cycle
L-Tartrate
4.2.1.32
Oxaloacetate
2.3.3.1
Citrate
Acetoacetyl-CoA
1.1.1.36
(R)-3-Hydroxy-butanoyl-CoA
4.2.1.55
Crotonoyl-CoA
1.3.1.85
L-threo-3-Methyl-aspartate
5.4.99.1
L-Glutamate
Alanine, aspartate and glutamate metabolism
N-Formyl-derivatives
1.1.1.93
5.1.2.5
Citrate cycle
Succinyl-CoA
(2S)-Ethyl-malonyl-CoA
4.3.1.2
CO2
Pyruvate metabolism
Formyl-phosphate
3.5.1.8
3.5.1.31
3.5.1.9
3.5.1.49
3.5.1.10
3.5.1.56
3.5.1.68
(S)-Malate
Fatty acid degradation
5.1.99.1
1.17.1.9
6.2.1.9
3.1.2.30
(R)-Methyl-malonyl-CoA
(2R)-Ethyl-malonyl-CoA
Mesaconate
1.2.2.1
2.7.2.6
Oxaloglycolate
1.1.1.93
meso-Tartrate
3.3.2.4
trans-2,3-Epoxy-succinate
(S)-Malyl-CoA
2.3.3.9
5.4.99.63
Formate
cis-Aconitate
5.1.99.1
(S)-Methyl-malonyl-CoA
(2S)-Methyl-succinyl-CoA
2.8.3.26
3.1.2.10
Formyl-CoA
1.3.1.7
4.1.3.24
Acetyl-CoA
1.3.8.12
1.2.3.4
Nitrogen metabolism
Ascorbate and aldarate metabolism
L-erythro-3-Methylmalyl-CoA
4.2.1.148
4.1.1.2
4.1.1.8
Acetate
Propionyl-CoA
4.1.3.24
Mesaconyl-CoA
Oxalate
2.8.3.2
Oxalyl-CoA
1.2.3.5
1.2.1.17
Dihydroxy-fumarate
4.1.1.54
Tartronate semialdehyde
4.1.1.47
Glyoxylate
4.1.3.1
Isocitrate
Succinate
1.1.99.14
4.1.3.16
Glycolate
1.1.3.15
4.1.3.42
4-Hydroxy-2-oxoglutarate
1.1.1.92
1.1.1.60
5.3.1.22
1.2.1.21
O2
H2O2
Pyruvate
4.1.3.13
3-Oxalomalate
Vitamin B6 metabolism
Purine metabolism
Oxaloacetate
D-Glycerate
1.1.1.26
1.1.1.79
1.1.1.29
1.1.1.81
4.1.1.40
1.1.1.26
2.3.3.7
3-Ethylmalate
Hydroxy-pyruvate
Glycolaldehyde
1.1.1.79
1.1.1.29
Butanoyl-CoA
2.7.1.165
2.7.1.31
1.1.1.77
Phosphoglycolate
2.3.3.12
3-Propylmalate
2-Phospho-D-glycerate
Ethane-1,2-diol
D-Ribulose 1,5-P2
4.1.1.39
Carbon fixation
Pentanoyl-CoA
3-Phospho-D-glycerate
2.2.1.5
2-Hydroxy-3-oxoadipate
Alanine, aspartate and glutamate metabolism
L-Glutamine
2-Oxoglutarate
Carbon fixation
Glycolysis / Gluconeogenesis
2-Oxoglutarate
1.4.7.1
L-Glutamate
Glycine
NH3
1.4.1.27
2.6.1.4
2.6.1.45
L-Serine
Glycine, serine and threonine metabolism
00630 1/27/21
(c) Kanehisa Laboratories

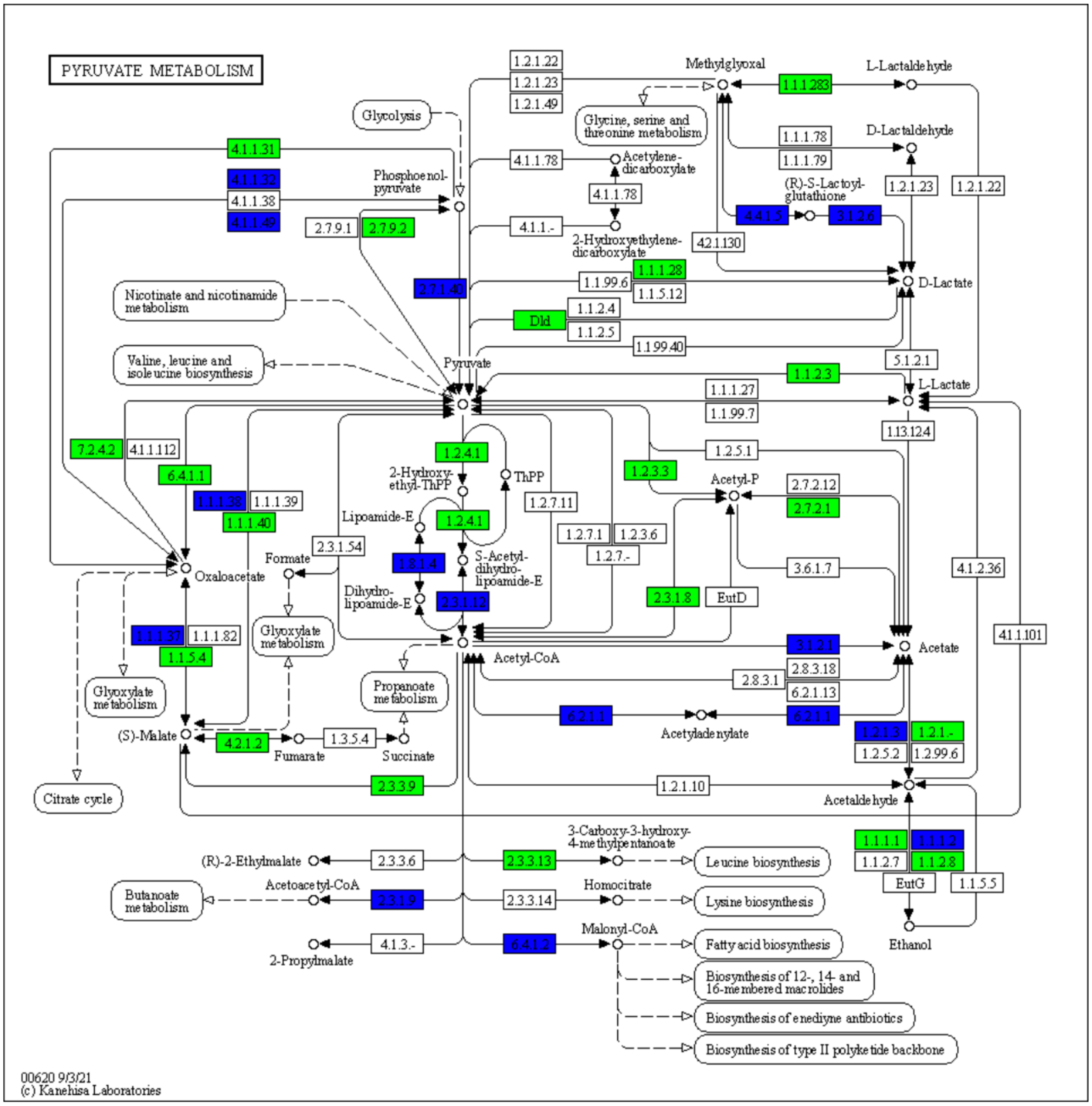
PYRUVATE METABOLISM
Glycolysis
Phosphoenol-pyruvate
Methylglyoxal
L-Lactaldehyde
D-Lactaldehyde
Glycine, serine and threonine metabolism
Acetylene-dicarboxylate
2-Hydroxyethylene-dicarboxylate
(R)-S-Lactoyl-glutathione
D-Lactate
L-Lactate
Nicotinate and nicotinamide metabolism
Valine, leucine and isoleucine biosynthesis
Pyruvate
Oxaloacetate
Formate
Glyoxylate metabolism
2-Hydroxy-ethyl-ThPP
ThPP
Lipoamide-E
S-Acetyl-dihydro-lipoamide-E
Dihydro-lipoamide-E
Acetyl-P
Acetyl-CoA
Acetate
Propanoate metabolism
Acetyladenylate
(S)-Malate
Fumarate
Succinate
Citrate cycle
Acetaldehyde
Ethanol
(R)-2-Ethylmalate
3-Carboxy-3-hydroxy-4-methylpentanoate
Leucine biosynthesis
Butanoate metabolism
Acetoacetyl-CoA
Homocitrate
Lysine biosynthesis
2-Propylmalate
Malonyl-CoA
Fatty acid biosynthesis
Biosynthesis of 12-, 14- and 16-membered macrolides
Biosynthesis of enediyne antibiotics
Biosynthesis of type II polyketide backbone
1.2.1.22
1.2.1.23
1.2.1.49
1.1.1.283
1.1.1.78
1.1.1.79
4.1.1.78
4.1.1.-
4.2.1.130
4.4.1.5
3.1.2.6
1.2.1.23
1.2.1.22
4.1.1.31
4.1.1.32
4.1.1.38
4.1.1.49
2.7.9.1
2.7.9.2
2.7.1.40
1.1.1.28
1.1.99.6
1.1.5.12
Dld
1.1.2.4
1.1.2.5
1.1.99.40
5.1.2.1
1.1.2.3
1.1.1.27
1.1.99.7
1.13.12.4
7.2.4.2
4.1.1.112
6.4.1.1
1.1.1.38
1.1.1.39
1.1.1.40
1.2.4.1
1.2.3.3
1.2.5.1
2.7.2.12
2.7.2.1
1.2.7.11
2.3.1.54
1.2.7.1
1.2.3.6
1.2.7.-
1.8.1.4
2.3.1.12
3.6.1.7
4.1.2.36
2.3.1.8
EutD
3.1.2.1
4.1.1.101
1.1.1.37
1.1.1.82
1.1.5.4
2.8.3.1
2.8.3.18
6.2.1.13
6.2.1.1
6.2.1.1
4.2.1.2
1.3.5.4
1.2.1.3
1.2.1.-
1.2.5.2
1.2.99.6
2.3.3.9
1.2.1.10
1.1.1.1
1.1.1.2
1.1.2.7
1.1.2.8
EutG
1.1.5.5
2.3.3.6
2.3.3.13
2.3.1.9
2.3.3.14
4.1.3.-
6.4.1.2
00620 9/3/21
(c) Kanehisa Laboratories

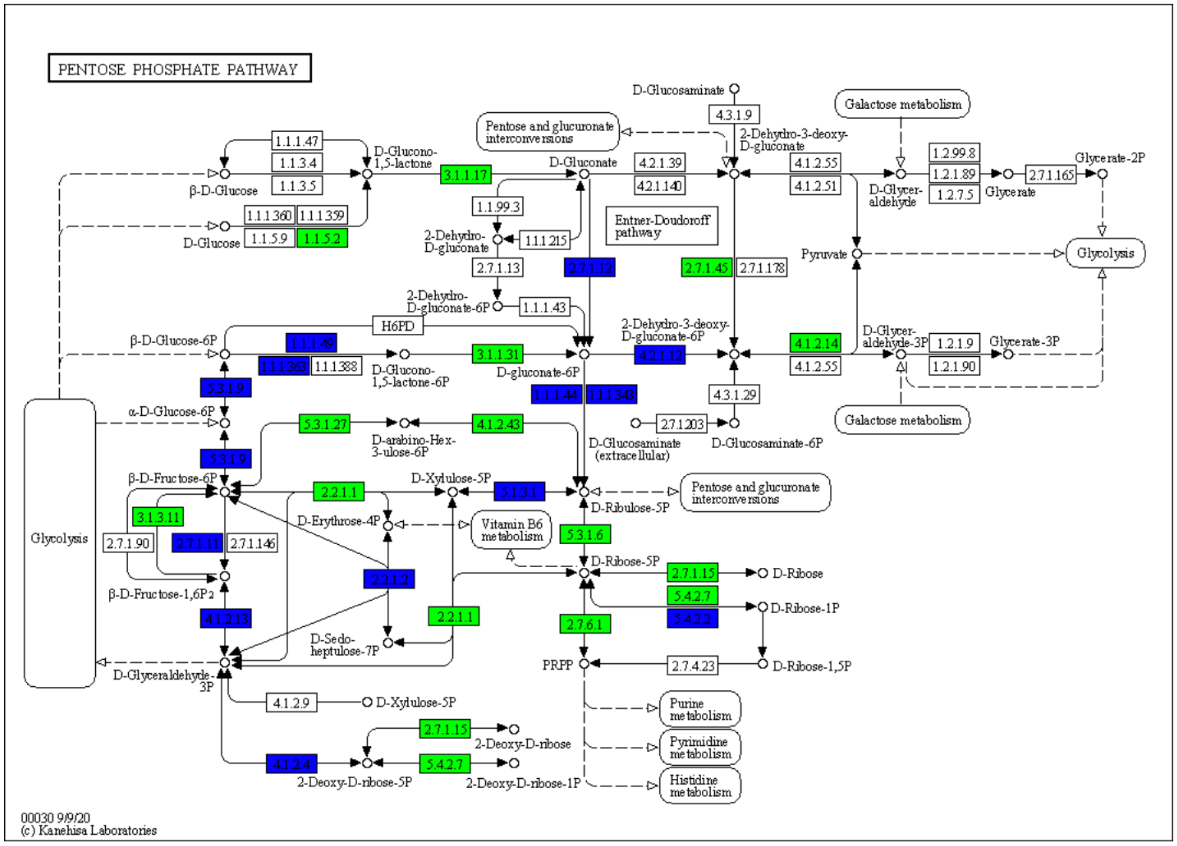
PENTOSE PHOSPHATE PATHWAY
D-Glucosaminate
4.3.1.9
Galactose metabolism
Pentose and glucuronate interconversions
2-Dehydro-3-deoxy-D-gluconate
1.1.1.47
1.1.3.4
1.1.3.5
D-Glucono-1,5-lactone
3.1.1.17
D-Gluconate
4.2.1.39
4.2.1.140
4.1.2.55
4.1.2.51
1.2.99.8
1.2.1.89
1.2.7.5
2.7.1.165
Glycerate-2P
Glycerate
D-Glycer-aldehyde
β-D-Glucose
1.1.99.3
1.1.1.360
1.1.1.359
D-Glucose
1.1.5.9
1.1.5.2
2-Dehydro-D-gluconate
1.1.1.215
Entner-Doudoroff pathway
Pyruvate
Glycolysis
2.7.1.13
2.7.1.12
2.7.1.45
2.7.1.178
2-Dehydro-D-gluconate-6P
1.1.1.43
H6PD
β-D-Glucose-6P
1.1.1.49
1.1.1.363
1.1.1.388
D-Glucono-1,5-lactone-6P
3.1.1.31
D-gluconate-6P
2-Dehydro-3-deoxy-D-gluconate-6P
4.2.1.12
4.1.2.14
4.1.2.55
D-Glycer-aldehyde-3P
1.2.1.9
1.2.1.90
Glycerate-3P
5.3.1.9
1.1.1.44
1.1.1.343
4.3.1.29
α-D-Glucose-6P
5.3.1.27
D-arabino-Hex-3-ulose-6P
4.1.2.43
2.7.1.203
D-Glucosaminate (extracellular)
D-Glucosamine-6P
β-D-Fructose-6P
2.2.1.1
D-Xylulose-5P
5.1.3.1
D-Ribulose-5P
Pentose and glucuronate interconversions
3.1.3.11
D-Erythrose-4P
Vitamin B6 metabolism
5.3.1.6
Glycolysis
2.7.1.90
2.7.1.11
2.7.1.146
D-Ribose-5P
2.7.1.15
D-Ribose
2.2.1.2
5.4.2.7
D-Ribose-1P
β-D-Fructose-1,6P2
5.4.2.2
4.1.2.13
2.2.1.1
2.7.6.1
D-Sedo-heptulose-7P
PRPP
2.7.4.23
D-Ribose-1,5P
D-Glyceraldehyde-3P
4.1.2.9
D-Xylulose-5P
Purine metabolism
2.7.1.15
2-Deoxy-D-ribose
Pyrimidine metabolism
4.1.2.4
5.4.2.7
2-Deoxy-D-ribose-5P
2-Deoxy-D-ribose-1P
Histidine metabolism
00030 9/9/20
(c) Kanehisa Laboratories

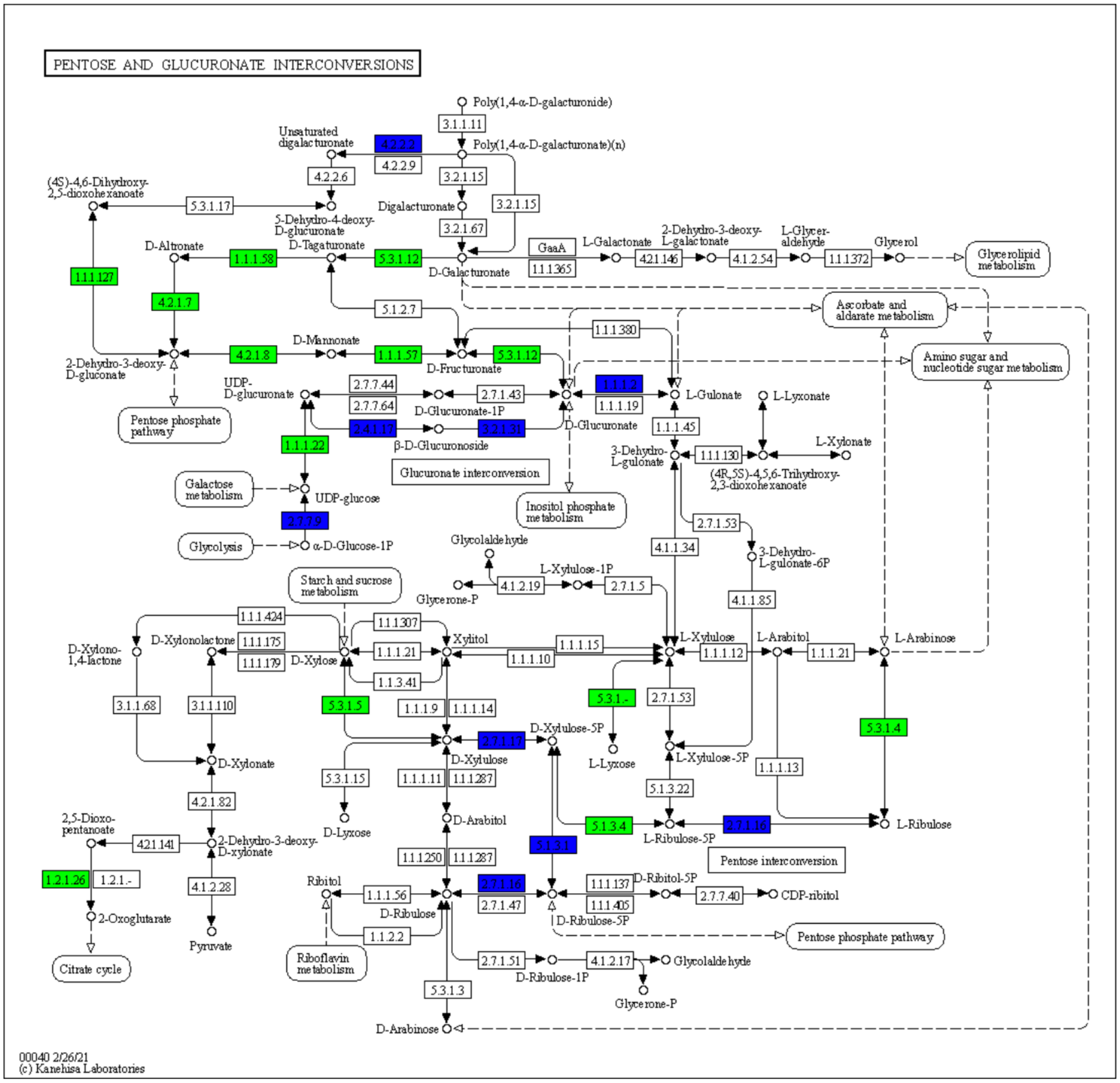
PENTOSE AND GLUCURONATE INTERCONVERSIONS
Poly(1,4-α-D-galacturonide)
3.1.1.11
Poly(1,4-α-D-galacturonate)(n)
Unsaturated digalacturonate
4.2.2.2
4.2.2.9
4.2.2.6
3.2.1.15
Digalacturonate
3.2.1.15
3.2.1.67
(4S)-4,6-Dihydroxy-2,5-dioxohexanoate
5.3.1.17
5-Dehydro-4-deoxy-D-glucuronate
D-Altronate
1.1.1.58
D-Tagaturonate
5.3.1.12
D-Galacturonate
GaaA
1.1.1.365
L-Galactonate
4.2.1.146
2-Dehydro-3-deoxy-L-galactonate
4.1.2.54
L-Glyceraldehyde
1.1.1.372
Glycerol
Glycerolipid metabolism
1.1.1.127
4.2.1.7
5.1.2.7
Ascorbate and aldarate metabolism
1.1.1.380
Amino sugar and nucleotide sugar metabolism
D-Mannonate
2-Dehydro-3-deoxy-D-gluconate
4.2.1.8
1.1.1.57
5.3.1.12
D-Fructuronate
Pentose phosphate pathway
UDP-D-glucuronate
2.7.7.44
2.7.7.64
2.7.1.43
D-Glucuronate-1P
1.1.1.19
L-Gulonate
L-Lyxonate
2.4.1.17
3.2.1.31
β-D-Glucuronoside
D-Glucuronate
1.1.1.45
L-Xylonate
1.1.1.22
3-Dehydro-L-gulonate
1.1.1.130
(4R,5S)-4,5,6-Trihydroxy-2,3-dioxohexanoate
Glucuronate interconversion
Galactose metabolism
UDP-glucose
Inositol phosphate metabolism
2.7.7.9
2.7.1.53
Glycolysis
α-D-Glucose-1P
4.1.1.34
3-Dehydro-L-gulonate-6P
Glycolaldehyde
L-Xylulose-1P
4.1.2.19
2.7.1.5
Glycerone-P
Starch and sucrose metabolism
4.1.1.85
1.1.1.424
1.1.1.307
Xylitol
1.1.1.15
L-Xylulose
L-Arabitol
L-Arabinose
D-Xylono-1,4-lactone
D-Xylonolactone
1.1.1.175
1.1.1.179
D-Xylose
1.1.1.21
1.1.1.10
1.1.1.12
1.1.1.21
1.1.3.41
5.3.1.-
2.7.1.53
3.1.1.68
3.1.1.110
5.3.1.5
1.1.1.9
1.1.1.14
2.7.1.17
D-Xylulose-5P
5.3.1.4
D-Xylonate
D-Xylulose
L-Lyxose
L-Xylulose-5P
5.3.1.15
1.1.1.11
1.1.1.287
1.1.1.13
5.1.3.22
4.2.1.82
D-Lyxose
D-Arabitol
5.1.3.4
L-Ribulose-5P
2.7.1.16
L-Ribulose
5.1.3.1
2,5-Dioxopentanoate
4.2.1.141
2-Dehydro-3-deoxy-D-xylonate
1.1.1.250
1.1.1.287
Pentose interconversion
1.2.1.26
1.2.1.-
4.1.2.28
Ribitol
1.1.1.56
2.7.1.47
1.1.1.137
D-Ribitol-5P
1.1.1.405
2.7.7.40
CDP-ribitol
2-Oxoglutarate
Pyruvate
D-Ribulose
D-Ribulose-5P
1.1.2.2
Pentose phosphate pathway
Citrate cycle
Riboflavin metabolism
2.7.1.51
D-Ribulose-1P
4.1.2.17
Glycolaldehyde
Glycerone-P
5.3.1.3
D-Arabinose
00040 2/26/21
(c) Kanehisa Laboratories

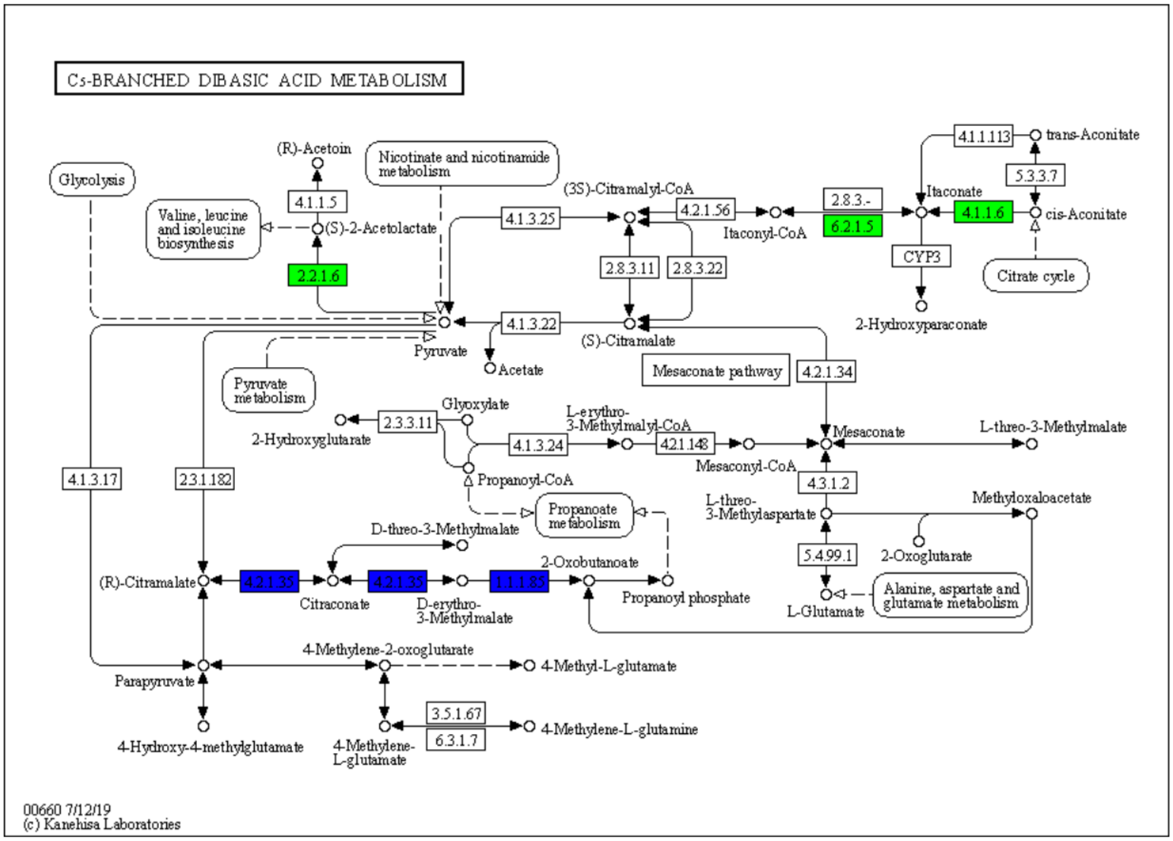
C5-BRANCHED DIBASIC ACID METABOLISM
Glycolysis
(R)-Acetoin
4.1.1.5
Valine, leucine and isoleucine biosynthesis
(S)-2-Acetolactate
2.2.1.6
Nicotinate and nicotinamide metabolism
(3S)-Citramalyl-CoA
4.1.3.25
4.2.1.56
Itaconyl-CoA
2.8.3.-
6.2.1.5
4.1.1.113
trans-Aconitate
5.3.3.7
Itaconate
4.1.1.6
cis-Aconitate
CYP3
Citrate cycle
2-Hydroxyparaconate
2.8.3.11
2.8.3.22
Pyruvate
4.1.3.22
(S)-Citramalate
Acetate
Mesaconate pathway
4.2.1.34
Pyruvate metabolism
Glyoxylate
2.3.3.11
2-Hydroxyglutarate
L-erythro-3-Methylmalyl-CoA
4.1.3.24
4.2.1.148
Mesaconyl-CoA
Mesaconate
L-threo-3-Methylmalate
Propanoyl-CoA
4.3.1.2
4.1.3.17
2.3.1.182
Propanoate metabolism
L-threo-3-Methylaspartate
Methyloxaloacetate
D-threo-3-Methylmalate
5.4.99.1
2-Oxoglutarate
(R)-Citramalate
4.2.1.35
Citraconate
4.2.1.35
D-erythro-3-Methylmalate
1.1.1.85
2-Oxobutanoate
Propanoyl phosphate
L-Glutamate
Alanine, aspartate and glutamate metabolism
Parapyruvate
4-Methylene-2-oxoglutarate
4-Methyl-L-glutamate
3.5.1.67
6.3.1.7
4-Methylene-L-glutamine
4-Hydroxy-4-methylglutamate
4-Methylene-L-glutamate
00660 7/12/19
(c) Kanehisa Laboratories

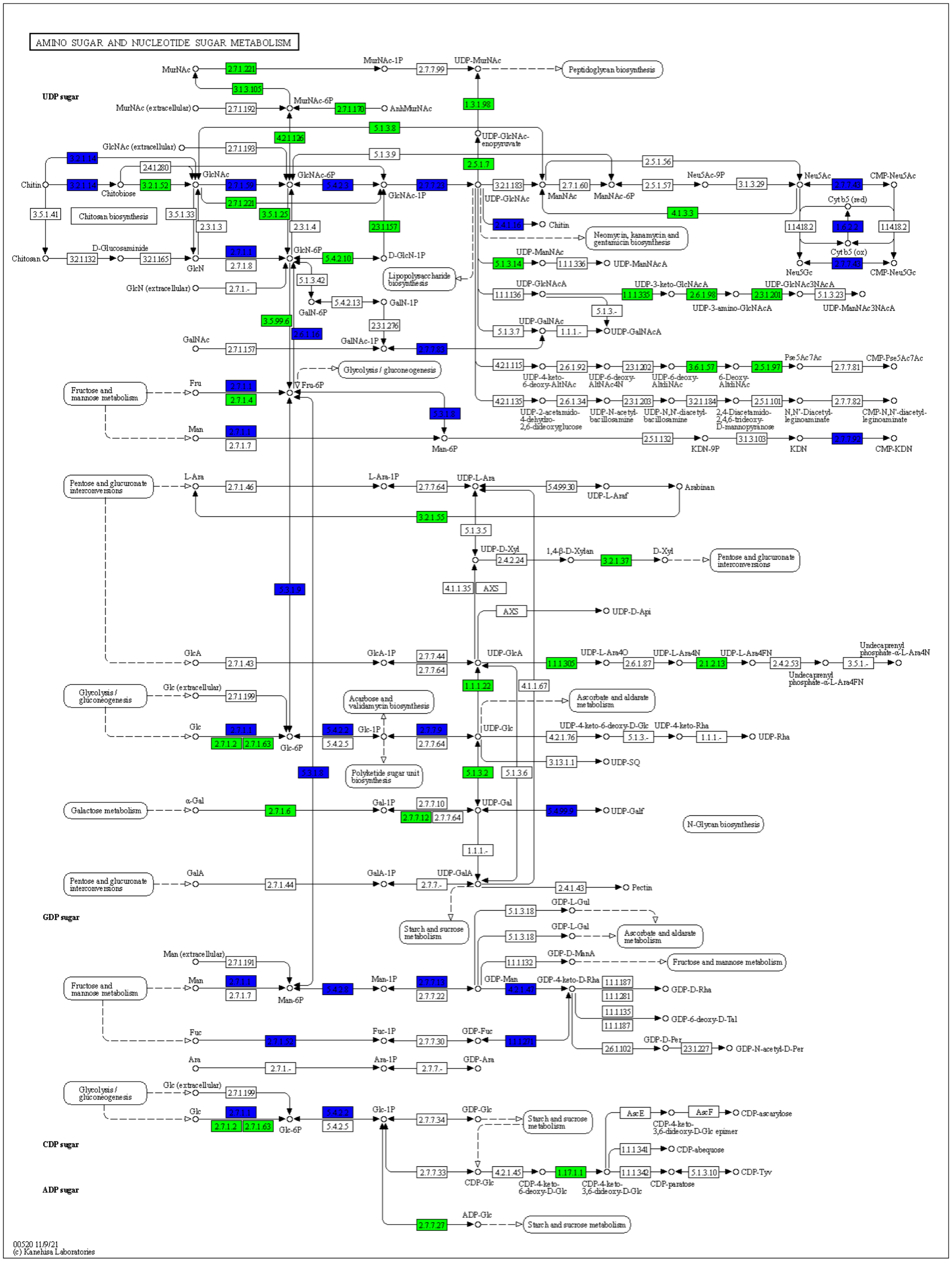

AMINO SUGAR AND NUCLEOTIDE SUGAR METABOLISM
UDP sugar
GDP sugar
CDP sugar
ADP sugar
MurNAc
MurNAc-1P
UDP-MurNAc
Peptidoglycan biosynthesis
MurNAc (extracellular)
MurNAc-6P
AnhMurNAc
GlcNAc (extracellular)
UDP-GlcNAc-enolpyruvate
Chitin
Chitobiose
GlcNAc
GlcNAc-6P
GlcNAc-1P
UDP-GlcNAc
ManNAc
ManNAc-6P
Neu5Ac-9P
Neu5Ac
CMP-Neu5Ac
Cyt b5 (red)
Cyt b5 (ox)
Neu5Gc
CMP-Neu5Gc
Chitosan biosynthesis
Chitosan
D-Glucosaminide
GlcN
GlcN-6P
D-GlcN-1P
Neomycin, kanamycin and gentamicin biosynthesis
Lipopolysaccharide biosynthesis
UDP-ManNAc
UDP-ManNAcA
UDP-GlcNAcA
UDP-3-keto-GlcNAcA
UDP-3-amino-GlcNAcA
UDP-GlcNAc3NAcA
UDP-ManNAc3NAcA
GlcN (extracellular)
GalN-6P
GalN-1P
UDP-GalNAc
UDP-GalNAcA
GalNAc
GalNAc-1P
Glycolysis / gluconeogenesis
Fructose and mannose metabolism
Fru
Fru-6P
Man
Man-6P
UDP-4-keto-6-deoxy-AltNAc
UDP-6-deoxy-AltNAc4N
UDP-6-deoxy-AltdiNAc
6-Deoxy-AltdiNAc
Pse5Ac7Ac
CMP-Pse5Ac7Ac
UDP-2-acetamido-4-dehydro-2,6-dideoxyglucose
UDP-N-acetylbacillosamine
UDP-N,N'-diacetylbacillosamine
2,4-Diacetamido-2,4,6-trideoxy-D-mannopyranose
N,N'-Diacetyllegionaminate
CMP-N,N'-diacetyllegionaminate
KDN-9P
KDN
CMP-KDN
Pentose and glucuronate interconversions
L-Ara
L-Ara-1P
UDP-L-Ara
UDP-L-Araf
Arabinan
UDP-D-Xyl
1,4-β-D-Xylan
D-Xyl
UDP-D-Api
GlcA
GlcA-1P
UDP-GlcA
UDP-L-Ara4O
UDP-L-Ara4N
UDP-L-Ara4FN
Undecaprenyl phosphate-α-L-Ara4N
Undecaprenyl phosphate-α-L-Ara4FN
Glc (extracellular)
Glc
Glc-6P
Glc-1P
Acarbose and validamycin biosynthesis
Polyketide sugar unit biosynthesis
UDP-Glc
UDP-4-keto-6-deoxy-D-Glc
UDP-4-keto-Rha
UDP-Rha
UDP-SQ
Ascorbate and aldarate metabolism
Galactose metabolism
α-Gal
Gal-1P
UDP-Gal
UDP-Galf
N-Glycan biosynthesis
GalA
GalA-1P
UDP-GalA
Pectin
Starch and sucrose metabolism
GDP-L-Gul
GDP-L-Gal
GDP-D-ManA
Man (extracellular)
Man-1P
GDP-Man
GDP-4-keto-D-Rha
GDP-D-Rha
GDP-6-deoxy-D-Tal
Fuc
Fuc-1P
GDP-Fuc
GDP-D-Per
GDP-N-acetyl-D-Per
Ara
Ara-1P
GDP-Ara
GDP-Glc
CDP-Glc
CDP-4-keto-6-deoxy-D-Glc
CDP-4-keto-3,6-dideoxy-D-Glc
CDP-4-keto-3,6-dideoxy-D-Glc epimer
CDP-ascarylose
CDP-abequose
CDP-paratose
CDP-Tyv
ADP-Glc
00520 11/9/21
(c) Kanehisa Laboratories

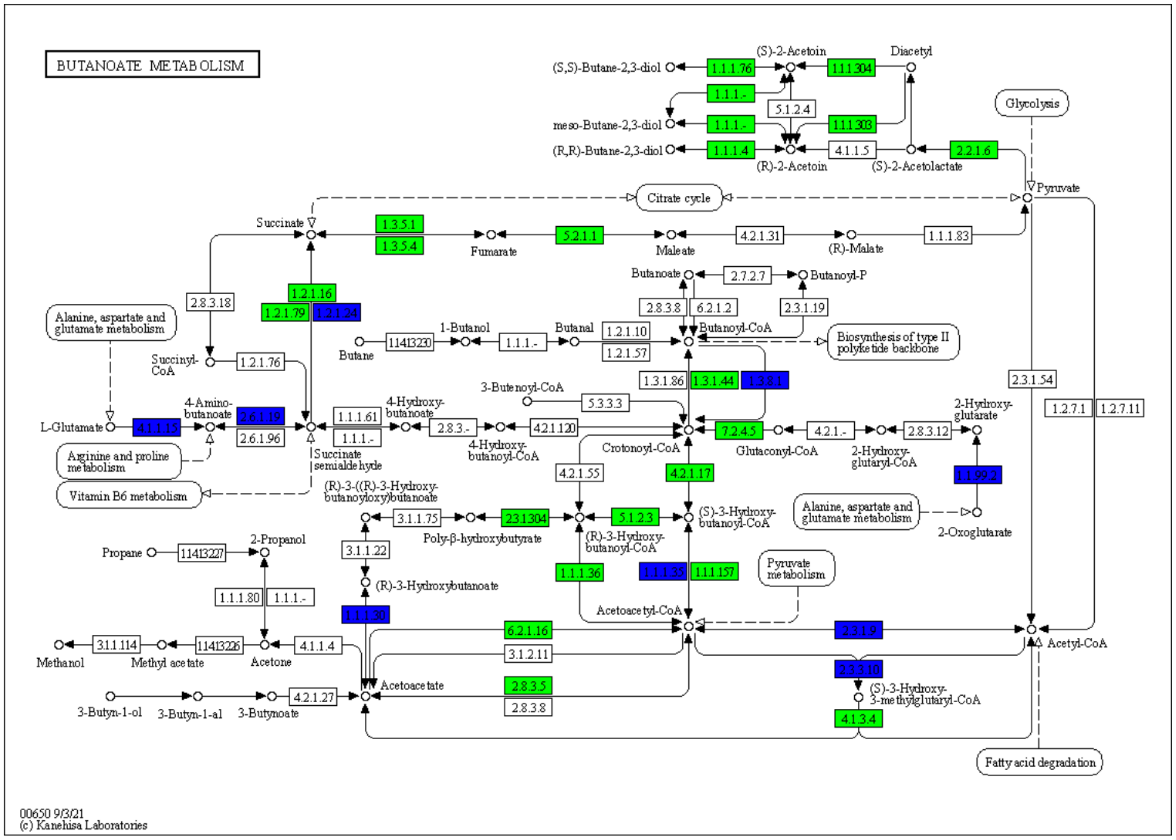

BUTANOATE METABOLISM
(S,S)-Butane-2,3-diol
1.1.1.76
(S)-2-Acetoin
1.1.1.304
Diacetyl
1.1.1.-
5.1.2.4
meso-Butane-2,3-diol
1.1.1.-
1.1.1.303
Glycolysis
(R,R)-Butane-2,3-diol
1.1.1.4
4.1.1.5
2.2.1.6
(R)-2-Acetoin
(S)-2-Acetolactate
Pyruvate
Citrate cycle
Succinate
1.3.5.1
1.3.5.4
5.2.1.1
4.2.1.31
1.1.1.83
Fumarate
Maleate
(R)-Malate
Butanoate
2.7.2.7
Butanoyl-P
1.2.1.16
2.8.3.18
1.2.1.79
1.2.1.24
2.8.3.8
6.2.1.2
2.3.1.19
Alanine, aspartate and glutamate metabolism
1-Butanol
Butanal
1.2.1.10
Butanoyl-CoA
Biosynthesis of type II polyketide backbone
Butane
11413230
1.1.1.-
1.2.1.57
Succinyl-CoA
1.2.1.76
3-Butenoyl-CoA
1.3.1.86
1.3.1.44
1.3.8.1
2.3.1.54
5.3.3.3
1.2.7.1
1.2.7.11
4-Amino-butanoate
4-Hydroxy-butanoate
2-Hydroxy-glutarate
L-Glutamate
4.1.1.15
2.6.1.19
1.1.1.61
2.8.3.-
4.2.1.120
7.2.4.5
4.2.1.-
2.8.3.12
2.6.1.96
1.1.1.-
4-Hydroxy-butanoyl-CoA
Crotonoyl-CoA
Glutaconyl-CoA
2-Hydroxy-glutaryl-CoA
Arginine and proline metabolism
Succinate semialdehyde
4.2.1.55
4.2.1.17
1.1.99.2
Vitamin B6 metabolism
(R)-3-((R)-3-Hydroxy-butanoyloxy)butanoate
Alanine, aspartate and glutamate metabolism
3.1.1.75
2.3.1.304
5.1.2.3
(S)-3-Hydroxy-butanoyl-CoA
2-Oxoglutarate
Poly-β-hydroxybutyrate
(R)-3-Hydroxy-butanoyl-CoA
Propane
11413227
2-Propanol
3.1.1.22
Pyruvate metabolism
1.1.1.36
1.1.1.35
1.1.1.157
(R)-3-Hydroxybutanoate
1.1.1.80
1.1.1.-
Acetoacetyl-CoA
1.1.1.30
6.2.1.16
2.3.1.9
Acetyl-CoA
Methanol
3.1.1.114
Methyl acetate
11413226
Acetone
4.1.1.4
3.1.2.11
2.3.3.10
(S)-3-Hydroxy-3-methylglutaryl-CoA
2.8.3.5
3-Butyn-1-ol
3-Butyn-1-al
3-Butynoate
4.2.1.27
Acetoacetate
2.8.3.8
4.1.3.4
Fatty acid degradation
00650 9/3/21
(c) Kanehisa Laboratories

Lipid Metabolism

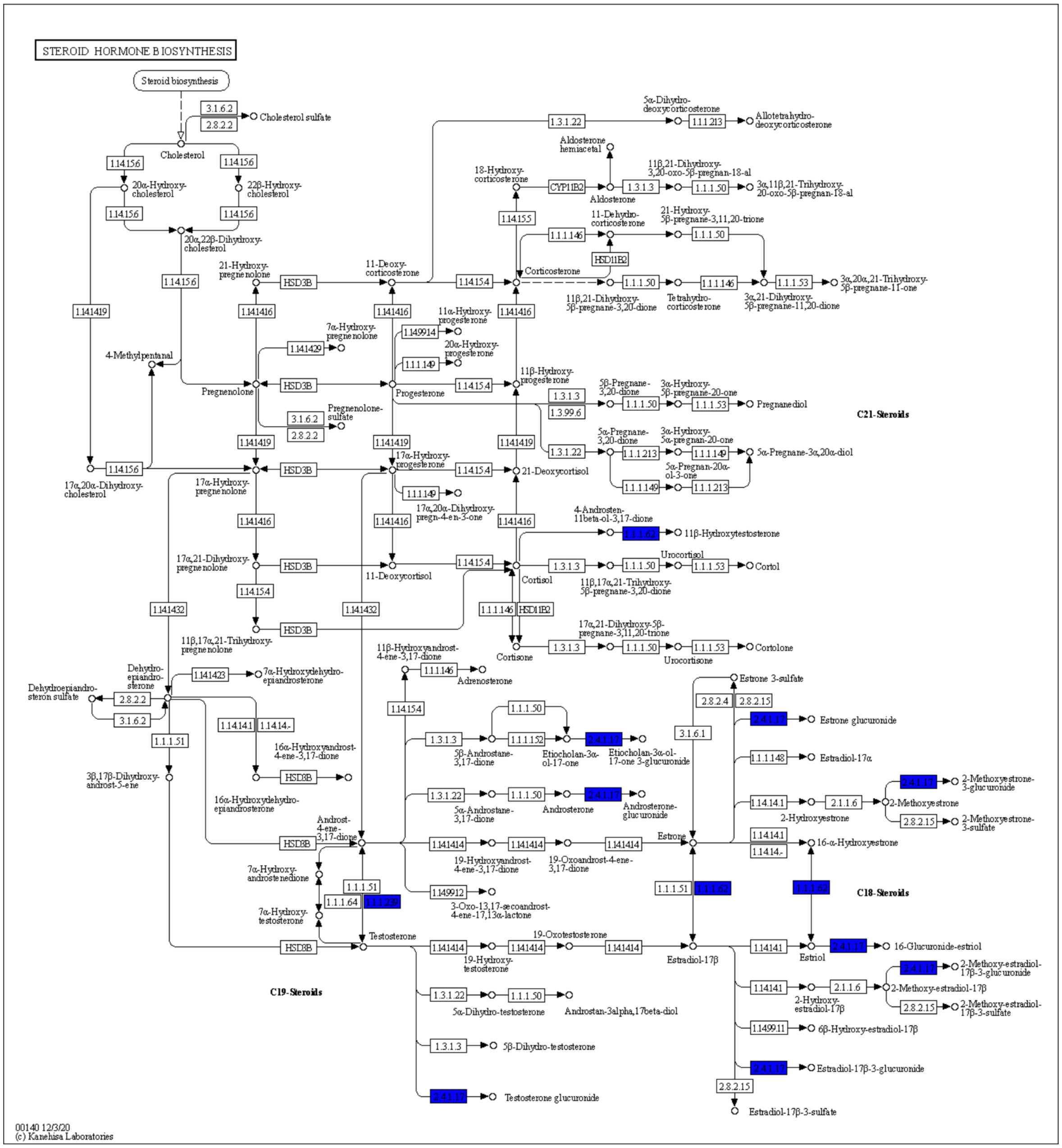

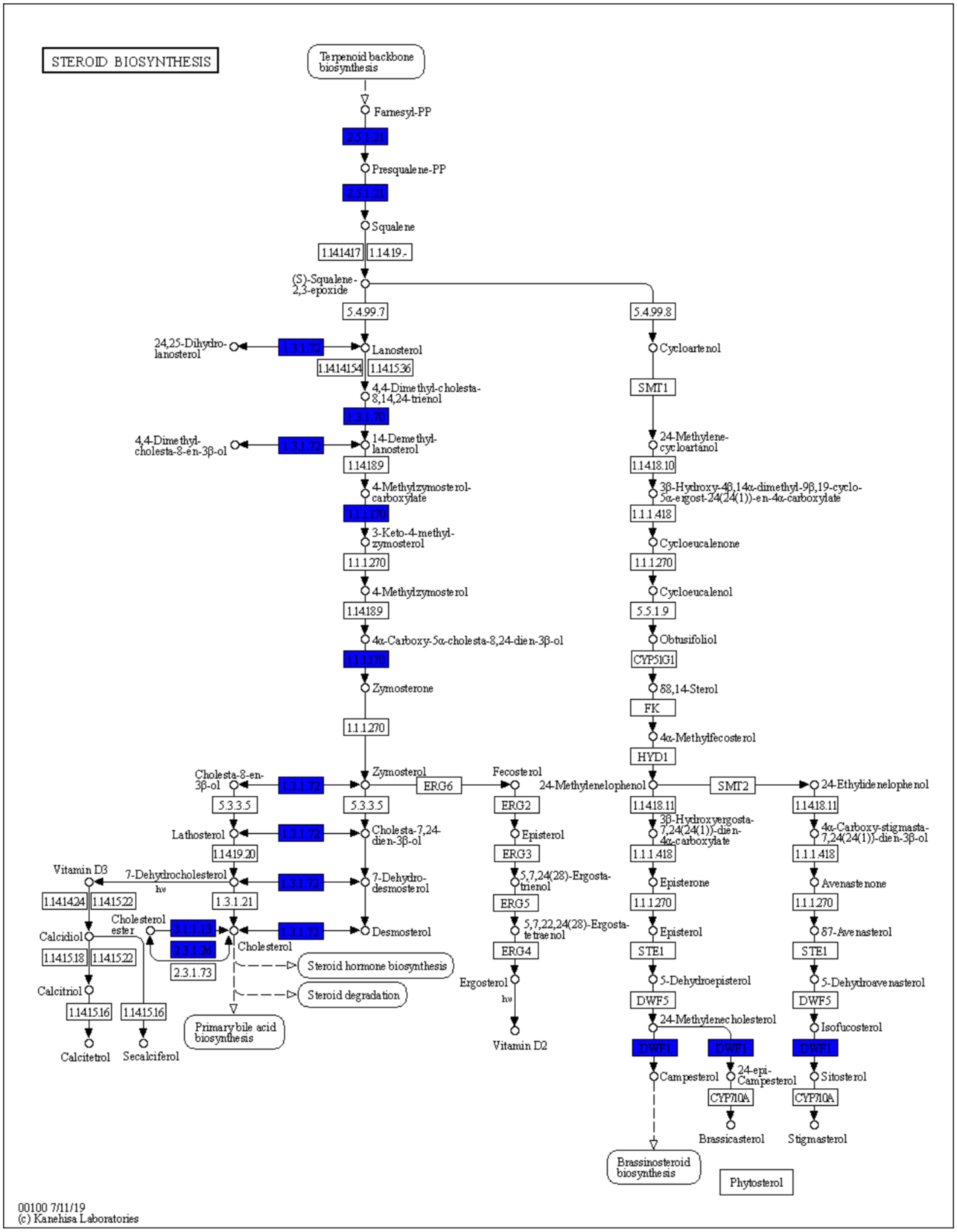
STEROID BIOSYNTHESIS
Terpenoid backbone biosynthesis
Farnesyl-PP
2.5.1.21
Presqualene-PP
2.5.1.21
Squalene
1.14.14.17
1.14.19.-
(S)-Squalene-2,3-epoxide
5.4.99.7
5.4.99.8
24,25-Dihydro-lanosterol
1.3.1.72
Lanosterol
1.14.14.154
1.14.15.36
4,4-Dimethyl-cholesta-8,14,24-trienol
1.3.1.70
4,4-Dimethyl-cholesta-8-en-3β-ol
1.3.1.72
14-Demethyl-lanosterol
1.14.18.9
4-Methylzymosterol-carboxylate
1.1.1.170
3-Keto-4-methyl-zymosterol
1.1.1.270
4-Methylzymosterol
1.14.18.9
4α-Carboxy-5α-cholesta-8,24-dien-3β-ol
1.1.1.170
Zymosterone
1.1.1.270
Cycloartenol
SMT1
24-Methylene-cycloartanol
1.14.18.10
3β-Hydroxy-4β,14α-dimethyl-9β,19-cyclo-5α-ergost-24(24(1))-en-4α-carboxylate
1.1.1.418
Cycloeucalenone
1.1.1.270
Cycloeucalenol
5.5.1.9
Obtusifoliol
CYP51G1
δ8,14-Sterol
FK
4α-Methylfecosterol
HYD1
Cholesta-8-en-3β-ol
1.3.1.72
Zymosterol
ERG6
Fecosterol
24-Methylenelophenol
SMT2
24-Ethylidenelophenol
5.3.3.5
5.3.3.5
ERG2
1.14.18.11
1.14.18.11
Lathosterol
1.3.1.72
Cholesta-7,24-dien-3β-ol
Episterol
3β-Hydroxyergosta-7,24(24(1))-dien-4α-carboxylate
4α-Carboxy-stigmasta-7,24(24(1))-dien-3β-ol
1.14.19.20
ERG3
1.1.1.418
1.1.1.418
Vitamin D3
7-Dehydrocholesterol
1.3.1.72
7-Dehydro-desmosterol
5,7,24(28)-Ergosta-trienol
Episterone
Avenastenone
hν
1.14.14.24
1.14.15.22
1.3.1.21
ERG5
1.1.1.270
1.1.1.270
Cholesterol ester
3.1.1.13
1.3.1.72
Desmosterol
5,7,22,24(28)-Ergosta-tetraenol
Episterol
δ7-Avenasterol
Calcidiol
2.3.1.26
Cholesterol
ERG4
STE1
STE1
1.14.15.18
1.14.15.22
2.3.1.73
Steroid hormone biosynthesis
Ergosterol
5-Dehydroepisterol
5-Dehydroavenasterol
Calcitriol
Steroid degradation
hν
DWF5
DWF5
1.14.15.16
1.14.15.16
24-Methylenecholesterol
Isofucosterol
Primary bile acid biosynthesis
Vitamin D2
DWF1
DWF1
DWF1
Calcitetrol
Secalciferol
Campesterol
24-epi-Campesterol
Sitosterol
CYP710A
CYP710A
Brassicasterol
Stigmasterol
Brassinosteroid biosynthesis
Phytosterol
00100 7/11/19
(c) Kanehisa Laboratories

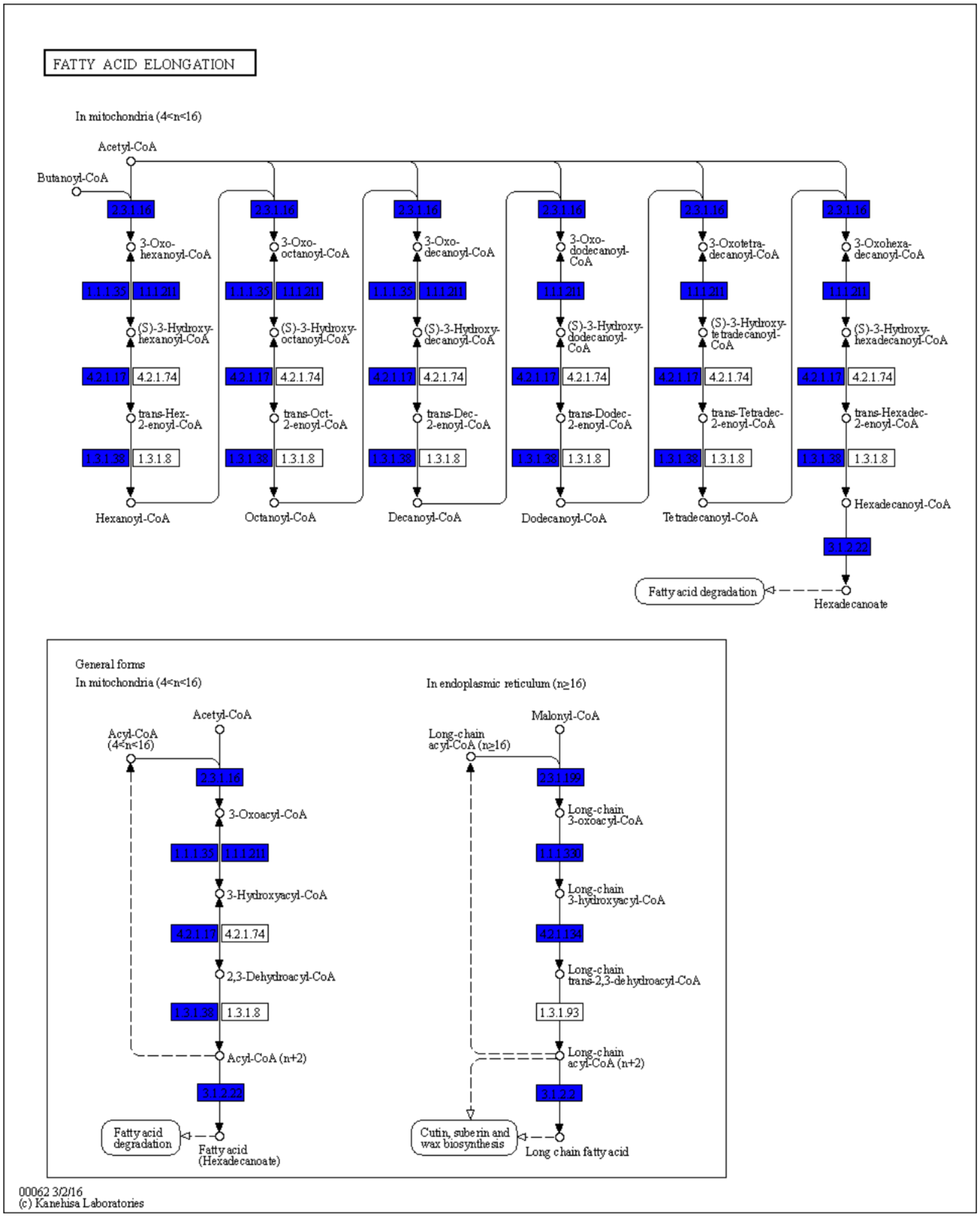
FATTY ACID ELONGATION
In mitochondria (4<n<16)
Acetyl-CoA
Butanoyl-CoA
2.3.1.16
3-Oxo-hexanoyl-CoA
3-Oxo-octanoyl-CoA
3-Oxo-decanoyl-CoA
3-Oxo-dodecanoyl-CoA
3-Oxotetra-decanoyl-CoA
3-Oxohexa-decanoyl-CoA
1.1.1.35
1.1.1.211
(S)-3-Hydroxy-hexanoyl-CoA
(S)-3-Hydroxy-octanoyl-CoA
(S)-3-Hydroxy-decanoyl-CoA
(S)-3-Hydroxy-dodecanoyl-CoA
(S)-3-Hydroxy-tetradecanoyl-CoA
(S)-3-Hydroxy-hexadecanoyl-CoA
4.2.1.17
4.2.1.74
trans-Hex-2-enoyl-CoA
trans-Oct-2-enoyl-CoA
trans-Dec-2-enoyl-CoA
trans-Dodec-2-enoyl-CoA
trans-Tetradec-2-enoyl-CoA
trans-Hexadec-2-enoyl-CoA
1.3.1.38
1.3.1.8
Hexanoyl-CoA
Octanoyl-CoA
Decanoyl-CoA
Dodecanoyl-CoA
Tetradecanoyl-CoA
Hexadecanoyl-CoA
3.1.2.22
Fatty acid degradation
Hexadecanoate
General forms
In mitochondria (4<n<16)
Acetyl-CoA
Acyl-CoA (4<n<16)
2.3.1.16
3-Oxoacyl-CoA
1.1.1.35
1.1.1.211
3-Hydroxyacyl-CoA
4.2.1.17
4.2.1.74
2,3-Dehydroacyl-CoA
1.3.1.38
1.3.1.8
Acyl-CoA (n+2)
3.1.2.22
Fatty acid degradation
Fatty acid (Hexadecanoate)
In endoplasmic reticulum (n≥16)
Malonyl-CoA
Long-chain acyl-CoA (n≥16)
2.3.1.199
Long-chain 3-oxoacyl-CoA
1.1.1.330
Long-chain 3-hydroxyacyl-CoA
4.2.1.134
Long-chain trans-2,3-dehydroacyl-CoA
1.3.1.93
Long-chain acyl-CoA (n+2)
3.1.2.2
Cutin, suberin and wax biosynthesis
Long chain fatty acid
00062 3/2/16
(c) Kanehisa Laboratories

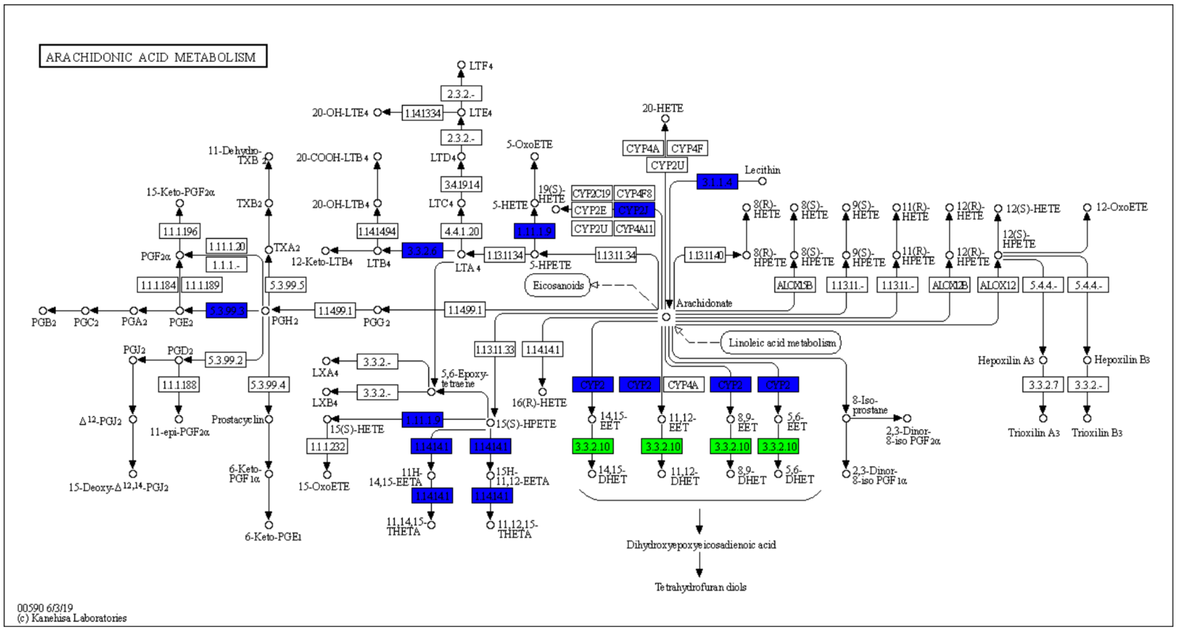
ARACHIDONIC ACID METABOLISM
LTF4
2.3.2.-
20-OH-LTE4
1.14.13.34
LTE4
2.3.2.-
LTD4
20-COOH-LTB4
3.4.19.14
5-OxoETE
20-HETE
CYP4A
CYP4F
CYP2U
Lecithin
11-Dehydro-TXB2
15-Keto-PGF2α
TXB2
20-OH-LTB4
LTC4
5-HETE
19(S)-HETE
CYP2C19
CYP4F8
CYP2E
CYP2U
CYP4A11
1.1.1.196
1.1.1.20
1.1.1.-
1.14.14.94
4.4.1.20
PGF2α
TXA2
12-Keto-LTB4
LTB4
LTA4
1.13.11.34
5-HPETE
1.13.11.34
1.13.11.40
8(R)-HETE
8(S)-HETE
9(S)-HETE
11(R)-HETE
12(R)-HETE
12(S)-HETE
12-OxoETE
8(R)-HPETE
8(S)-HPETE
9(S)-HPETE
11(R)-HPETE
12(R)-HPETE
12(S)-HPETE
1.1.1.184
1.1.1.189
5.3.99.5
Eicosanoids
ALOX15B
1.13.11.-
1.13.11.-
ALOX12B
ALOX12
5.4.4.-
5.4.4.-
Arachidonate
PGB2
PGC2
PGA2
PGE2
PGH2
1.14.99.1
PGG2
1.14.99.1
1.13.11.33
1.14.14.1
Linoleic acid metabolism
PGJ2
PGD2
5.3.99.2
LXA4
3.3.2.-
5,6-Epoxy-tetraene
Hepoxilin A3
Hepoxilin B3
1.1.1.188
5.3.99.4
LXB4
3.3.2.-
16(R)-HETE
CYP4A
8-Iso-prostane
3.3.2.7
3.3.2.-
Δ12-PGJ2
11-epi-PGF2α
Prostacyclin
15(S)-HETE
15(S)-HPETE
14,15-EET
11,12-EET
8,9-EET
5,6-EET
2,3-Dinor-8-iso PGF2α
Trioxilin A3
Trioxilin B3
1.1.1.232
3.3.2.10
3.3.2.10
3.3.2.10
3.3.2.10
6-Keto-PGF1α
11H-14,15-EETA
15H-11,12-EETA
14,15-DHET
11,12-DHET
8,9-DHET
5,6-DHET
2,3-Dinor-8-iso PGF1α
15-Deoxy-Δ12,14-PGJ2
15-OxoETE
11,14,15-THETA
11,12,15-THETA
6-Keto-PGE1
Dihydroxyepoxyeicosadienoic acid
Tetrahydrofuran diols
00590 6/3/19
(c) Kanehisa Laboratories

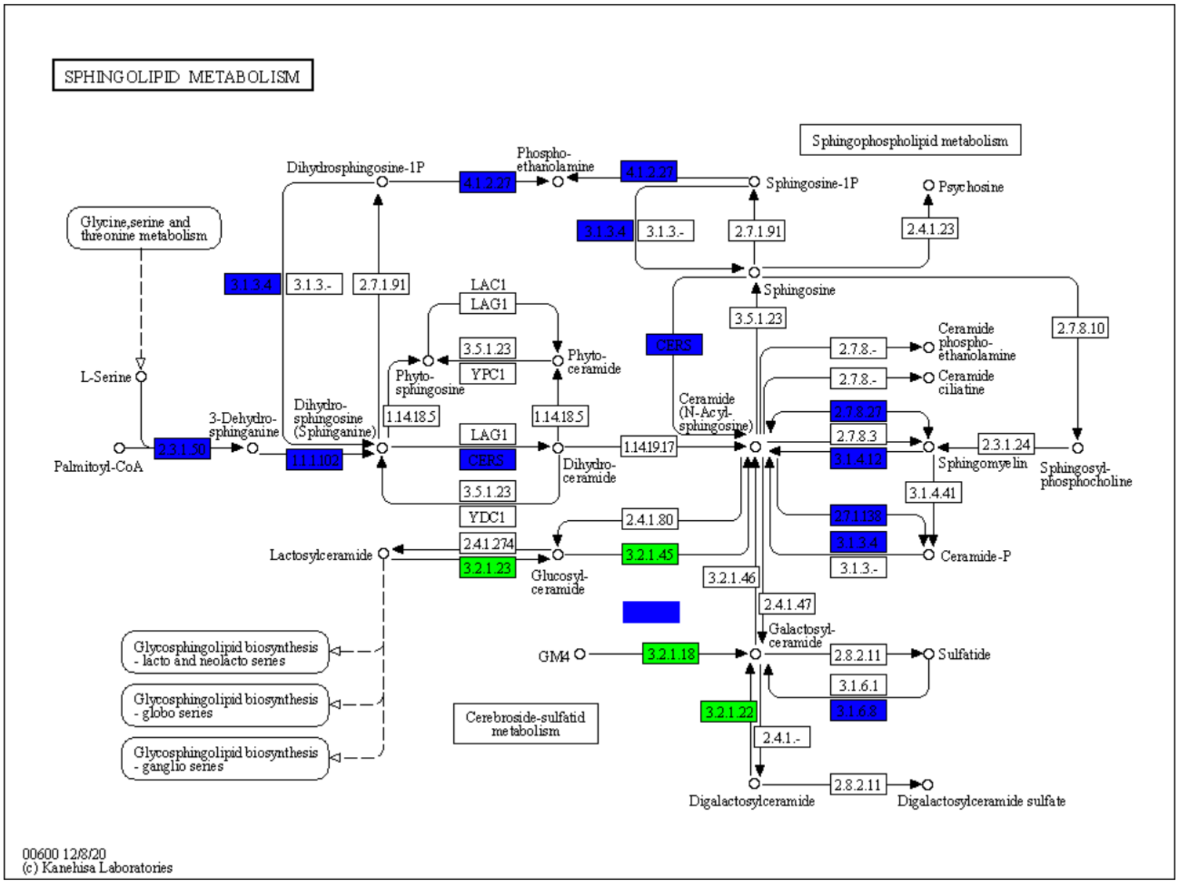
SPHINGOLIPID METABOLISM
Sphingophospholipid metabolism
Phospho-ethanolamine
Dihydrosphingosine-1P
4.1.2.27
4.1.2.27
Sphingosine-1P
Psychosine
Glycine, serine and threonine metabolism
3.1.3.4
3.1.3.-
2.7.1.91
2.4.1.23
3.1.3.4
3.1.3.-
2.7.1.91
Sphingosine
LAC1
LAG1
3.5.1.23
CERS
2.7.8.10
3.5.1.23
Phyto-ceramide
2.7.8.-
Ceramide phospho-ethanolamine
L-Serine
YPC1
Phyto-sphingosine
2.7.8.-
Ceramide ciliatine
Dihydro-sphingosine (Sphinganine)
1.14.18.5
1.14.18.5
Ceramide (N-Acyl-sphingosine)
2.7.8.27
3-Dehydro-sphinganine
LAG1
1.14.19.17
2.7.8.3
2.3.1.24
Palmitoyl-CoA
2.3.1.50
1.1.1.102
CERS
Dihydro-ceramide
3.1.4.12
Sphingomyelin
Sphingosyl-phosphocholine
3.5.1.23
3.1.4.41
YDC1
2.4.1.80
2.7.1.138
2.4.1.274
3.1.3.4
Lactosylceramide
3.2.1.23
3.2.1.45
Ceramide-P
Glucosyl-ceramide
3.1.3.-
3.2.1.46
2.4.1.47
Glycosphingolipid biosynthesis - lacto and neolacto series
GM4
3.2.1.18
Galactosyl-ceramide
2.8.2.11
Sulfatide
Glycosphingolipid biosynthesis - globo series
3.1.6.1
Cerebroside-sulfatide metabolism
3.2.1.22
3.1.6.8
Glycosphingolipid biosynthesis - ganglio series
2.4.1.-
2.8.2.11
Digalactosylceramide
Digalactosylceramide sulfate
00600 12/8/20
(c) Kanehisa Laboratories

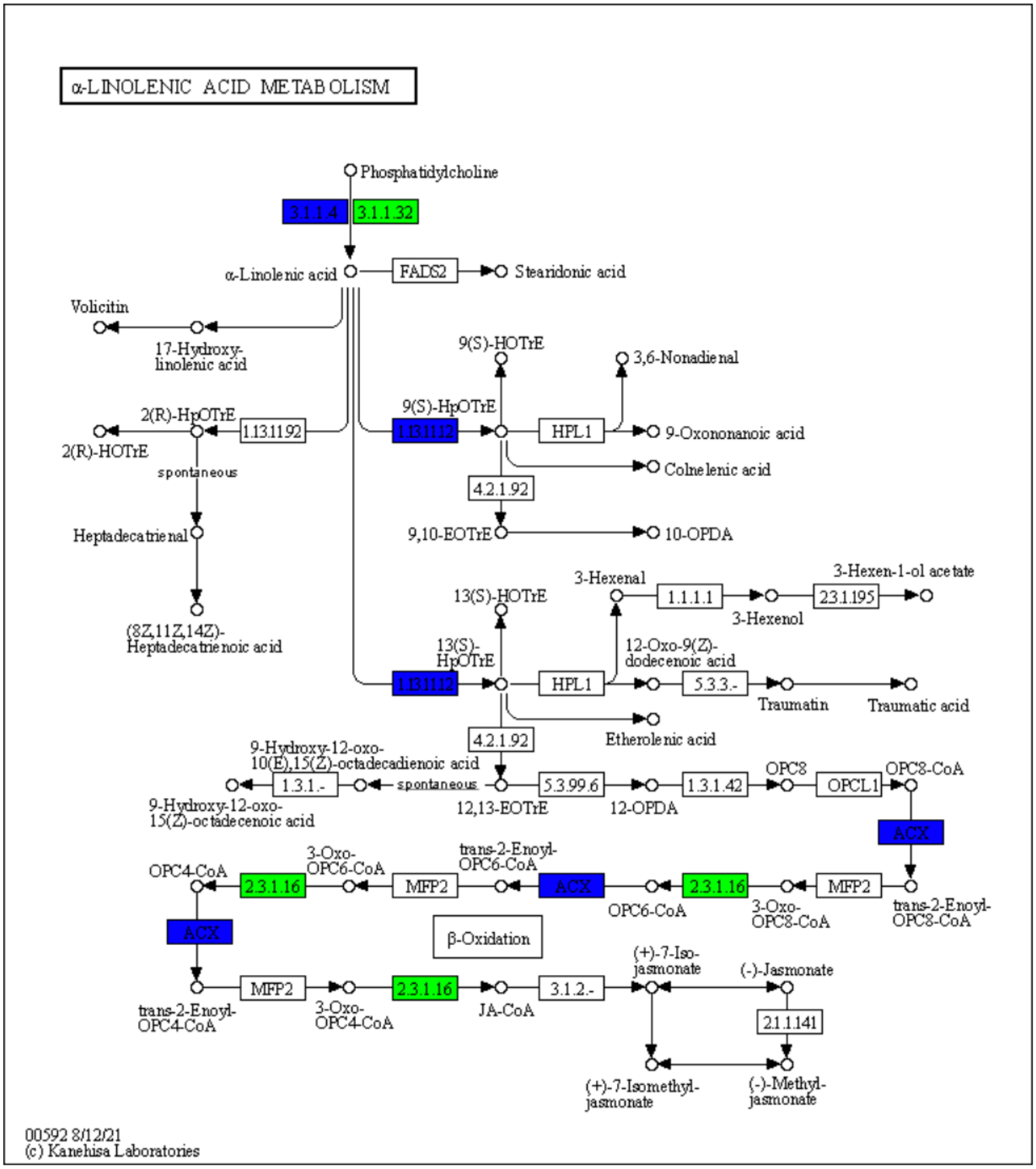

α-LINOLENIC ACID METABOLISM
Phosphatidylcholine
3.1.1.4
3.1.1.32
α-Linolenic acid
FADS2
Stearidonic acid
Volicitin
17-Hydroxy-linolenic acid
9(S)-HOTrE
3,6-Nonadienal
2(R)-HpOTrE
1.13.11.92
2(R)-HOTrE
9(S)-HpOTrE
1.13.11.12
HPL1
9-Oxononanoic acid
Colnelenic acid
spontaneous
4.2.1.92
Heptadecatrienal
9,10-EOTrE
10-OPDA
(8Z,11Z,14Z)-Heptadecatrienoic acid
13(S)-HOTrE
3-Hexenal
1.1.1.1
3-Hexenol
3-Hexen-1-ol acetate
2.3.1.195
13(S)-HpOTrE
12-Oxo-9(Z)-dodecenoic acid
5.3.3.-
Traumatin
Traumatic acid
Etherolenic acid
9-Hydroxy-12-oxo-10(E),15(Z)-octadecadienoic acid
1.3.1.-
9-Hydroxy-12-oxo-15(Z)-octadecenoic acid
12,13-EOTrE
5.3.99.6
12-OPDA
1.3.1.42
OPC8
OPCL1
OPC8-CoA
ACX
trans-2-Enoyl-OPC8-CoA
MFP2
3-Oxo-OPC8-CoA
2.3.1.16
OPC6-CoA
trans-2-Enoyl-OPC6-CoA
3-Oxo-OPC6-CoA
OPC4-CoA
β-Oxidation
trans-2-Enoyl-OPC4-CoA
3-Oxo-OPC4-CoA
JA-CoA
3.1.2.-
(+)-7-Iso-jasmonate
(-)-Jasmonate
2.1.1.141
(+)-7-Isomethyl-jasmonate
(-)-Methyl-jasmonate
00592 8/12/21
(c) Kanehisa Laboratories

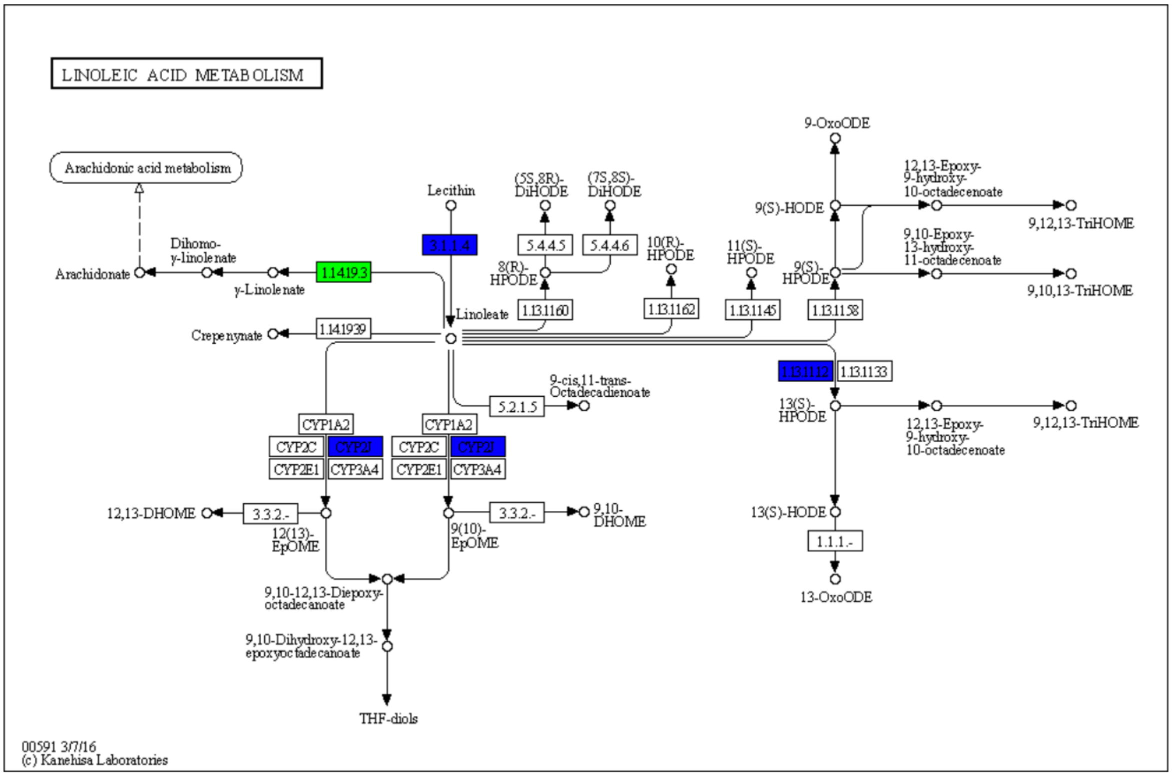

LINOLEIC ACID METABOLISM
Arachidonic acid metabolism
Arachidonate
Dihomo-γ-linolenate
γ-Linolenate
1.14.19.3
Lecithin
Linoleate
Crepenynate
1.14.19.39
(5S,8R)-DiHODE
(7S,8S)-DiHODE
5.4.4.5
5.4.4.6
8(R)-HPODE
1.13.11.60
10(R)-HPODE
1.13.11.62
11(S)-HPODE
1.13.11.45
9-OxoODE
9(S)-HODE
9(S)-HPODE
1.13.11.58
12,13-Epoxy-9-hydroxy-10-octadecenoate
9,12,13-TriHOME
9,10-Epoxy-13-hydroxy-11-octadecenoate
9,10,13-TriHOME
1.13.11.33
13(S)-HPODE
12,13-Epoxy-9-hydroxy-10-octadecenoate
9,12,13-TriHOME
13(S)-HODE
1.1.1.-
13-OxoODE
9-cis,11-trans-Octadecadienoate
5.2.1.5
CYP1A2
CYP2C
CYP2E1
CYP3A4
CYP1A2
CYP2C
CYP2E1
CYP3A4
12,13-DHOME
3.3.2.-
12(13)-EpOME
9(10)-EpOME
3.3.2.-
9,10-DHOME
9,10-12,13-Diepoxy-octadecanoate
9,10-Dihydroxy-12,13-epoxyoctadecanoate
THF-diols
00591 3/7/16
(c) Kanehisa Laboratories

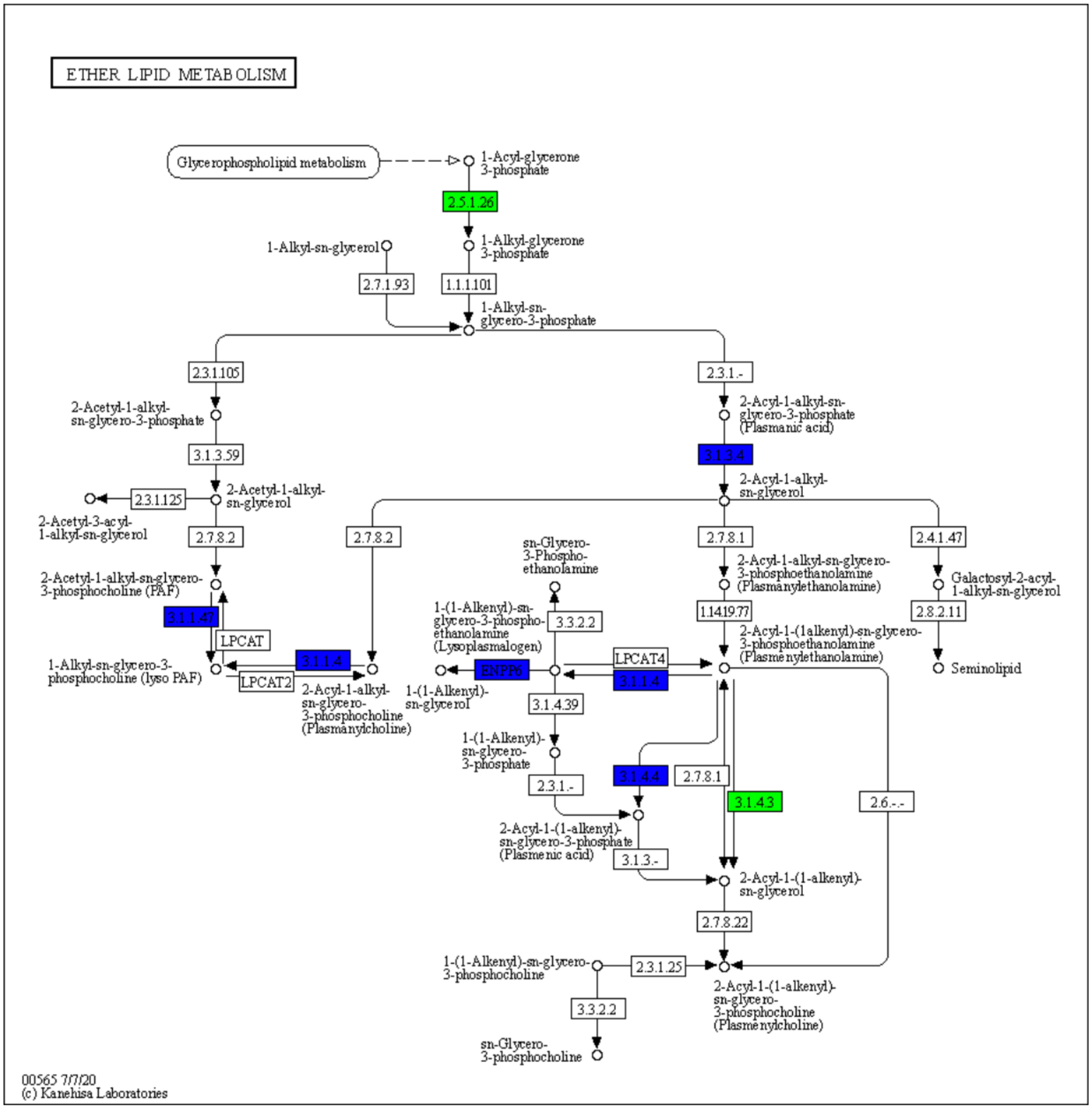
ETHER LIPID METABOLISM
Glycerophospholipid metabolism
1-Acyl-glycerone 3-phosphate
2.5.1.26
1-Alkyl-glycerone 3-phosphate
1-Alkyl-sn-glycerol
2.7.1.93
1.1.1.101
1-Alkyl-sn-glycero-3-phosphate
2.3.1.105
2.3.1.-
2-Acetyl-1-alkyl-sn-glycero-3-phosphate
2-Acyl-1-alkyl-sn-glycero-3-phosphate (Plasmanic acid)
3.1.3.59
3.1.3.4
2.3.1.125
2-Acetyl-1-alkyl-sn-glycerol
2-Acyl-1-alkyl-sn-glycerol
2-Acetyl-3-acyl-1-alkyl-sn-glycerol
2.7.8.2
2.7.8.2
2.7.8.1
2.4.1.47
sn-Glycero-3-Phospho-ethanolamine
2-Acyl-1-alkyl-sn-glycero-3-phosphoethanolamine (Plasmanylethanolamine)
Galactosyl-2-acyl-1-alkyl-sn-glycerol
2-Acetyl-1-alkyl-sn-glycero-3-phosphocholine (PAF)
3.1.1.47
LPCAT
1-(1-Alkenyl)-sn-glycero-3-phospho-ethanolamine (Lysoplasmalogen)
3.3.2.2
1.14.19.77
2.8.2.11
LPCAT4
2-Acyl-1-(1alkenyl)-sn-glycero-3-phosphoethanolamine (Plasmenylethanolamine)
1-Alkyl-sn-glycero-3-phosphocholine (lyso PAF)
3.1.1.4
LPCAT2
2-Acyl-1-alkyl-sn-glycero-3-phosphocholine (Plasmanylcholine)
ENPP6
1-(1-Alkenyl)-sn-glycerol
3.1.1.4
Seminolipid
3.1.4.39
1-(1-Alkenyl)-sn-glycero-3-phosphate
3.1.4.4
2.7.8.1
3.1.4.3
2.6.-.-
2.3.1.-
2-Acyl-1-(1-alkenyl)-sn-glycero-3-phosphate (Plasmenic acid)
3.1.3.-
2-Acyl-1-(1-alkenyl)-sn-glycerol
2.7.8.22
1-(1-Alkenyl)-sn-glycero-3-phosphocholine
2.3.1.25
2-Acyl-1-(1-alkenyl)-sn-glycero-3-phosphocholine (Plasmenylcholine)
3.3.2.2
sn-Glycero-3-phosphocholine
00565 7/7/20
(c) Kanehisa Laboratories

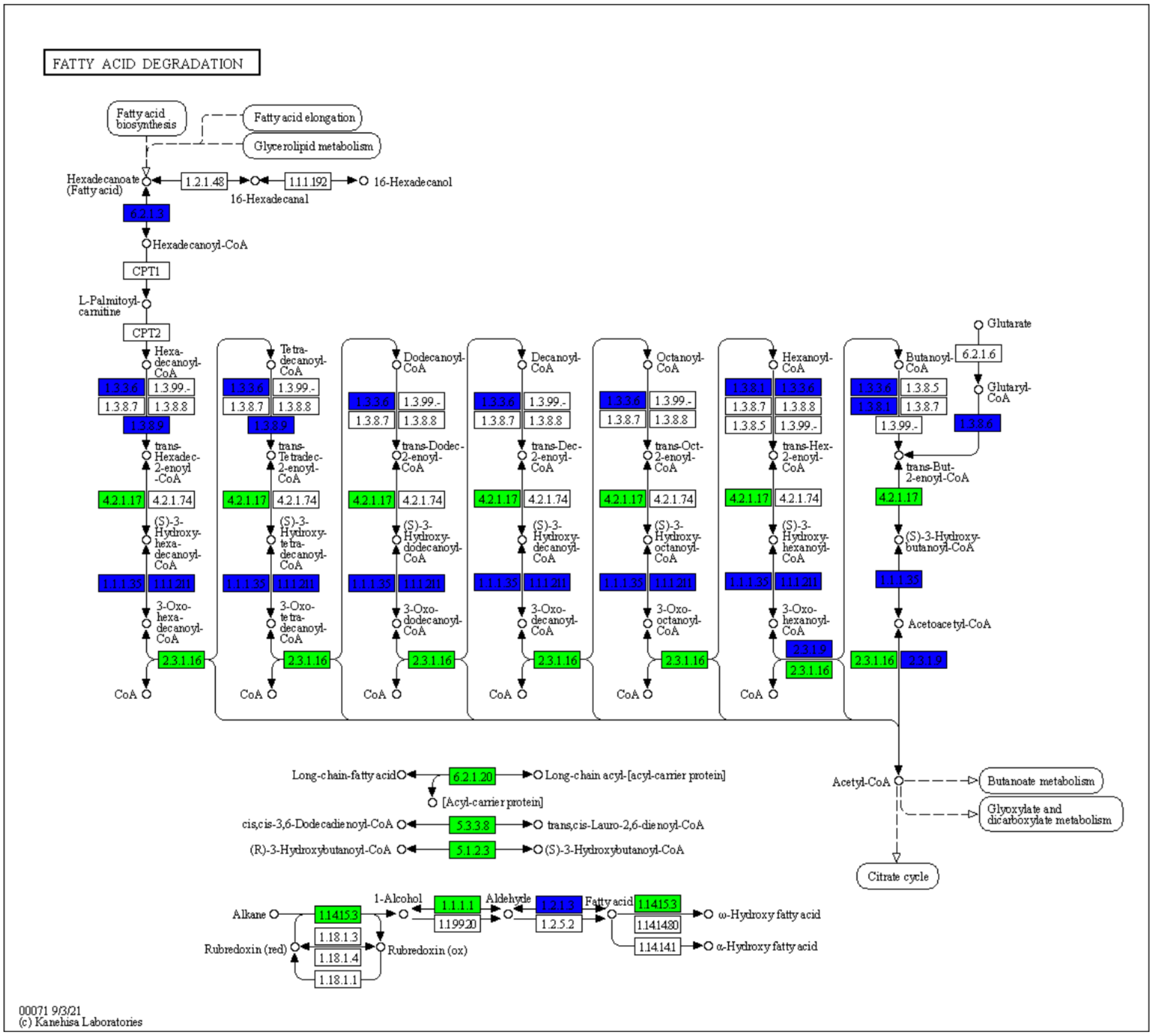
FATTY ACID DEGRADATION
Fatty acid biosynthesis
Fatty acid elongation
Glycerolipid metabolism
Hexadecanoate (Fatty acid)
1.2.1.48
16-Hexadecanal
1.1.1.192
16-Hexadecanol
6.2.1.3
Hexadecanoyl-CoA
CPT1
L-Palmitoyl-carnitine
CPT2
Hexa-decanoyl-CoA
Tetra-decanoyl-CoA
Dodecanoyl-CoA
Decanoyl-CoA
Octanoyl-CoA
Hexanoyl-CoA
Butanoyl-CoA
Glutarate
6.2.1.6
Glutaryl-CoA
1.3.3.6
1.3.99.-
1.3.8.7
1.3.8.8
1.3.8.9
1.3.8.1
1.3.8.5
1.3.8.6
trans-Hexadec-2-enoyl-CoA
trans-Tetradec-2-enoyl-CoA
trans-Dodec-2-enoyl-CoA
trans-Dec-2-enoyl-CoA
trans-Oct-2-enoyl-CoA
trans-Hex-2-enoyl-CoA
trans-But-2-enoyl-CoA
4.2.1.17
4.2.1.74
(S)-3-Hydroxy-hexa-decanoyl-CoA
(S)-3-Hydroxy-tetra-decanoyl-CoA
(S)-3-Hydroxy-dodecanoyl-CoA
(S)-3-Hydroxy-decanoyl-CoA
(S)-3-Hydroxy-octanoyl-CoA
(S)-3-Hydroxy-hexanoyl-CoA
(S)-3-Hydroxy-butanoyl-CoA
1.1.1.35
1.1.1.211
3-Oxo-hexa-decanoyl-CoA
3-Oxo-tetra-decanoyl-CoA
3-Oxo-dodecanoyl-CoA
3-Oxo-decanoyl-CoA
3-Oxo-octanoyl-CoA
3-Oxo-hexanoyl-CoA
Acetoacetyl-CoA
2.3.1.16
2.3.1.9
CoA
Acetyl-CoA
Butanoate metabolism
Glyoxylate and dicarboxylate metabolism
Citrate cycle
Long-chain-fatty acid
6.2.1.20
Long-chain acyl-[acyl-carrier protein]
[Acyl-carrier protein]
cis,cis-3,6-Dodecadienoyl-CoA
5.3.3.8
trans,cis-Lauro-2,6-dienoyl-CoA
(R)-3-Hydroxybutanoyl-CoA
5.1.2.3
(S)-3-Hydroxybutanoyl-CoA
Alkane
1.14.15.3
1-Alcohol
1.1.1.1
1.1.99.20
Aldehyde
1.2.1.3
1.2.5.2
Fatty acid
1.14.14.80
1.14.14.1
ω-Hydroxy fatty acid
α-Hydroxy fatty acid
1.18.1.3
1.18.1.4
1.18.1.1
Rubredoxin (red)
Rubredoxin (ox)
00071 9/3/21
(c) Kanehisa Laboratories

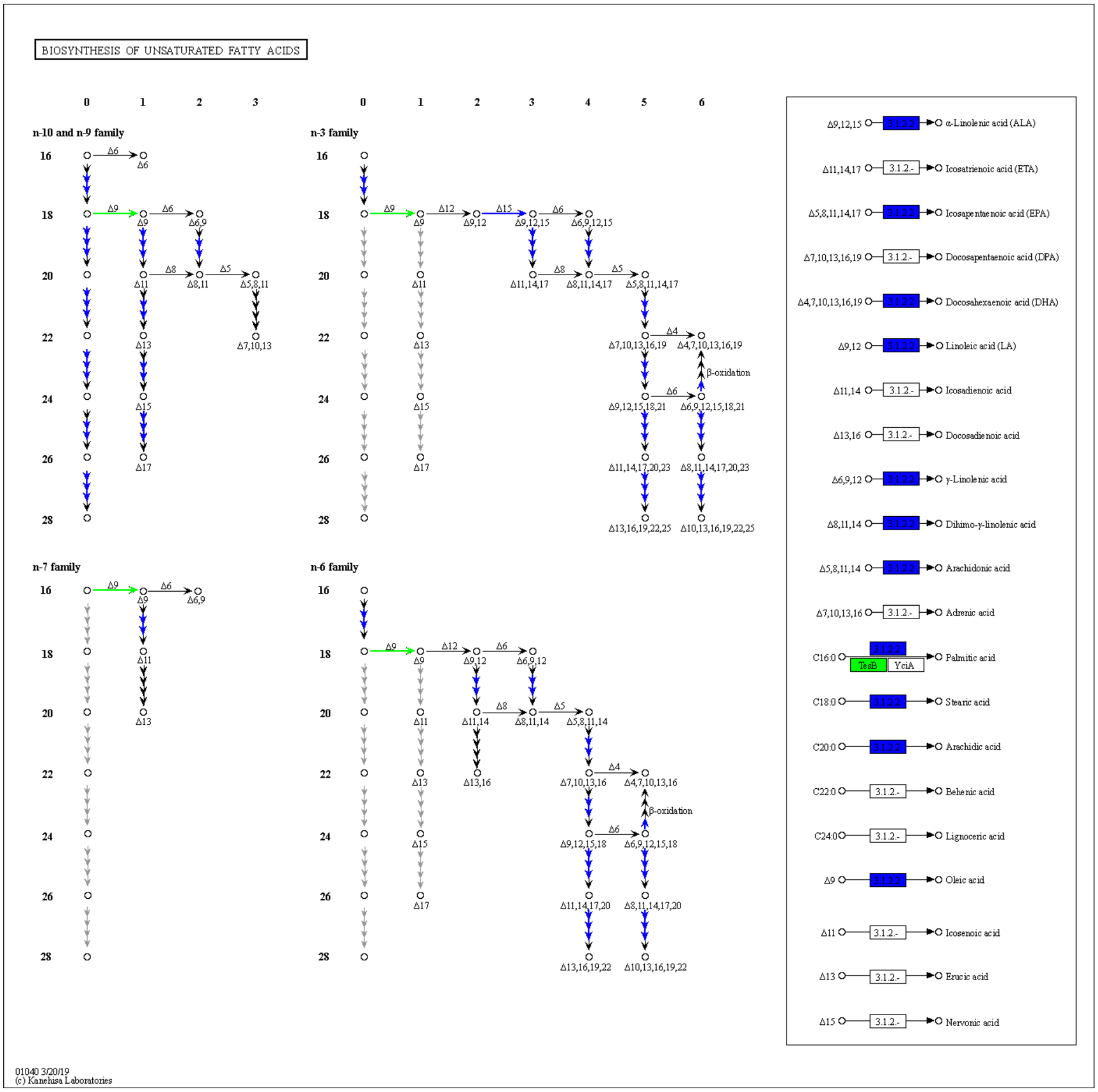
BIOSYNTHESIS OF UNSATURATED FATTY ACIDS
n-10 and n-9 family
n-3 family
n-7 family
n-6 family
β-oxidation
Δ9,12,15 3.1.2.- α-Linolenic acid (ALA)
Δ11,14,17 3.1.2.- Icosatrienoic acid (ETA)
Δ5,8,11,14,17 3.1.2.- Icosapentaenoic acid (EPA)
Δ7,10,13,16,19 3.1.2.- Docosapentaenoic acid (DPA)
Δ4,7,10,13,16,19 3.1.2.- Docosahexaenoic acid (DHA)
Δ9,12 3.1.2.- Linoleic acid (LA)
Δ11,14 3.1.2.- Icosadienoic acid
Δ13,16 3.1.2.- Docosadienoic acid
Δ6,9,12 3.1.2.- γ-Linolenic acid
Δ8,11,14 3.1.2.- Dihimo-γ-linolenic acid
Δ5,8,11,14 3.1.2.- Arachidonic acid
Δ7,10,13,16 3.1.2.- Adrenic acid
C16:0 3.1.2.- TesB YciA Palmitic acid
C18:0 3.1.2.- Stearic acid
C20:0 3.1.2.- Arachidic acid
C22:0 3.1.2.- Behenic acid
C24:0 3.1.2.- Lignoceric acid
Δ9 3.1.2.- Oleic acid
Δ11 3.1.2.- Icosenoic acid
Δ13 3.1.2.- Erucic acid
Δ15 3.1.2.- Nervonic acid
01040 3/20/19
(c) Kanehisa Laboratories

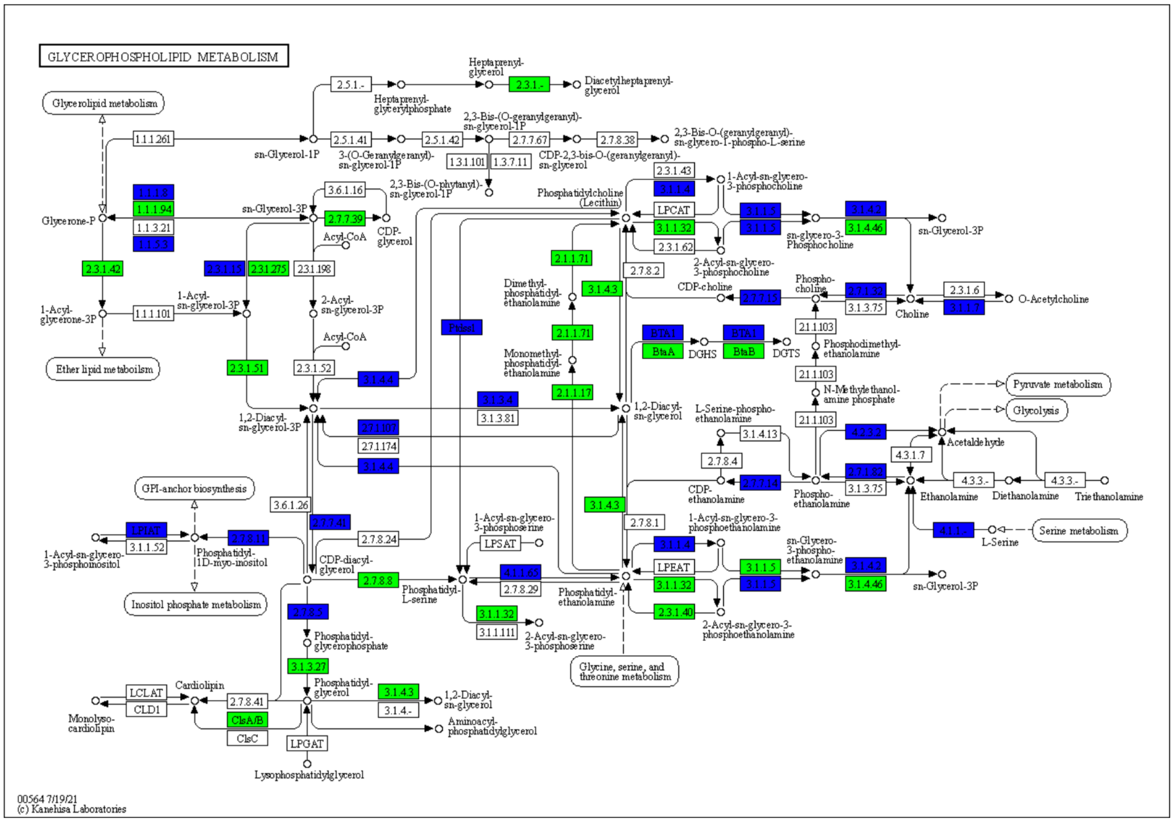
GLYCEROPHOSPHOLIPID METABOLISM
Glycerolipid metabolism
Glycerone-P
1-Acyl-glycerone-3P
Ether lipid metaboilsm
sn-Glycerol-1P
sn-Glycerol-3P
1-Acyl-sn-glycerol-3P
2-Acyl-sn-glycerol-3P
1,2-Diacyl-sn-glycerol-3P
Heptaprenyl-glycerol
Heptaprenyl-glycerylphosphate
Diacetylheptaprenyl-glycerol
3-(O-Geranylgeranyl)-sn-glycerol-1P
2,3-Bis-(O-geranylgeranyl)-sn-glycerol-1P
2,3-Bis-(O-phytanyl)-sn-glycerol-1P
CDP-2,3-bis-O-(geranylgeranyl)-sn-glycerol
2,3-Bis-O-(geranylgeranyl)-sn-glycero-1-phospho-L-serine
CDP-glycerol
Acyl-CoA
Phosphatidylcholine (Lecithin)
1-Acyl-sn-glycero-3-phosphocholine
2-Acyl-sn-glycero-3-phosphocholine
sn-glycero-3-Phosphocholine
sn-Glycerol-3P
CDP-choline
Phospho-choline
Choline
O-Acetylcholine
Dimethyl-phosphatidyl-ethanolamine
Monomethyl-phosphatidyl-ethanolamine
DGHS
DGTS
Phosphodimethyl-ethanolamine
N-Methylethanol-amine phosphate
1,2-Diacyl-sn-glycerol
L-Serine-phospho-ethanolamine
Pyruvate metabolism
Glycolysis
Acetaldehyde
CDP-ethanolamine
Phospho-ethanolamine
Ethanolamine
Diethanolamine
Triethanolamine
L-Serine
Serine metabolism
GPI-anchor biosynthesis
1-Acyl-sn-glycero-3-phosphoinositol
Phosphatidyl-1D-myo-inositol
Inositol phosphate metabolism
CDP-diacyl-glycerol
1-Acyl-sn-glycero-3-phosphoserine
Phosphatidyl-L-serine
2-Acyl-sn-glycero-3-phosphoserine
Phosphatidyl-ethanolamine
1-Acyl-sn-glycero-3-phosphoethanolamine
2-Acyl-sn-glycero-3-phosphoethanolamine
sn-Glycero-3-phospho-ethanolamine
Glycine, serine, and threonine metabolism
Phosphatidyl-glycerophosphate
Phosphatidyl-glycerol
Aminoacyl-phosphatidylglycerol
Lysophosphatidylglycerol
Cardiolipin
Monolyso-cardiolipin
LCLAT
CLD1
ClsA/B
ClsC
LPGAT
LPIAT
LPCAT
LPSAT
LPEAT
BtaA
BtaB
00564 7/19/21
(c) Kanehisa Laboratories

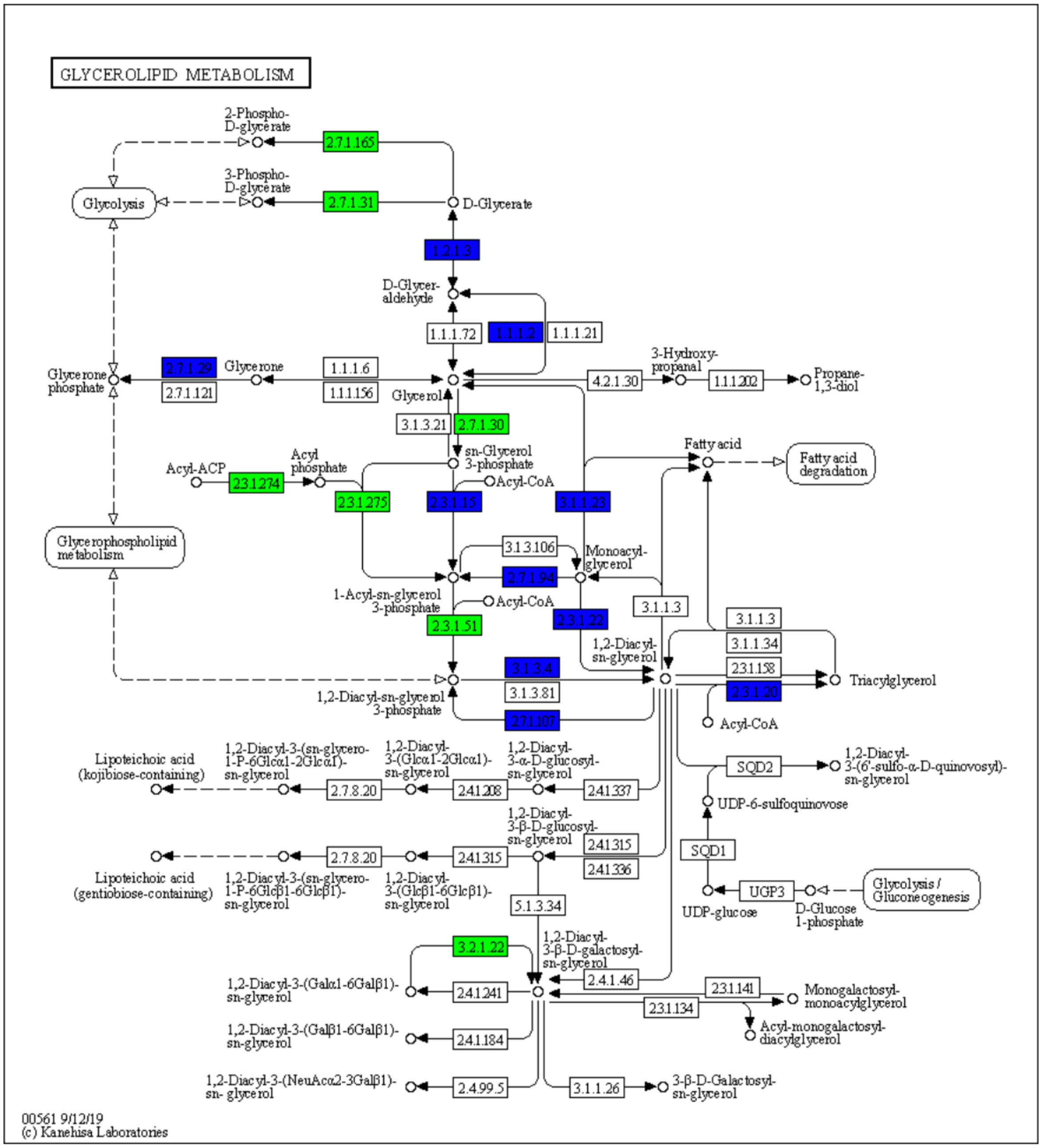
GLYCEROLIPID METABOLISM
2-Phospho-D-glycerate
2.7.1.165
3-Phospho-D-glycerate
2.7.1.31
Glycolysis
D-Glycerate
1.2.1.3
D-Glycer-aldehyde
1.1.1.72
1.1.1.2
1.1.1.21
Glycerone phosphate
2.7.1.29
2.7.1.121
Glycerone
1.1.1.6
1.1.1.156
Glycerol
3-Hydroxy-propanal
4.2.1.30
1.1.1.202
Propane-1,3-diol
3.1.3.21
2.7.1.30
sn-Glycerol 3-phosphate
Acyl-ACP
Acyl phosphate
2.3.1.274
2.3.1.275
Acyl-CoA
2.3.1.15
3.1.1.23
Fatty acid
Fatty acid degradation
Glycerophospholipid metabolism
3.1.3.106
Monoacyl-glycerol
1-Acyl-sn-glycerol 3-phosphate
2.7.1.94
Acyl-CoA
2.3.1.51
2.3.1.22
3.1.1.3
3.1.1.3
3.1.1.34
1,2-Diacyl-sn-glycerol
2.3.1.158
3.1.3.4
3.1.3.81
2.3.1.20
Triacylglycerol
1,2-Diacyl-sn-glycerol 3-phosphate
2.7.1.107
Acyl-CoA
Lipoteichoic acid (kojibiose-containing)
1,2-Diacyl-3-(sn-glycero-1-P-6Glcα1-2Glcα1)-sn-glycerol
1,2-Diacyl-3-(Glcα1-2Glcα1)-sn-glycerol
1,2-Diacyl-3-α-D-glucosyl-sn-glycerol
2.7.8.20
2.4.1.208
2.4.1.337
SQD2
1,2-Diacyl-3-(6'-sulfo-α-D-quinovosyl)-sn-glycerol
UDP-6-sulfoquinovose
1,2-Diacyl-3-β-D-glucosyl-sn-glycerol
2.4.1.315
2.4.1.336
SQD1
2.7.8.20
2.4.1.315
Lipoteichoic acid (gentiobiose-containing)
1,2-Diacyl-3-(sn-glycero-1-P-6Glcβ1-6Glcβ1)-sn-glycerol
1,2-Diacyl-3-(Glcβ1-6Glcβ1)-sn-glycerol
5.1.3.34
UGP3
UDP-glucose
D-Glucose 1-phosphate
Glycolysis / Gluconeogenesis
3.2.1.22
1,2-Diacyl-3-β-D-galactosyl-sn-glycerol
2.4.1.46
1,2-Diacyl-3-(Galα1-6Galβ1)-sn-glycerol
2.4.1.241
2.3.1.141
Monogalactosyl-monoacylglycerol
2.3.1.134
Acyl-monogalactosyl-diacylglycerol
1,2-Diacyl-3-(Galβ1-6Galβ1)-sn-glycerol
2.4.1.184
1,2-Diacyl-3-(NeuAcα2-3Galβ1)-sn- glycerol
2.4.99.5
3.1.1.26
3-β-D-Galactosyl-sn-glycerol
00561 9/12/19
(c) Kanehisa Laboratories

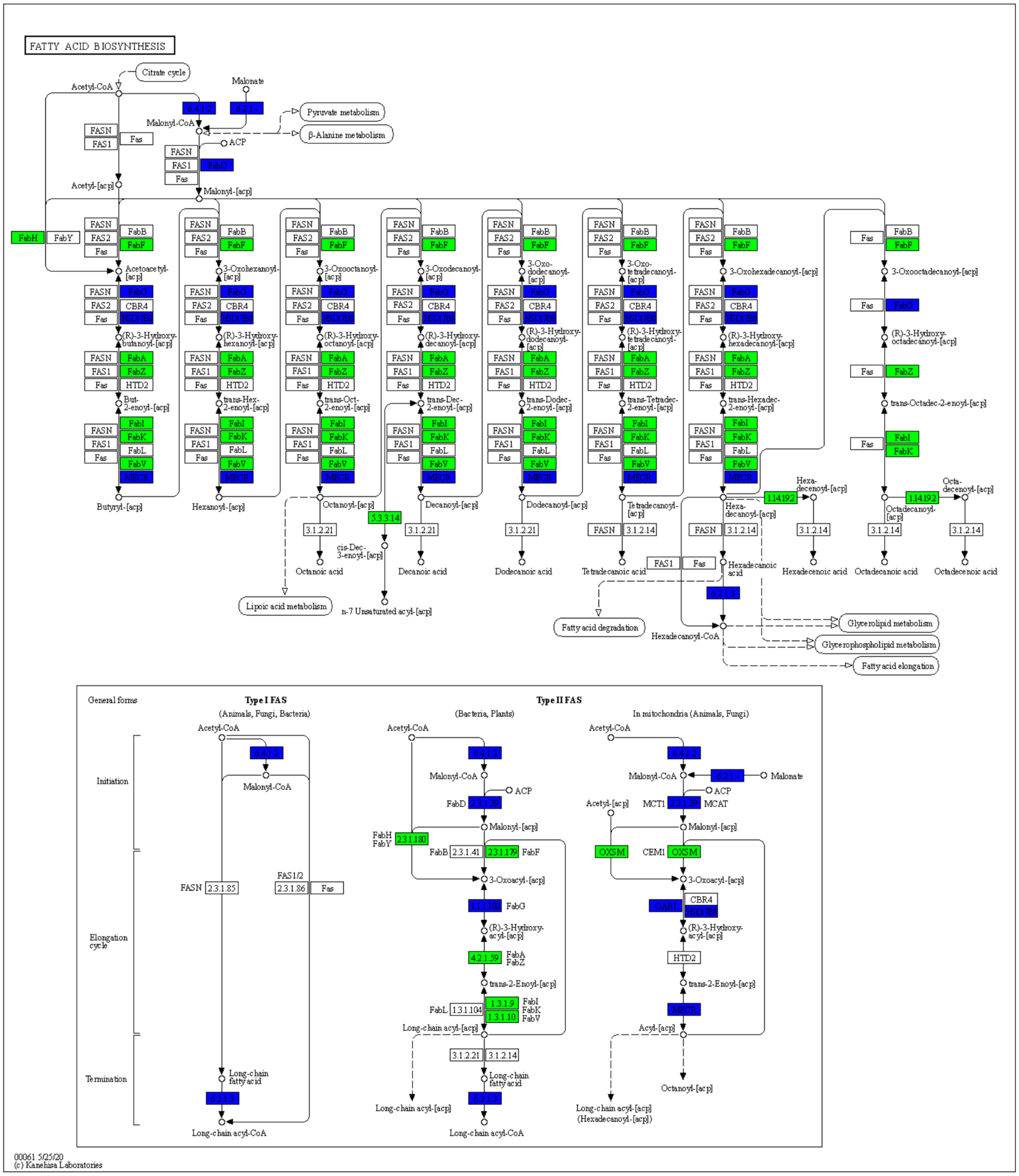

FATTY ACID BIOSYNTHESIS
Citrate cycle
Acetyl-CoA
Malonate
Malonyl-CoA
Pyruvate metabolism
β-Alanine metabolism
ACP
Acetyl-[acp]
Malonyl-[acp]
Acetoacetyl-[acp]
3-Oxohexanoyl-[acp]
3-Oxooctanoyl-[acp]
3-Oxodecanoyl-[acp]
3-Oxo-dodecanoyl-[acp]
3-Oxo-tetradecanoyl-[acp]
3-Oxohexadecanoyl-[acp]
3-Oxooctadecanoyl-[acp]
(R)-3-Hydroxy-butanoyl-[acp]
(R)-3-Hydroxy-hexanoyl-[acp]
(R)-3-Hydroxy-octanoyl-[acp]
(R)-3-Hydroxy-decanoyl-[acp]
(R)-3-Hydroxy-dodecanoyl-[acp]
(R)-3-Hydroxy-tetradecanoyl-[acp]
(R)-3-Hydroxy-hexadecanoyl-[acp]
(R)-3-Hydroxy-octadecanoyl-[acp]
But-2-enoyl-[acp]
trans-Hex-2-enoyl-[acp]
trans-Oct-2-enoyl-[acp]
trans-Dec-2-enoyl-[acp]
trans-Dodec-2-enoyl-[acp]
trans-Tetradec-2-enoyl-[acp]
trans-Hexadec-2-enoyl-[acp]
trans-Octadec-2-enoyl-[acp]
Butyryl-[acp]
Hexanoyl-[acp]
Octanoyl-[acp]
Decanoyl-[acp]
Dodecanoyl-[acp]
Tetradecanoyl-[acp]
Hexa-decanoyl-[acp]
Hexa-decenoyl-[acp]
Octadecanoyl-[acp]
Octa-decenoyl-[acp]
FabH
FabY
FabB
FabF
FabA
FabZ
FabI
FabK
FabV
FAS1
5.3.3.14
1.14.19.2
3.1.2.21
3.1.2.14
cis-Dec-3-enoyl-[acp]
Octanoic acid
Decanoic acid
Dodecanoic acid
Tetradecanoic acid
Hexadecanoic acid
Hexadecenoic acid
Octadecanoic acid
Octadecenoic acid
Lipoic acid metabolism
n-7 Unsaturated acyl-[acp]
Fatty acid degradation
Hexadecanoyl-CoA
Glycerolipid metabolism
Glycerophospholipid metabolism
Fatty acid elongation
General forms
Type I FAS
(Animals, Fungi, Bacteria)
Type II FAS
(Bacteria, Plants)
In mitochondria (Animals, Fungi)
Initiation
Elongation cycle
Termination
FASN
2.3.1.85
FAS1/2
2.3.1.86
Long-chain fatty acid
Long-chain acyl-CoA
FabD
FabH
FabY
2.3.1.180
FabB
2.3.1.41
2.3.1.179
FabF
FabG
3-Oxoacyl-[acp]
(R)-3-Hydroxy-acyl-[acp]
4.2.1.59
FabA
FabZ
trans-2-Enoyl-[acp]
FabL
1.3.1.104
1.3.1.9
1.3.1.10
FabI
FabK
FabV
Long-chain acyl-[acp]
3.1.2.21
3.1.2.14
Acetyl-[acp]
MCT1
MCAT
OXSM
CEM1
CBR4
HTD2
Acyl-[acp]
Octanoyl-[acp]
Long-chain acyl-[acp]
(Hexadecanoyl-[acp])
00061 5/25/20
(c) Kanehisa Laboratories
