# Supplementary figures and images for: Author Correction: Proximity ligation strategy for the genomic reconstruction of microbial communities associated with the ectoparasite Caligus rogercresseyi
Source: Sci Rep. 2022 May 9;12:7530. doi: 10.1038/s41598-022-11969-0 (PMC9085864; doi:10.1038/s41598-022-11969-0)

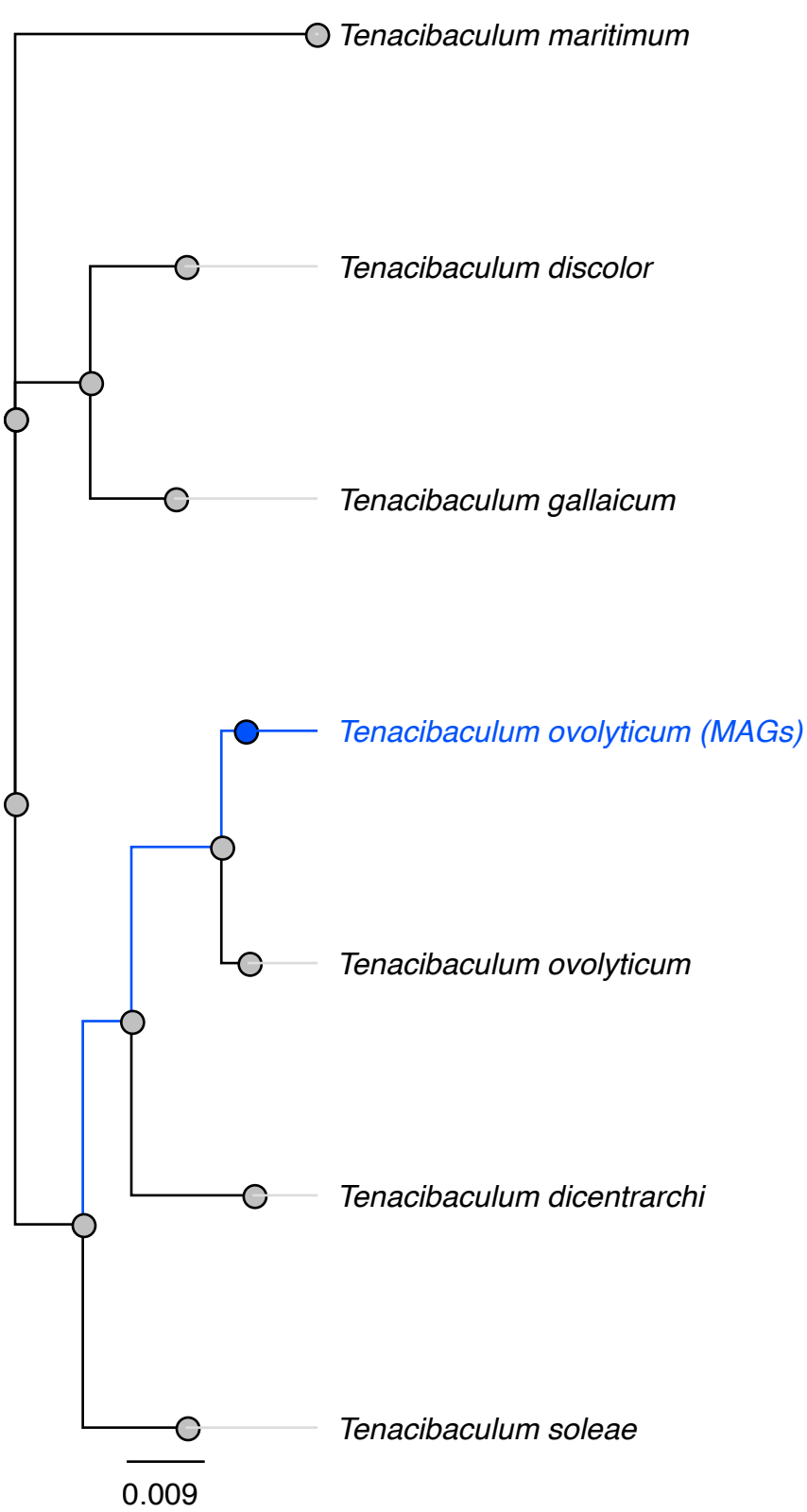

Supplement: Supplementary file 1 — Supplementary Information. [file 41598_2022_11969_MOESM1_ESM.pdf]
